# Long‐Read Reference Transcriptomes for Five Species of Rosaceae Tribe Spiraeeae: A Resource for Investigating Sequence Divergence, Species‐Associated Expression Variation, and Leaf‐Developmental Candidate Genes

**DOI:** 10.1002/pld3.70202

**Published:** 2026-11-02

**Authors:** Armin M. Mafakheri, Daniel Potter

**Affiliations:** ^1^ Department of Plant Sciences University of California ‐ Davis Davis California USA

**Keywords:** comparative transcriptomics, differentially expressed transcripts, gene coexpression networks, purifying selection

## Abstract

Species in the Rosaceae tribe Spiraeeae occupy diverse habitats and vary strikingly in growth form and leaf morphology. Still, no reference transcriptomes have been published for the tribe, and its transcriptome‐level variation is uncharacterized. We generated long‐read (PacBio Iso‐Seq) reference transcriptomes of five species spanning three genera: *Petrophytum caespitosum* (Nutt.) Rydb., *Spiraea lucida* Douglas ex Greene, *Spiraea splendens* Baumann ex K. Koch, *Aruncus dioicus* (Walter) Fernald var. *dioicus*, and *Aruncus aethusifolius* (H.Lév.) Nakai. We then quantified expression across 20 individuals using 3′‐tag RNA sequencing (TagSeq) to characterize expression patterns, ortholog sequence divergence, and gene repertoire. Multivariate analyses (principal component analysis [PCA] and hierarchical clustering) resolved replicates into three groups corresponding to the sampled genera. Because the material combined field‐collected and potted plants, we treat expression comparisons as descriptive and do not attribute them to genetic control of interspecific differences. Pairwise analyses revealed extensive transcriptional divergence between genera, including thousands of differentially expressed transcripts in comparisons such as *A. aethusifolius* vs. *P. caespitosum* . Functional enrichment highlighted variation in transport and catabolism, plastid organization, and biotic‐stress responses, implicating both metabolic and organelle‐associated processes. Transcription factor families, particularly bHLH and bZIP, varied most among differentially expressed transcripts. Weighted gene coexpression network analysis (WGCNA) recovered modules correlated with species identity; because between‐species variation dominates this dataset, we treat these as descriptive summaries of coexpression structure and do not regard them as independent evidence of species‐specific regulatory programs. Because reads were quantified against a reference assembled from species‐specific contigs, mapping rates were higher within species (70.5%–85.1%) than across species (25.1%–77.0%), and between‐species expression comparisons are interpreted with this asymmetry in mind. Sequence‐level analysis of orthologous transcript pairs indicated that protein‐coding genes are predominantly under purifying selection. A small fraction (2.7%–5.0% of retained pairs) showed Ka/Ks > 1, but these were concentrated at low synonymous divergence, where ratio estimates are unstable; we therefore treat them as screening‐level candidates for rapid divergence, enriched for stress‐response, membrane‐trafficking, and ion‐transport annotations, and we make no claim of positive selection. These results provide a comparative transcriptomic framework for Spiraeeae and identify candidate transcripts and protein‐coding differences for future studies of ecological and morphological variation in Rosaceae.

## 1 Introduction

Comparative transcriptomics provides a powerful framework for characterizing differences in transcript abundance among closely related species, especially in nonmodel plant lineages lacking reference genomes. By comparing transcript abundance, transcript structure, and coexpression patterns across taxa, this approach can identify lineage‐associated molecular differences and generate hypotheses about the biological processes associated with ecological and morphological divergence (Haas et al. 2013; Ward et al. 2012). In addition, ortholog‐based sequence analyses can be used to examine coding‐sequence divergence among homologous genes in different species after lineage splitting.

Comparative transcriptomic analyses across related plant lineages have been widely used to characterize expression divergence and to identify candidate genes associated with lineage differentiation. Examples include comparisons of domesticated and wild tomato lineages (Koenig et al. 2013) and phylogenomic analyses of Caryophyllales (Yang et al. 2015) and comparative studies in bamboo, tea, *Camellia*, and *Thuja* that identified lineage‐associated patterns of functional enrichment and orthologs with elevated sequence divergence (Chang et al. 2023; Wu et al. 2022; Xia et al. 2014; Zhao et al. 2013). These studies illustrate the utility of transcriptome‐scale comparisons for describing molecular differences among related taxa, even when direct functional validation is not yet available.

Despite these broad advances, many plant lineages remain underexplored from a comparative transcriptomic perspective. The focus of this study, tribe Spiraeeae of the large, economically important family Rosaceae, is an example. Although research has extensively explored the genetic makeup of economically significant members of Rosaceae, such as apples (Gul et al. 2019), strawberries (Qiao et al. 2016), and pears (Zhao et al. 2019), there is a notable scarcity of in‐depth genetic studies on many wild or less commercially prominent species. This lack of comprehensive genetic data limits our ability to compare transcriptomic and sequence‐level variation across the family's full ecological and morphological diversity. Furthermore, many candidate genes and pathways associated with stress‐related and lineage‐specific differences in wild Rosaceae species remain insufficiently characterized, highlighting an important area for future comparative work.

Within Rosaceae, the tribe Spiraeeae (subfamily Amygdaloideae) is a well‐supported clade comprising nine genera and 65–100 species that collectively exhibit a remarkable morphological and ecological diversity (Table S1). Previously Potter et al. (2007) divided the tribe into two subclades, one comprising *Aruncus*, *Luetkea*, *Holodiscus*, and *Xerospiraea*, the other including the remaining genera. The genetic basis underlying leaf morphology and ecological differentiation within tribe Spiraeeae remains poorly understood. Although the clade exhibits striking variation in leaf form and habitat preference, ranging from mesic forest understories to dry rocky outcrops, few studies have examined how transcript expression, transcript structure, and regulatory networks vary among Spiraeeae species. As a result, the molecular mechanisms linking transcriptomic variation to morphological and ecological diversity in this group remain largely unresolved.

Here, we generated reference transcriptomes for five species from three genera of Spiraeeae (Table S2), described species‐associated differences in transcript abundance, and nominated candidate genes involved in leaf development for future testing. Because leaf morphology is confounded with taxon in this sampling design and no quantitative morphometric measurements were made, we do not attempt to associate expression differences with leaf form.

The leaf morphologies of the five species sampled here range from large and highly compound with toothed leaflet blades (*Aruncus*) to moderately small and simple leaves with toothed margins (*Spiraea*) to very small and entire leaves (*Petrophytum*). The selected taxa also represent a broad ecological gradient within Spiraeeae. *P. caespitosum* is associated with dry, exposed rocky substrates, whereas *A. dioicus* typically occupies mesic habitats such as forest margins and openings. Within *Spiraea*, *S. lucida* is found in relatively drier to mesic habitats, whereas *S. splendens* is associated with wetter habitats, including streamsides and marshy alpine meadows. By sampling species that span this gradient of water availability and growth form, our goal was not to test a specific ecophysiological trait directly, but rather to identify transcriptomic patterns and candidate pathways that may help frame future hypothesis‐driven studies of ecological and morphological differentiation (Hummer and Janick 2009; Illa et al. 2011; Xiang et al. 2017). Together, these attributes make Spiraeeae a useful system for comparative studies of molecular variation associated with lineage diversification, morphological diversity, and habitat differentiation.

We sequenced, assembled de novo, and annotated long‐read transcriptomes for the five species. In this study, we analyze orthologous transcript pairs to estimate synonymous substitution rates (Ks), nonsynonymous substitution rates (Ka), and Ka/Ks ratios as standard comparative metrics for assessing relative sequence divergence and identifying candidate loci under differential selective constraints (Byrne and Wolfe 2007; Fay and Wu 2003; Greenberg et al. 2006; Semon and Wolfe 2007, 2008) in Spiraeeae. These analyses are used here to complement transcript‐level expression comparisons; they are not intended to infer genes under positive selection or to establish causal mechanisms underlying species differences.

This study had two complementary objectives: first, to describe species‐associated differences in transcript abundance and to nominate leaf‐developmental candidate genes for future testing; and second, to integrate expression‐based analyses with comparative sequence‐level metrics to identify candidate genes and networks showing elevated coding‐sequence divergence. Together, these approaches allow us to move from transcriptome‐wide patterns to species‐specific candidates and coordinated gene networks that may be relevant to morphological and ecological differentiation within Spiraeeae.

## 2 Materials and Methods

### 2.1 Plant Material and RNA Isolation

Plant materials used in this study included 3–6 biological replicates (individuals) of each species (Table S2) harvested either from plants growing in the field ( *P. caespitosum* subsp. *caespitosum*, *S. splendens* ) or from potted plants (*Aruncus aethusifolius*, *A. dioicus* , and *S. lucida* ). Accordingly, differences in expression should be interpreted primarily as species‐associated patterns in this dataset, while acknowledging that environmental/source effects cannot be fully disentangled. Replication varied among species (3–6 individuals) due to differences in plant availability and the retention of only samples meeting stringent RNA quality criteria for sequencing and downstream expression analyses. Field collections of *P. caespitosum* subsp. *caespitosum* and *S. splendens* were made in Salmon–Challis National Forest, Idaho and Lassen National Forest, California, respectively. Collection permits were not required because neither species is listed as threatened, endangered, or sensitive. In both areas, permits are not required for the collection of small quantities of nontimber forest products, other than mushrooms, for personal, noncommercial use. The three to five youngest visible leaves at the shoot apex were harvested from each plant and stored in RNAlater TissueProtect Tubes (Qiagen) until transfer to a −80°C freezer. Sampling was standardized by leaf position rather than by a measured developmental index. Because field‐collected and potted plants were not phenologically synchronized, developmental stage cannot be regarded as controlled across species.

RNA was isolated using a Cetyltrimethyl Ammonium Bromide (CTAB)‐based extraction protocol as described in Blanco‐Ulate et al. (2013). RNA purity was evaluated with a NanoDrop 2000 spectrophotometer (Thermo Scientific, Hanover Park, IL, USA). RNA was quantified using a Qubit 2.0 Fluorometer with the RNA broad‐range kit (Life Technologies, Carlsbad, CA, USA). RNA integrity was assessed using electrophoresis and an Agilent 2100 Bioanalyzer (Agilent Technologies, Santa Clara, CA, USA). Only RNA with an integrity number (RIN) greater than 8.0 was used for library preparation. RNA from all 20 individuals was used for 3′‐tag RNA sequencing (TagSeq); RNA from one representative individual per species (Aa7, Ad7, P10, Sl7, and Ss1) was additionally used for Iso‐Seq library preparation.

### 2.2 Iso‐Seq Library Preparation and Sequencing

Iso‐Seq libraries were used solely to construct the reference transcriptome; expression was quantified separately from TagSeq libraries (see below). For each species, 1 μg of RNA from a single representative individual was used for cDNA synthesis and for SMRTbell library construction using the SMARTer PCR cDNA synthesis kit (Clontech Laboratories Inc., Mountain View, CA, USA). First‐strand cDNA synthesis was performed using the SMARTScribe Reverse Transcriptase (Clontech Laboratories Inc., Mountain View, CA, USA). To minimize artifacts during large‐scale amplification, a cycle optimization step was performed by collecting five 5‐μL aliquots at 10, 12, 14, 16, and 18 PCR cycles. PCR reaction aliquots were loaded onto a 0.8% E‐Gel precast agarose gel (Invitrogen, Life Technologies, Carlsbad, CA, USA) to determine the optimal cycle number. Second‐strand cDNA was synthesized and amplified using the Kapa HiFi PCR kit (Kapa Biosystems, Wilmington, MA, USA) with the 5′ PCR Primer II A (Clontech Laboratories Inc., Mountain View, CA, USA) following the manufacturer's instructions. Large‐scale PCR was performed using the number of cycles determined during the optimization step (14 cycles). Species‐specific barcoded double‐stranded cDNAs (barcodes bc1001, bc1002, bc1004, bc1005, and bc1006) were pooled at equal amounts and used for size selection. Size selection was performed using a BluePippin (Sage Science, Beverly, MA, USA), and 1–2‐kbp, 2–3‐kbp, 3–6‐kbp, and 5–10‐kbp fractions were collected. After size selection, each fraction was PCR‐enriched before SMRTbell template library preparation. cDNA SMRTbell libraries were prepared using 1–3‐μg cDNA of PCR‐enriched, size‐selected samples, followed by DNA damage repair and SMRTbell ligation using the SMRTbell Template Prep Kit 1.0 (Pacific Biosciences, Menlo Park, CA, USA). A second size selection was performed on the 3–6‐kbp and 5–10‐kbp fractions to remove short contaminating SMRTbell templates. The pooled barcoded library was sequenced on a single SMRT cell on a PacBio Sequel II system (DNA Technologies Core, University of California, Davis, USA), yielding 3,599,918 circular consensus sequence (CCS) reads (8.11 Gbp; mean read length 2253 bp; median read quality Q40). Demultiplexing, filtering, quality control, clustering, and polishing of the Iso‐Seq sequencing data were performed using SMRT Link v8.0; refinement was carried out without requiring a polyA tail, and clustering was performed separately for each species. Demultiplexing assigned 848,223 reads to *A. aethusifolius*, 596,408 to *A. dioicus* , 928,092 to *P. caespitosum* , 639,089 to *S. lucida* , and 334,053 to *S. splendens* . Coding sequences (CDSs) were identified using TransDecoder v5.5.0 (https://github.com/TransDecoder/TransDecoder, accessed on November 1, 2022) with the default parameters.

The assembled sequences for each species were clustered with CD‐HIT v4.8.1 (Fu et al. 2012). Sequences with similarity > 95% within each species were grouped into one class, and the longest sequence in each class was treated as a representative nonredundant transcript for downstream processing. Assembly statistics were obtained using BUSCO 5.8.2 (Manni et al. 2021) (organism embryophyta_odb10).

### 2.3 TagSeq Library Preparation and Sequencing

Expression was quantified from 3′‐tag RNA sequencing (TagSeq) libraries prepared from all 20 individuals (*A. aethusifolius n* = 6, *A. dioicus* *n* = 3, *P. caespitosum* *n* = 5, *S. lucida* *n* = 3, and *S. splendens* *n* = 3) at the DNA Technologies Core, University of California, Davis. Libraries were sequenced single‐end (93 bp) on an Illumina HiSeq 4000. Raw reads were preprocessed with HTStream v1.3.2 to remove PhiX contamination, adapter sequences, and low‐quality bases, with a minimum retained read length of 50 bp. All 20 libraries were prepared in a single batch and sequenced in a single Illumina HiSeq 4000 run, so library preparation and sequencing batch are not confounded with species.

### 2.4 Functional Annotation of Transcripts

Trinotate was used to functionally annotate representative nonredundant transcripts and ORFs (Bryant et al. 2017). Trinotate makes use of several different well‐referenced methods for functional annotation, including National Center for Biotechnology Information (NCBI) nucleotide sequences database (NT), NCBI nonredundant database (NR), Universal protein (UniProt) database, Protein family (PFAM), and KEGG Orthology (KO). Eukaryotic Orthologous Group (KOG) assignments were taken from the eggNOG component of the Trinotate output.

### 2.5 Exploratory Expression Analysis (Principal Component Analysis [PCA] and Hierarchical Clustering)

Exploratory analyses were conducted to assess global transcript expression patterns across samples before differential expression testing. Preprocessed TagSeq reads were quantified against the combined nonredundant transcript reference (85,404 transcripts) using Salmon v1.10.0 in mapping‐based (quasimapping) mode, run in single‐end mode. Mapping rates ranged from 70.5% to 85.1% across samples. Salmon‐estimated read counts (NumReads) from the quant.sf files were combined into a transcript‐by‐sample count matrix in R; because TagSeq generates 3′‐biased reads whose coverage is not distributed across the transcript body, raw counts rather than length‐normalized TPM values were carried forward. Count data were imported into edgeR v4 (Robinson et al. 2010) to construct a DGEList object; lowly expressed transcripts were removed using filterByExpr, and library size normalization was performed using the trimmed mean of M‐values (TMM) method. Log2 counts‐per‐million (logCPM) values were computed from the TMM‐normalized object and used for exploratory visualization. The top 500 most variably expressed transcripts across all samples were selected based on variance in TMM‐normalized expression values. Hierarchical clustering was performed using Euclidean distance and visualized as a heatmap with expression values scaled by row *Z*‐scores (Figure 3). PCA was performed on the same set of TMM‐normalized expression values, with each point representing an individual biological replicate. These analyses were used solely for exploratory visualization of transcriptome‐wide structure and were not used for statistical inference.

### 2.6 Analysis of Differentially Expressed Transcripts (DETs)

To investigate transcript expression differences between species, Iso‐Seq transcripts from all five species were combined and clustered with CD‐HIT (Fu et al. 2012) to generate a single nonredundant reference transcriptome. TagSeq reads from each of the 20 individuals were then quantified against this combined reference using Salmon in mapping‐based mode, thereby estimating relative transcript abundance across species within a shared‐reference framework. Because the reference is assembled from species‐specific Iso‐Seq contigs, mapping rates were consistently higher when samples were quantified against their own species' transcripts (70.5%–85.1%) than against those of other species (25.1%–77.0%); this asymmetry is a property of the shared‐reference design and is considered when interpreting between‐species comparisons.

We did not apply reciprocal mapping filters, sequence‐similarity thresholds, or an ortholog‐constrained count matrix to the expression data. An ortholog‐constrained matrix would necessarily be restricted to reciprocal‐best‐hit pairs recoverable in all five species—a set substantially smaller than the full reference and itself biased by the uneven assembly completeness documented above. We therefore restricted ortholog‐level inference to the sequence‐divergence analyses, which operate directly on reciprocal best BLASTP hits (RBH) pairs, and interpret the expression results as relative abundance within a shared‐reference framework rather than as ortholog‐level expression comparisons.

The quantification of transcript expression was performed using raw read counts obtained from Salmon, which were imported into the edgeR v4 R package (Robinson et al. 2010) for downstream processing. Library size normalization was performed in edgeR using the TMM method to account for differences in sequencing depth across samples, and lowly expressed transcripts were filtered before testing. Differential expression was then assessed using the limma–voom framework, in which voom was applied to model the mean–variance relationship of the filtered count data and generate precision weights, followed by fitting linear models under a species‐only design (~0 + species) and testing specified pairwise contrasts among the five species. DETs were identified by comparing the five species using TagSeq data, with 3–6 biological replicates per species. The analysis accounted for transcript isoform complexity by quantifying transcript‐level expression relative to the combined Iso‐Seq–derived reference transcriptome. Significant DETs were identified using the Benjamini–Hochberg false discovery rate (FDR) procedure to adjust *p*‐values for multiple testing, together with a minimum log2 fold‐change threshold to ensure robust and reliable results. Differential expression analyses were interpreted as relative expression differences derived from mapping each species to a shared‐reference transcriptome: they are not direct ortholog‐level expression comparisons. This approach enables comparative transcriptomic analyses across nonmodel species by pairing short‐read expression measurement with the full‐length isoform resolution of the long‐read reference. Consequently, the magnitudes of between‐species expression differences reported here are expected to be inflated relative to true biological divergence, and comparisons between genera are more affected than comparisons within a genus, where reference representation is more even.

Ortholog‐level sequence analyses were performed separately using reciprocal‐best‐hit approaches and Ka/Ks estimation and were not inferred directly from expression data. All statistical analyses and data visualizations related to differential expression were performed in R using RStudio. Volcano plots were generated using the ggplot2 R package (Wickham 2016). Venn diagrams for the four focal pairwise comparisons were generated using the ggVennDiagram R package (Gao et al. 2021) and overlap among DET sets was visualized using the UpSetR R package (Conway et al. 2017).

### 2.7 Gene Ontology (GO) Enrichment Analysis of DETs and Identification of Common TFs

Functional enrichment of the up‐ and downregulated DETs from each pairwise comparison was performed with ShinyGO 0.80 (Ge et al. 2020) using its STRING‐based enrichment option (Szklarczyk et al. 2023), with *Arabidopsis thaliana* (NCBI taxonomy ID 3702) as the reference organism and the default whole‐genome background. Enrichment was assessed for Gene Ontology biological process, molecular function, and cellular component terms, and for KEGG pathways, with significance based on the reported false discovery rate. Transcripts were mapped to *A. thaliana* identifiers via their best Swiss‐Prot hits from the Trinotate annotation. The list of most common transcription factors (TFs) and their variability across different comparisons was obtained from the plant TF database PlantTFDB 5.0 (Tian et al. 2020).

### 2.8 Weighted Gene Coexpression Network Analysis (WGCNA)

WGCNA was performed in R using the WGCNA package (Langfelder and Horvath 2008). Before network construction, the top 5000 most variably expressed transcripts were selected based on median absolute deviation of normalized expression values. This variance‐based filtering step was applied to reduce noise and computational complexity and does not imply that all selected transcripts are directly involved in the biological processes under investigation. Pairwise Pearson correlations between genes were used to construct an adjacency matrix using a soft‐thresholding power of 30 selected from candidate powers of 1–10 and 12–30, with a signed hybrid network type. The adjacency matrix was transformed into a topological overlap matrix (TOM), and genes were hierarchically clustered on TOM dissimilarity. Modules were identified by dynamic tree cutting (deepSplit = 2, pamRespectsDendro = FALSE, minimum module size = 30) and merged at an eigengene dissimilarity threshold of 0.25, with unassigned genes designated to the gray module. Module eigengenes were calculated as the first principal component of each module's expression matrix. Soft‐thresholding power selection was guided by scale‐free topology fit and mean connectivity diagnostics implemented in WGCNA (Figure S9). Module–trait relationships were assessed by correlating module eigengenes with plant species identity, and results were visualized using a module–trait correlation heatmap (Figure S11). Modules showing a strong positive correlation (correlation > 0.5, *p* < 0.05) were selected for downstream analyses. Selected modules were exported to Cytoscape (Shannon et al. 2003), where hub genes were identified using the CytoHubba plugin based on degree centrality. From each of the important modules, the top 20 transcripts were annotated against known databases.

### 2.9 Ortholog Identification and Screening for Candidate Loci With Elevated Ka/Ks Ratios

Putative orthologs between the 10 pairwise comparisons of the five species were identified using RBH, with an E‐value cutoff of 1.0 × 10^−10^ (Moreno‐Hagelsieb and Latimer 2008). For each orthologous pair, the corresponding CDSs predicted by TransDecoder were retrieved and aligned at the nucleotide level using LASTZ. The YN algorithm, implemented in KaKs_Calculator v1.2 (Zhang et al. 2006), was used to calculate the nonsynonymous substitution rate (Ka), the synonymous substitution rate (Ks), and the Ka/Ks ratio for each orthologous gene pair. Ortholog pairs with Ks > 2 were excluded from downstream analyses. To reduce noise from unstable rate estimates associated with very recent divergence, ortholog pairs with Ks < 0.05 were also excluded from inference. Orthologous loci with Ka/Ks ratios greater than 1 were flagged for comparative screening and visualized using the ggplot2 R package (Wickham 2016). Because a pairwise, gene‐wide Ka/Ks ratio averages over all codons and does not by itself constitute a formal test of positive selection, and because per‐pair ratios are unstable at low Ks, these loci are reported as screening candidates for rapid sequence divergence; demonstrating positive selection would require formal codon‐based tests (e.g., site or branch‐site models) (Yang and Nielsen 2002).

### 2.10 Leaf‐Developmental Gene Identification and Focused Ka/Ks Estimation

Core leaf‐developmental regulators were identified from the Swiss‐Prot/UniProt annotation of the combined nonredundant reference transcriptome (Trinotate; translated‐nucleotide search, E < 1 × 10^−5^; described above) by screening for the transcription‐factor families that pattern leaf shape, size, and polarity: CIN‐type TCP, GROWTH‐REGULATING FACTOR (GRF) with its GIF/AN3 coactivators, YABBY, HD‐ZIP III/REVOLUTA, and the BELL and WOX homeobox families. Each match was recorded as gene‐specific or family‐level according to its best hit identity, and transcripts with a best hit identity ≥ 55% to the named regulator were retained for focused sequence‐divergence analysis. For each family, one representative transcript per species was used to form cross‐species ortholog pairs. Protein sequences were aligned pairwise (BLOSUM62) and used to construct codon‐level alignments of the corresponding CDSs; Ka, Ks, and Ka/Ks were then estimated by the Nei–Gojobori method with Jukes–Cantor correction (Nei and Gojobori 1986) in a custom R script (Biostrings), available in the GitHub repository. Pairs with alignment identity < 40% or Ks ≥ 2 were excluded. This simpler estimator was used for the focused analysis because it was applied to a small set of highly conserved regulators for which the conclusion (Ka/Ks ≪ 1) is insensitive to the choice of method.

### 2.11 Accession Numbers

Sequence data from this article can be found in the NCBI Sequence Read Archive under BioProject accession number PRJNA1394637. Assembled reference transcriptomes have been deposited at DDBJ/ENA/GenBank as Transcriptome Shotgun Assembly projects under accessions GLQD00000000 (A. aethusifolius Aa7), GLQC00000000 ( *A. dioicus* Ad7), GLQE00000000 ( *P. caespitosum* P10), GLQF00000000 ( *S. lucida* Sl7) and GLQG00000000 ( *S. splendens* Ss1). UniProt/SwissProt accession numbers for the best hit leaf‐developmental regulators are listed in Table S5.

## 3 Results

### 3.1 De Novo Assembly and Functional Annotation of Transcriptome

Full‐length reads represent CCS reads spanning the complete transcript from the 5′ primer to the 3′ poly(A) tail, whereas full‐length nonchimeric (FLNC) reads correspond to full‐length reads that do not contain evidence of transcript chimerism. Transcript clustering was performed to collapse redundant FLNC reads into nonredundant transcript clusters, which approximate unique transcript isoforms rather than gene counts.

*A. aethusifolius* yielded the highest number of full‐length reads (847,960) and 59,679 nonredundant transcripts, whereas *S. splendens* , despite having the fewest full‐length reads (333,890), still produced 31,518 transcripts. *A. dioicus* and *P. caespitosum* were characterized by high read counts (596,112 and 927,885 full‐length reads; 572,376 and 924,652 FLNC, respectively) but differed in transcript numbers, reflecting differences in sequencing depth, transcript diversity, or clustering behavior among species. *S. lucida* contained the longest observed transcript (8754 bp), indicating the presence of exceptionally long transcript isoforms in this species. Across species, median transcript lengths ranged from 1607 to 2315 bp (Table 1).

**TABLE 1:** Iso‐Seq read counts and reference transcriptome statistics for the five species. Full‐length (num_reads_fl) and full‐length nonchimeric (num_reads_flnc) read counts are taken from the Iso‐Seq demultiplexing and refine reports. Transcripts, length quantiles (minimum, first quartile, median, mean, third quartile, and maximum) and N50 were computed from the clustered, nonredundant reference transcriptome assembled for each species, the same assemblies used for BUSCO assessment (Figure 1) and deposited in the Transcriptome Shotgun Assembly archive. All lengths are given in bp.

Species	num_reads_fl	num_reads_flnc	Transcripts	Min	1st Qu.	Median	Mean	3rd Qu.	Max.	N50
*A. aethusifolius*	847,960	845,211	59,679	59	1666	2315	2431.1	3100	8595	2790
*A. dioicus*	596,112	572,376	67,445	51	932	1607	1683.7	2311	7638	2177
*P. caespitosum*	927,885	924,652	71,101	132	1584	2146	2273.7	2821	8526	2548
*S. lucida*	638,956	636,350	56,736	59	1276	1779	1957.0	2440	8754	2217
*S. splendens*	333,890	329,660	31,518	77	1290	1808	1934.2	2452	8197	2209

*Note:* Transcripts shorter than 200 bp (50 in *A. aethusifolius*, 2420 in *A. dioicus* , 14 in *P. caespitosum* , 74 in *S. lucida*, and 38 in *S. splendens* ) were removed prior to TSA submission, giving deposited assembly sizes of 59,629, 65,025, 71,087, 56,662, and 31,480 transcripts, respectively.

Transcriptome completeness was assessed with BUSCO v5.8.2 in transcriptome mode against the embryophyta_odb10 lineage (*n* = 1614 BUSCO groups). Four of the five assemblies were closely comparable in completeness: *A. dioicus* 82.8% (S 32.5%, D 50.3%), *P. caespitosum* 82.8% (S 24.0%, D 58.8%), *A. aethusifolius* 81.8% (S 22.2%, D 59.6%), and *S. lucida* 81.7% (S 22.6%, D 59.2%). *S. splendens* was appreciably less complete at 71.4% (S 34.0%, D 37.4%), with correspondingly more missing BUSCOs (26.6% versus 14.3%–17.0% for the other species; Figure 1). This difference tracks sequencing depth: *S. splendens* received 334,053 of the 3.35 million demultiplexed Iso‐Seq reads, approximately one‐third the yield obtained for *P. caespitosum* , and yielded 31,518 transcripts compared with 56,736–71,101 for the remaining species. Analyses requiring ortholog recovery in *S. splendens* are therefore depth‐limited. Reciprocal‐best‐hit ortholog identification recovered a mean of 4107 pairs for the four comparisons involving *S. splendens* (range 3485–5021), compared with 6336 for the six comparisons that do not (range 4571–7988), a reduction of approximately 35%. Pairwise contrasts involving *S. splendens* therefore screen a smaller fraction of the shared gene space and have correspondingly reduced power to detect elevated‐Ka/Ks candidates; the absence of a candidate locus in these comparisons should not be read as evidence of constraint. The combined nonredundant reference recovered 97.5% of BUSCO groups, but with 89.0% duplicated and only 8.5% single‐copy, reflecting retention of orthologous copies contributed by multiple species rather than their consolidation into consensus sequences. The generally high proportion of duplicated BUSCOs across all assemblies is consistent with transcriptome‐based assemblies retaining multiple isoforms and paralogous transcripts, rather than reflecting genome‐level duplication.

**FIGURE 1:**
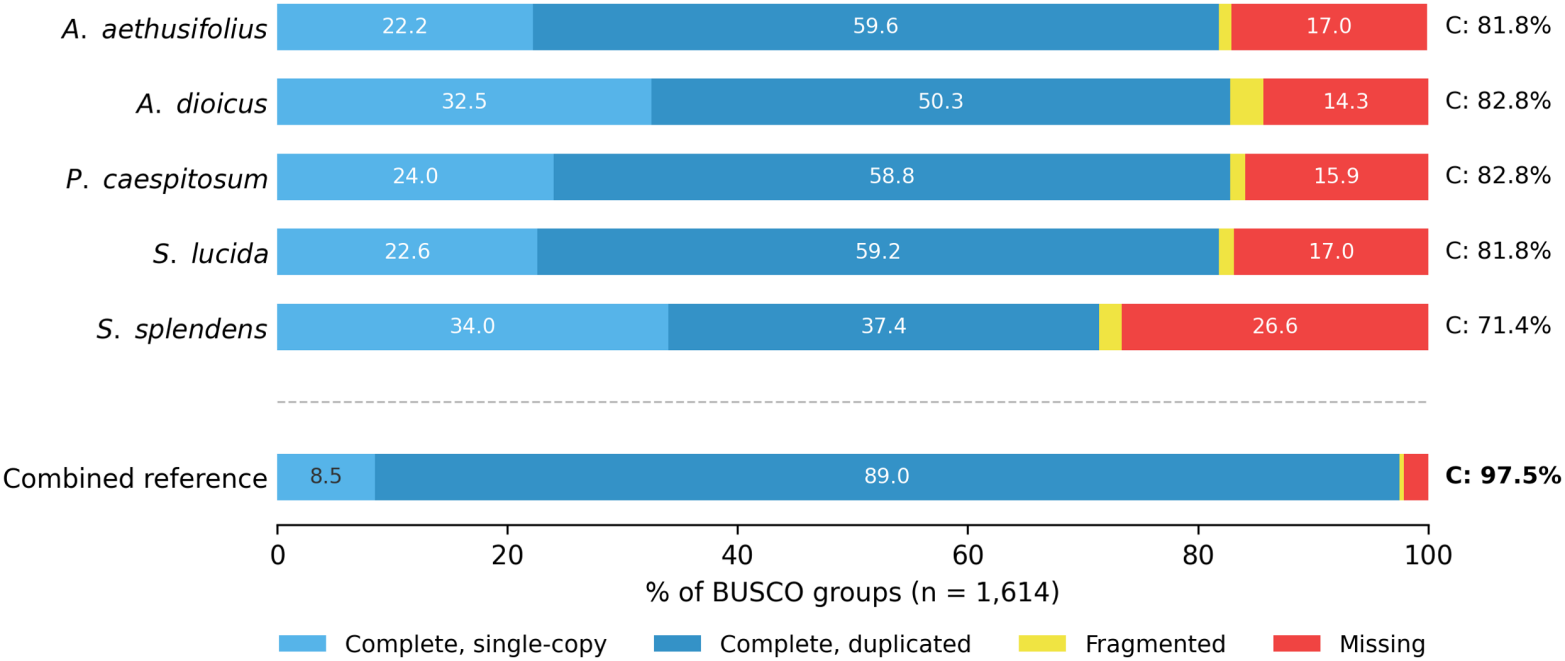
Benchmarking Universal Single‐Copy Orthologues (BUSCO) assessment of transcriptome completeness for the five species‐level Iso‐Seq assemblies and for the combined nonredundant reference, evaluated against the embryophyta_odb10 lineage (*n* = 1614 BUSCO groups). Bars show the percentage of BUSCO groups recovered as complete and single‐copy (S), complete and duplicated (D), fragmented (F), or missing (M); total completeness (C) is given at right.

The number of predicted CDSs was 32,981 for *A. aethusifolius*, 19,542 for *A. dioicus* , 14,006 for *S. splendens* , 27,411 for *S. lucida* , and 50,507 for *P. caespitosum* . Annotation across multiple public databases varied; however, *A. aethusifolius* and *A. dioicus* generally had higher annotation percentages (Figure 2). Specifically, *A. dioicus* had the most matches in NR (49%), NT (48%), and Swiss‐Prot (39%), whereas *A. aethusifolius* had the highest annotations in KO (37%), GO (40%), and KOG (43%). *P. caespitosum* had the lowest annotations among the species in all databases.

**FIGURE 2:**
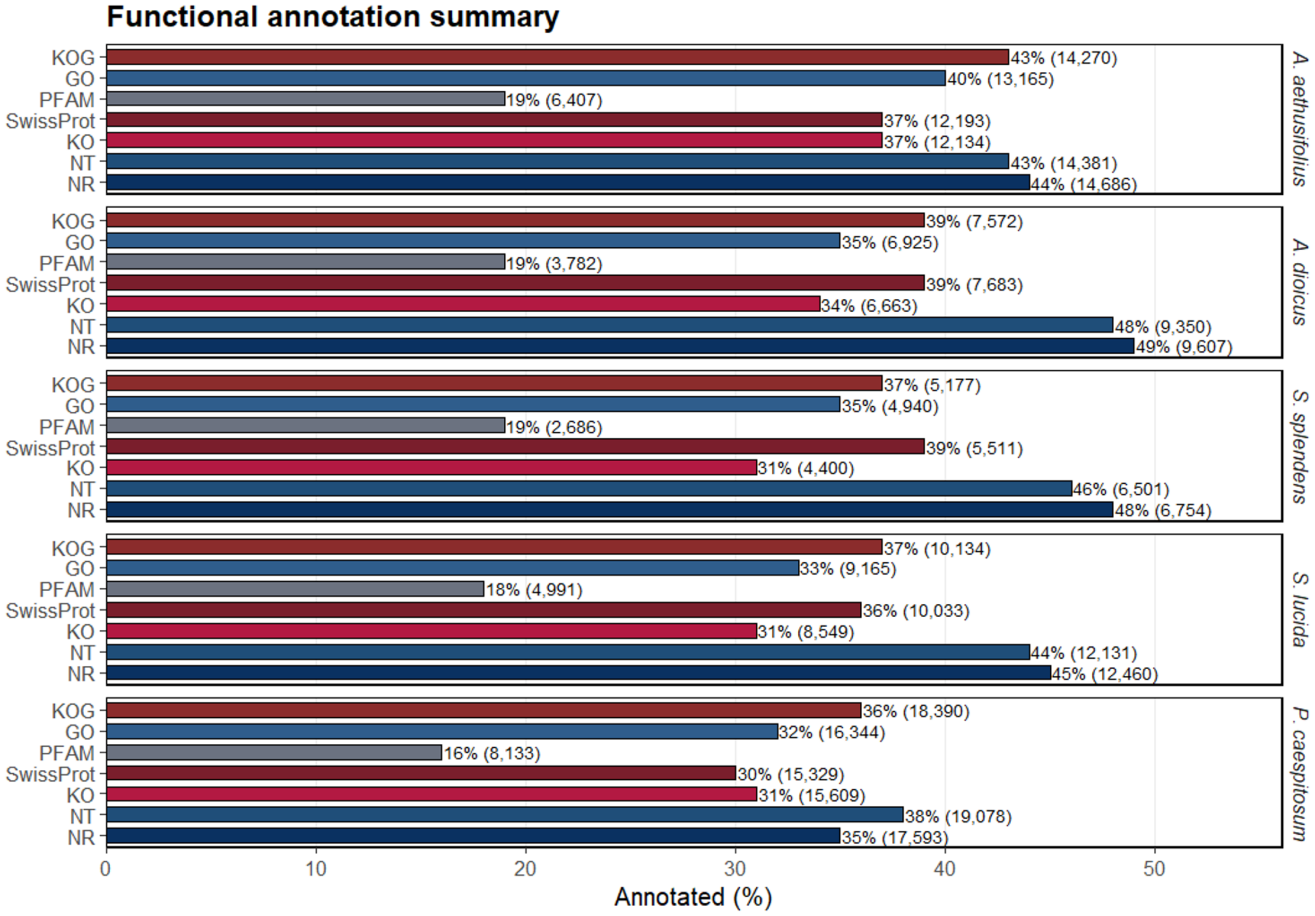
Annotation of the transcripts to public databases. GO, Gene Ontology; KO, KEGG orthology; KOG, clusters of orthologous groups of proteins; NR, NCBI nonredundant protein sequences; NT, NCBI nucleotide sequences; PFAM, protein family; SwissProt, a manually annotated and reviewed protein sequence database.

### 3.2 Hierarchical Clustering and PCA

To evaluate global similarities among samples, hierarchical clustering and PCA were performed using the top 500 most variable transcripts, selected based on variance across samples. Expression values were TMM‐normalized and row‐scaled (*Z*‐scores) prior to clustering. Sample clustering was conducted using Euclidean distance with hierarchical clustering (Figure 3). Both hierarchical clustering and PCA separated samples into three major groups corresponding to the genera *Aruncus*, *Spiraea*, and *Petrophytum*, consistent with taxonomy and previously reported phylogenetic relationships within Spiraeeae (Potter et al. 2007). In the PCA, the first two principal components explained 54.94% and 33.75% of the total variance, respectively, and biological replicates clustered tightly by species (Figure 4).

**FIGURE 3:**
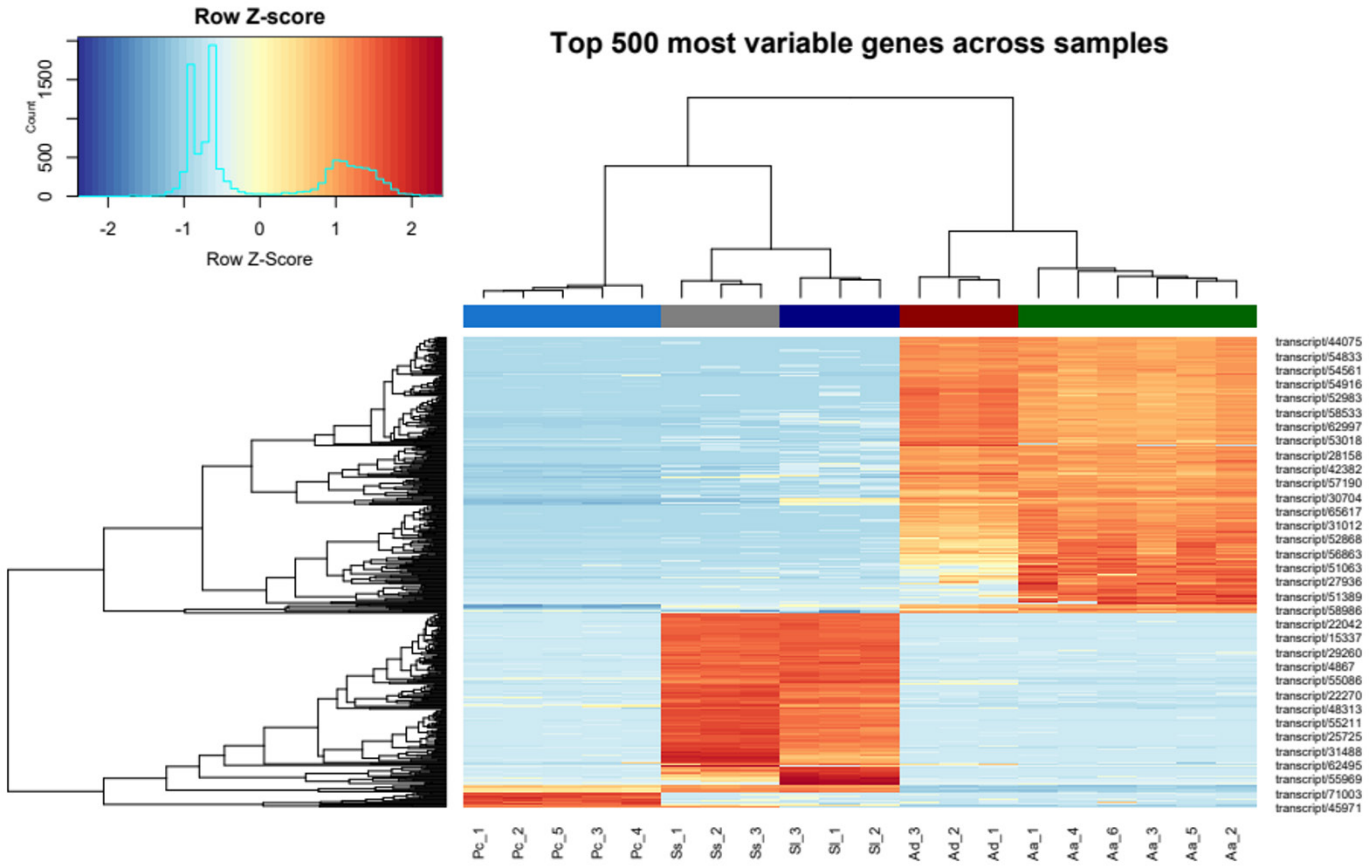
Heatmap of the top 500 most variable transcripts across five species ( *S. lucida* [Sl], *S. splendens* [Ss], *A. aethusifolius* [Aa], *A. dioicus* [Ad], and *P. caespitosum* [Pc]). Expression values are shown as row *Z*‐scores of TMM‐normalized expressions, with hierarchical clustering based on Euclidean distance. Samples cluster by species, illustrating divergence in transcript expression profiles among species.

**FIGURE 4:**
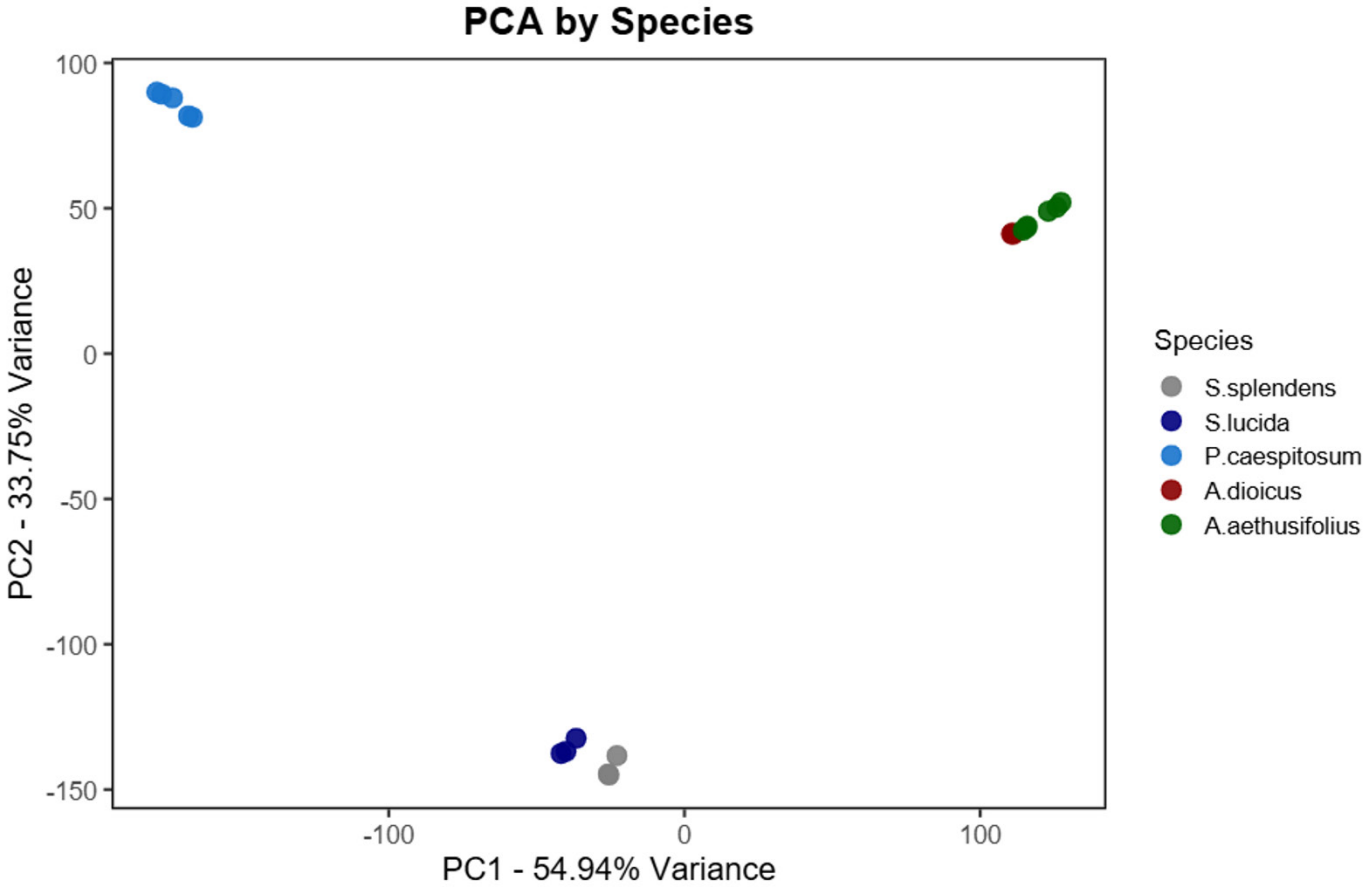
Principal component analysis (PCA) of TMM‐normalized expression values for the top 500 most variable transcripts across five species. Each point represents an individual biological replicate. The first two principal components explain 54.94% (PC1) and 33.75% (PC2) of the total variance, respectively, and biological replicates cluster by genus within Spiraeeae.

### 3.3 DETs and Functional Enrichment Analysis

Transcript abundance differed extensively among the five species. Transcript‐level diversity reflects differences in transcript abundance across species and provides a basis for comparative expression analyses. DETs were identified from Salmon‐estimated read counts after filtering and TMM normalization in edgeR, followed by voom transformation and linear modeling with limma. Pairwise comparisons among the five species yielded 10 contrasts, applying thresholds of |log_2_ fold change| ≥ 2 and FDR < 0.01.

Overall differential expression magnitude and direction across all pairwise contrasts are summarized in Figure S1. This figure provides the total numbers of upregulated and downregulated DETs for each comparison. Guided by the taxonomic structure recovered from hierarchical clustering and PCA, we focus here on three intergeneric comparisons; volcano plots for all remaining contrasts are provided in Figures S2–S8. In the comparison between *A. aethusifolius* and *P. caespitosum* , the volcano plot (Figure 5a) revealed extensive transcriptional divergence, with 6836 transcripts significantly upregulated and 4442 transcripts downregulated. Similarly, the comparison between *A. aethusifolius* and *S. splendens* (Figure 5b) identified 5350 upregulated and 4135 downregulated transcripts, indicating substantial expression divergence. In contrast, the comparison between *S. lucida* and *P. caespitosum* (Figure 5c) yielded 3811 upregulated and 2755 downregulated transcripts. Functional annotations of top‐ranked transcripts identified in each contrast are provided in Table S3. As expected, comparisons between more closely related species, such as *A. aethusifolius* vs. *A. dioicus* and *S. lucida* vs. *S. splendens* , resulted in substantially fewer DETs.

**FIGURE 5:**
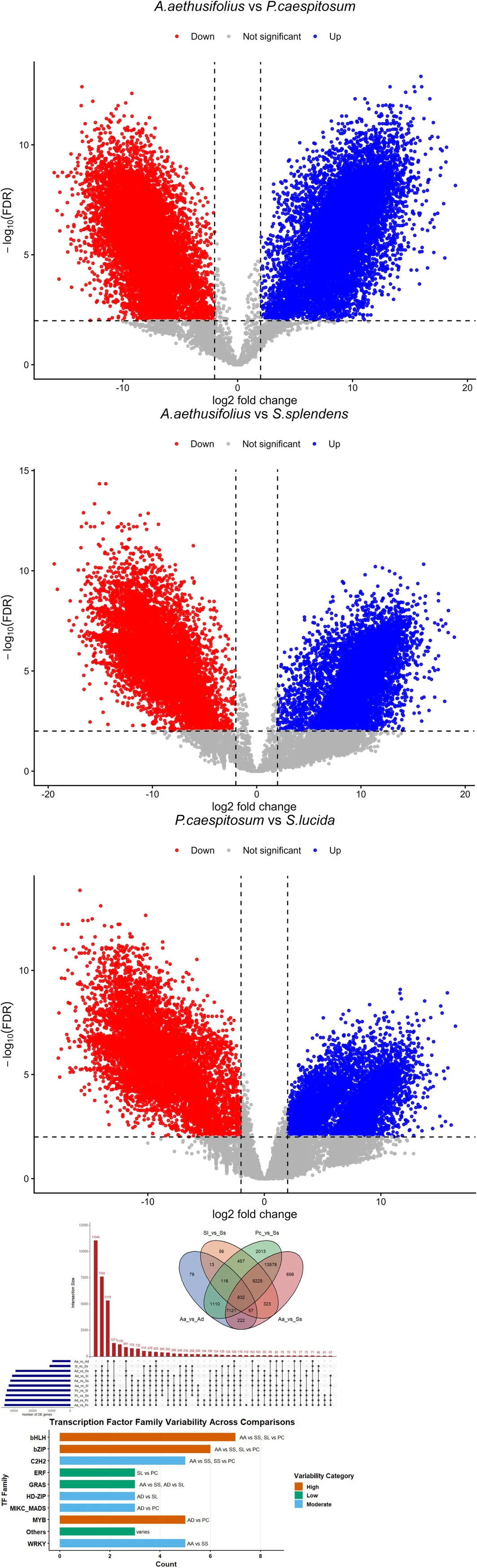
Differential expression and transcription factor variability across species comparisons. Volcano plots illustrate differentially expressed transcripts (DETs) in comparisons between (a) *A. aethusifolius* vs. *P. caespitosum* , (b) *A. aethusifolius* vs. *S. splendens* , and (c) *S. lucida* vs. *P. caespitosum* . Red points represent significantly upregulated transcripts, blue points represent significantly downregulated transcripts, and gray points indicate transcripts with no significant change. The Venn diagram and accompanying UpSet plot summarize shared and unique DETs across four focal comparisons and all pairwise comparisons accordingly (d). The bar chart highlights variability in transcription factor family expression across focal contrasts (e).

The overlap among DET sets across focal contrasts is summarized using a Venn diagram and an accompanying UpSet plot (Figure 5d). Several large, shared intersections were observed, indicating common transcriptional responses across multiple species comparisons, while each contrast also retained a distinct set of DETs, reflecting lineage‐specific expression differences. Total DET counts for each comparison are shown alongside the UpSet plot, which resolves complex intersections that the Venn diagram alone cannot capture.

Analysis of transcription factor (TF) families among DETs revealed pronounced differences across species contrasts (Figure 5e). TF family membership was assigned using PlantTFDB 5.0, and the plot summarizes the number of DE TF transcripts per family across comparisons. The bHLH and bZIP families showed the greatest variability in DE counts, particularly in contrasts involving *A. aethusifolius* vs. *S. splendens* and *S. lucida* vs. *P. caespitosum* . Moderate variability was observed for WRKY and C2H2 families, whereas GRAS and ERF families showed relatively stable patterns across most contrasts. This analysis describes TF family representation among DE transcripts and does not test TF binding‐site or motif enrichment.

### 3.4 Functional Enrichment of DETs

Functional annotation of DETs was performed based on Swiss‐Prot assignments, followed by KEGG pathway and Gene Ontology (GO) enrichment analyses. KEGG pathway enrichment revealed several taxon‐specific patterns (Figure 6a). Pathways related to aminoacyl‐tRNA biosynthesis, fatty acid metabolism, and peroxisome function were enriched exclusively in comparisons involving *A. aethusifolius* vs. *P. caespitosum* and *A. aethusifolius* vs. *S. splendens* . In contrast, ribosome and oxidative phosphorylation pathways were enriched only in comparisons involving *A. dioicus* against *S. lucida* , *S. splendens* , and *P. caespitosum* . GO enrichment analysis for molecular function (Figure 6b) showed broadly conserved patterns across comparisons, dominated by binding and catalytic activity terms, including ion binding, metal ion binding, and ATP binding. Nevertheless, certain functions displayed comparison‐specific enrichment, such as RNA binding in *A. aethusifolius* vs. *S. splendens* .

**FIGURE 6:**
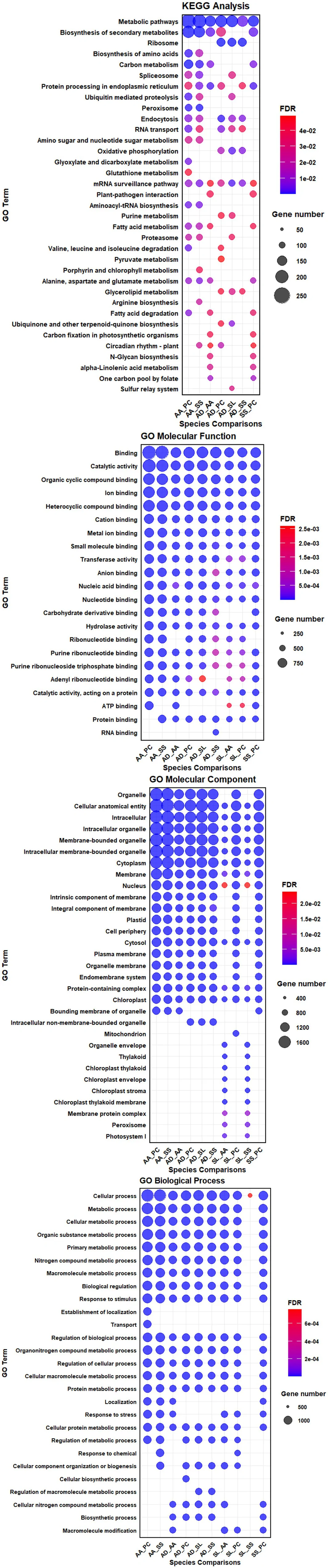
Functional enrichment analysis of upregulated differentially expressed transcripts (DETs). KEGG pathway enrichment analysis (a) was performed based on gene annotations associated with upregulated DETs. Gene Ontology (GO) enrichment was categorized into three main domains: molecular function (b), cellular component (c), and biological process (d). Bubble size represents the number of annotated genes associated with each term, whereas bubble color indicates the corresponding FDR value, as shown in the legend.

Enrichment analysis for cellular components (Figure 6c) highlighted widespread involvement of intracellular organelles, including membrane‐bounded organelles. Mitochondrial terms were uniquely enriched in the *S. lucida* vs. *P. caespitosum* comparison, whereas several chloroplast‐related terms were enriched only in comparisons involving *S. lucida* against *A. aethusifolius* and *S. splendens* . For biological process terms (Figure 6d), enrichment was dominated by metabolic processes across most contrasts, whereas “establishment of localization” and “transport” were enriched exclusively in the *A. aethusifolius* vs. *P. caespitosum* comparison.

### 3.5 WGCNA

WGCNA recovered modules of coexpressed transcripts, several of which correlated with species identity (Figure S11). Because between‐species variation dominates this dataset, modules are expected to track species largely as a restatement of that structure; we therefore treat these correlations as a descriptive summary of coexpression structure only. The full analysis, module assignments, module–trait correlations, and representative network visualizations are provided as Figures S9–S15.

### 3.6 Relative Sequence Divergence and Identification of Candidate Loci With Elevated Ka/Ks Ratios

We analyzed the distribution of synonymous substitutions per site (Ks) across orthologous gene pairs to summarize relative sequence divergence among species (Figure 7). The distributions resolve into three tiers corresponding to the subclade structure of the tribe. The two congeneric comparisons show the lowest divergence, with *A. aethusifolius* vs. *A. dioicus* exhibiting the sharpest peak (median Ks = 0.032; 75.2% of pairs below Ks = 0.1) and *S. splendens* vs. *S. lucida* next (median Ks = 0.066; 64.4% below 0.1). Intermediate distributions were recovered for the two intergeneric comparisons within the Spiraea–Petrophytum subclade ( *P. caespitosum* vs. *S. lucida* , median Ks = 0.140; *S. splendens* vs. *P. caespitosum* , median Ks = 0.142). The six comparisons between *Aruncus* and the remaining genera, which span the two subclades recognized by Potter et al. (2007), show the greatest divergence, with median Ks values of 0.226–0.260 and correspondingly broader distributions extending toward higher values. This ordering is concordant with the phylogenetic relationships within the tribe and provides an internal check on the ortholog assignments.

**FIGURE 7:**
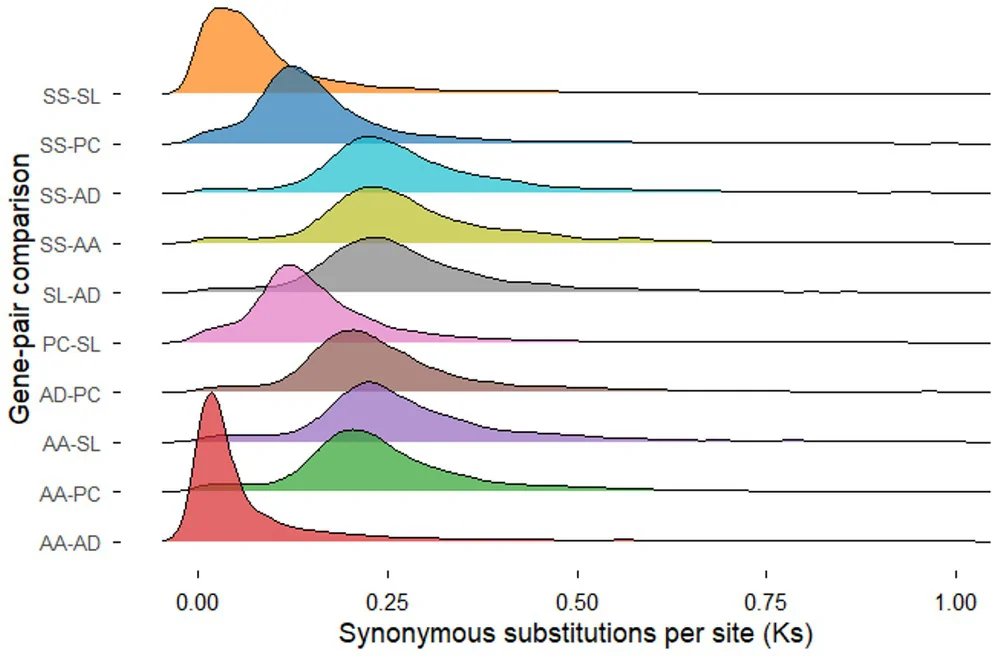
Distribution of synonymous substitution rates (Ks) for orthologous gene pairs across 10 pairwise species comparisons among five Spiraeeae species. Density curves illustrate variation in Ks distributions, consistent with differences in relative sequence divergence and substitution‐rate heterogeneity among lineages. Pairwise comparisons are denoted using species abbreviations as follows: AA, *A. aethusifolius*; AD, *A. dioicus* ; PC, *P. caespitosum* ; SL, *S. lucida* ; SS, *S. splendens* . For example, SS–SL indicates the comparison between *S. splendens* and *S. lucida* , and AA–PC indicates the comparison between *A. aethusifolius* and *P. caespitosum* . Comparisons with peaks at lower Ks values are consistent with lower relative sequence divergence, whereas broader distributions extending toward higher Ks values are consistent with greater relative sequence divergence.

To assess patterns of coding‐sequence divergence in protein‐coding genes, we calculated nonsynonymous substitution rates (Ka) and Ka/Ks ratios for orthologous gene pairs across 10 species comparisons. Four representative comparisons are shown in Figure 8, with the remaining analyses provided in Figure S16. We flagged ortholog pairs with Ka/Ks > 1 as candidate loci with elevated Ka/Ks ratios under the screening criteria used here. To reduce noise from unstable estimates associated with very recent divergence, ortholog pairs with Ks < 0.05 were excluded from downstream interpretation and are shown separately in the scatter plots.

**FIGURE 8:**
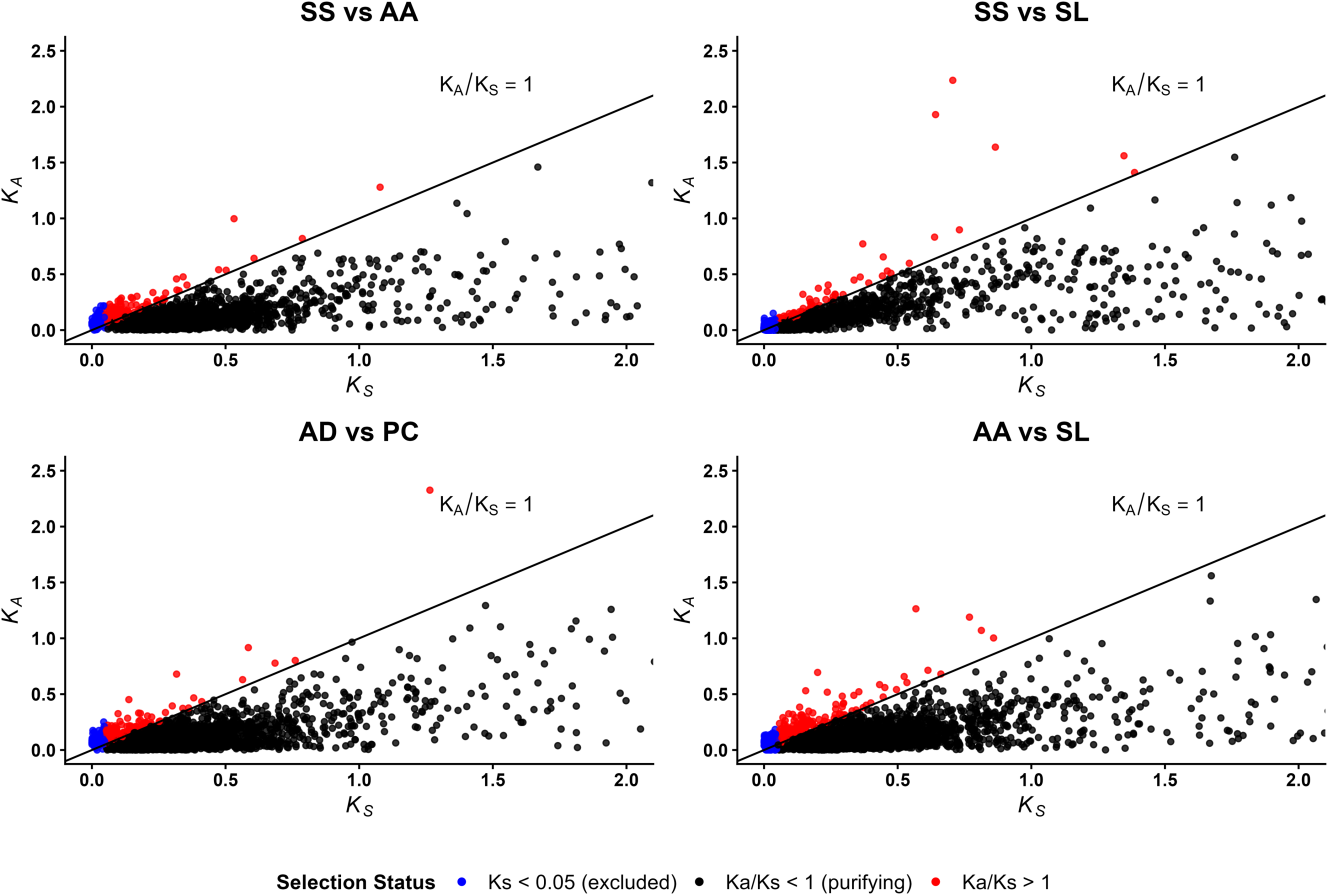
Comparative analysis of nonsynonymous (Ka) and synonymous (Ks) substitution rates for representative species pairs (AA, *Aruncus aethusifolius*; AD, *Aruncus dioicus*; PC, *Petrophytum caespitosum* ; SL, *Spiraea lucida* ; SS, *Spiraea splendens* ). Pairwise comparisons shown include SS vs. AA, SS vs. SL, AD vs. PC, and AA vs. SL. Each scatter plot shows the nonsynonymous substitution rate (Ka) plotted against the synonymous substitution rate (Ks) for orthologous gene pairs. The diagonal line indicates Ka = Ks (Ka/Ks = 1), corresponding to neutral expectations. Genes with Ka/Ks > 1 were flagged as candidate loci with elevated Ka/Ks ratios under our screening criteria (red points). Blue points represent ortholog pairs with very low Ks values (Ks < 0.05), which were excluded from inference due to unstable rate estimates but are shown for completeness.

Across all 10 comparisons, many orthologous gene pairs exhibited Ka/Ks ratios well below 1, indicating pervasive purifying selection on protein‐coding sequences, the dominant and most robust signal in these data. A small fraction of the retained pairs showed Ka/Ks > 1, ranging from 2.70% ( *A. dioicus* vs. *P. caespitosum* ) to 4.97% (*A. aethusifolius* vs. *A. dioicus* ) (Table S6). These elevated‐ratio pairs were concentrated at low synonymous divergence (Figure 8), where Ka/Ks estimates have high variance and are prone to inflation, so a substantial portion of this tail is expected from estimation error alone. Because a gene‐wide pairwise Ka/Ks ratio averages over all codons, it also cannot, by itself, distinguish a few positively selected sites from statistical noise. We therefore interpret these loci as screening‐level candidates for rapid sequence divergence; confirming any individual candidate would require formal codon‐based selection tests (e.g., site or branch‐site models).

The proportion of RBH ortholog pairs entering the candidate screen varied substantially among comparisons (Table S6). Retention after Ks filtering ranged from 37.9% (*A. aethusifolius* vs. *A. dioicus* ) to 96.1% ( *S. lucida* vs. *A. dioicus* ) and was inversely related to divergence: the two congeneric comparisons lost most pairs to the Ks < 0.05 exclusion, whereas the across‐subclade comparisons retained more than 95%. The candidate screen is therefore weighted toward the more divergent comparisons, and the small number of elevated‐Ka/Ks loci recovered within genera reflects the limited number of evaluable pairs as much as stronger constraint.

Functional annotation of candidate loci with elevated Ka/Ks ratios was based on Swiss‐Prot and Pfam annotations generated by Trinotate, and gene functions were summarized using curated database descriptions. The annotations of these screening candidates (which include an expected false positive fraction) spanned diverse functional categories across comparisons and are summarized descriptively, without inference of lineage‐specific selection. In *S. splendens* vs. *A. aethusifolius*, candidate loci included heat shock proteins, ENTH/VHS family proteins, and metal ion transporters. In *P. caespitosum* vs. *S. lucida* , candidate loci included zinc finger (RNA‐binding) proteins, nucleotidyltransferase family proteins, and cyclase‐associated protein 1. Comparisons between *S. splendens* and *P. caespitosum* identified candidate loci involved in seed storage, lipid transfer, vesicular transport, and oxygen‐dependent metabolic reactions. In *A. aethusifolius* vs. *P. caespitosum* , candidate loci were enriched for genes associated with ion transport, metal detoxification, genome maintenance, and specialized enzymatic functions, including cytochrome P450 family members. Additional candidate loci detected across other comparisons included uncoupling protein 5, histone deacetylase 3, SPRY‐domain proteins, kinase superfamily members, and calcineurin‐like metallophosphoesterases. Additional annotation details for candidate loci in the remaining contrasts are available in Table S4.

### 3.7 Leaf‐Developmental Regulators: Annotation and Sequence Conservation

To assess whether the genes that pattern leaf development are recoverable in these species, we annotated the combined nonredundant reference transcriptome directly (translated‐nucleotide search against UniProt/Swiss‐Prot, *E* < 1 × 10^−5^; gene‐level assignments for 40,609 transcripts, 78% of the quantified set) and screened the annotation for core leaf‐developmental regulators. The screen recovered members of the principal families that pattern leaf shape, size, and polarity: CIN‐type TCP transcription factors (leaf curvature and marginal growth), GROWTH‐REGULATING FACTORs with their GIF/AN3 coactivators (leaf size), YABBY and HD‐ZIP III/REVOLUTA genes (adaxial–abaxial polarity), and BELL and WOX homeobox genes (Table S5). REVOLUTA was among the most confidently annotated (≈85% amino acid identity).

We deliberately do not interpret the expression of these regulators. Because all five species were quantified against a single combined de novo reference, in which each species' reads map preferentially to its own assembled transcripts, transcript‐level abundance is not directly comparable across species for individual loci; apparent expression differences for any single gene cannot be separated from differences in transcript representation among assemblies. We therefore treat the screen as an annotation resource, a catalog of leaf‐developmental loci present in these transcriptomes, carrying no implication of differential developmental regulation.

Two further features bound the interpretation. First, the class I KNOX program (STM/BREVIPEDICELLUS), which promotes leaflet initiation in compound‐leaved species, was essentially unrepresented (a single transcript); the compound leaves of *Aruncus* are thus not accompanied by a detectable class I KNOX signature in these data, and we do not invoke this program. Second, many assignments are family‐level rather than gene‐specific; several CIN‐TCP transcripts, for example, match TCP4 at ~48% identity and are more accurately described as CIN‐TCP‐like.

At the sequence level, the developmental‐family orthologs that could be reliably tested and confidently annotated (best hit identity ≥ 55%) were uniformly under purifying selection (Figure 9; Ka/Ks ≈ 0.10–0.25), consistent with the genome‐wide pattern (Figure 7) and with the strong functional constraint expected for these conserved regulators. The conservation of their protein‐coding sequences indicates that the marked differences in leaf form among these species are unlikely to arise from amino acid changes in the regulators themselves, pointing instead toward regulatory or expression‐level evolution as the more probable basis, a hypothesis that can be tested directly with quantitative leaf morphometrics and depth‐matched, common garden expression profiling of comparable tissues and developmental stages.

**FIGURE 9:**
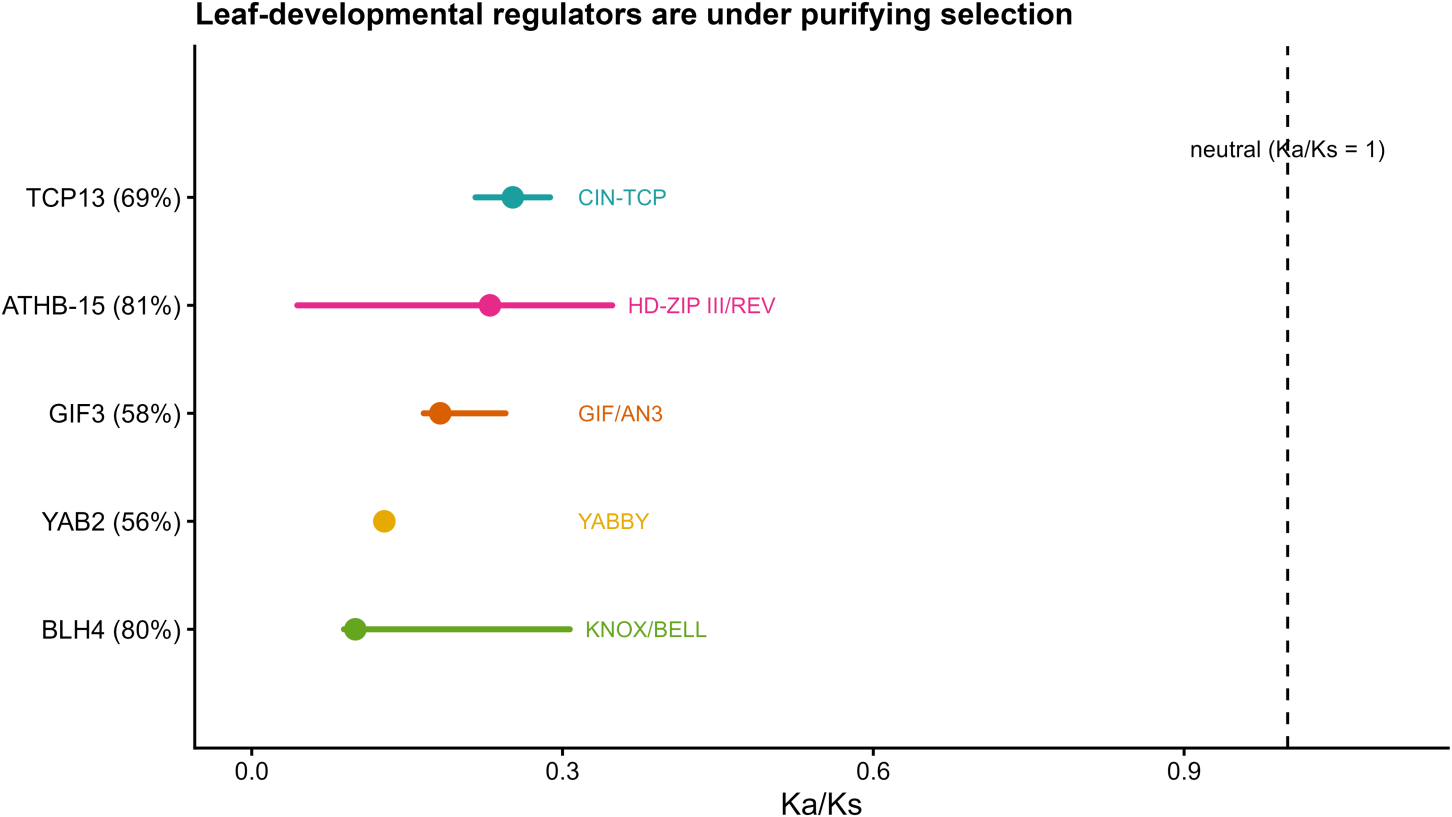
Leaf‐developmental regulators are under purifying selection. Ka/Ks for orthologs of core leaf‐developmental transcription‐factor families across the five species (one representative per species; cross‐species pairs), restricted to high‐confidence annotations (best hit identity ≥ 55%) and shown as the per‐family median (point) and range (line). Percent identities (in parentheses) indicate annotation confidence to the named best hit gene. The neutral expectation (Ka/Ks = 1) is shown for reference; all families fall well below it, indicating purifying selection on these regulators.

## 4 Discussion

Across the five species, the dominant axes of transcriptome variation corresponded to genus: clustering and PCA on the top 500 most variable transcripts separated samples into *Aruncus*, *Spiraea*, and *Petrophytum* groups, with replicates of each species clustering together (Figures 3 and 4). Notably, *S. lucida* and *S. splendens* grouped together despite being sampled under different conditions (potted vs. field), indicating that growth condition was not the principal driver of this structure. We nonetheless cannot separate species identity from sampling condition, so we describe the observed patterns of differential expression as species‐associated, without attributing them to genetically based regulatory divergence. The species‐ and genus‐structured pattern observed here is consistent with comparative transcriptomic studies in other plant systems where species identity and phylogenetic relationships dominate expression structure even across contrasting habitats, including altitudinally partitioned *Senecio* species (Chapman et al. 2013), ecologically divergent *Begonia* species with light‐niche differentiation (Emelianova and Kidner 2022), and habitat‐associated divergence in *Melastoma* species and their hybrid (Ng et al. 2019). Similarly, comparative transcriptomics has been used to identify lineage‐specific functional enrichments associated with environmental contrast in stressful habitats, such as intertidal mangroves (Guo et al. 2017), and to quantify regulatory divergence in life history traits such as the vernalization response of *Mimulus guttatus* (Preston et al. 2016).

The use of long‐read Iso‐Seq sequencing enables high‐confidence reconstruction of full‐length transcripts and the identification of alternative isoforms that are frequently unresolved by short‐read assemblies (Su et al. 2024). Isoform‐level diversity has been shown to capture biologically meaningful variation in plants, including *Zea mays* (Wang et al. 2016) and *Helianthus* (Badouin et al. 2017). While not optimized for absolute quantification, Iso‐Seq provides critical resolution of transcript structure and isoform diversity (Monzó et al. 2025).

Here, BUSCO completeness and annotation rates varied among species, with *P. caespitosum* showing high completeness but relatively low annotation proportion (Figures 1 and 2). Variation in annotation success has been reported in comparative transcriptome studies of nonmodel plants, including tropical tree species (Brousseau et al. 2014) and high‐alpine herbs (Jia et al. 2017), reflecting evolutionary distance from well‐represented reference taxa and incomplete database coverage.

Because replication varied among species (3–6 individuals), power is not uniform across contrasts, and we interpret borderline effects cautiously while emphasizing DETs supported by both stringent FDR control and large fold changes. In addition, because transcript abundance was estimated across divergent species using a combined nonredundant reference, some apparent differences in transcript abundance may also reflect differential mappability, transcript representation, or isoform composition across taxa, particularly in cross‐genus comparisons.

Differential expression analyses indicated that more distantly related species pairs generally exhibited greater transcriptomic divergence, consistent with phylogenetic distance contributing to regulatory differentiation (Figure 5; Figure S1). Specifically, cross‐genus contrasts yielded substantially more DETs than within‐genus contrasts (e.g., *A. aethusifolius* vs. *A. dioicus* and *S. lucida* vs. *S. splendens* ), mirroring the genus‐level structure observed in PCA and clustering (Figures 3 and 4). This pattern is consistent with transcriptomic divergence increasing with phylogenetic separation in our dataset, although technical effects related to cross‐species transcript representation may also contribute to the magnitude of some contrasts. In woody plants, comparative transcriptome analyses have identified both conserved and species‐specific expression programs across divergent lineages such as *Populus*, indicating that divergence can be expressed as shifts in tissue‐preferential regulation and pathway deployment (Hefer et al. 2015). Similarly, comparative expression profiling in *Eucalyptus* species/ecotypes from contrasting climates has shown distinct pathway‐level responses aligned with ecological context (Spokevicius et al. 2017). Lineage‐specific expression modules associated with divergent physiology/ecology are also evident in congeneric plant comparisons (Pavey et al. 2010).

Our observations of functional enrichment of unique GO and KEGG terms in specific contrasts suggest that differences in metabolic and localization processes may be associated with phenotypic differentiation between taxa. Across contrasts, the recurrence of organelle‐ and redox/energy‐associated categories (e.g., chloroplast/mitochondrial terms and oxidative phosphorylation) alongside lipid/fatty acid and peroxisome pathways (Figure 6) suggests that lineage differences in cellular energy balance and stress‐associated metabolic processes are a recurring component of transcriptomic differentiation among these taxa, although these enrichments do not by themselves establish adaptive or environmentally induced mechanisms. Enrichment of aminoacyl‐tRNA biosynthesis and fatty acid metabolism pathways in the *A. aethusifolius* vs. *P. caespitosum* contrast is consistent with evidence that nutrient limitation and nutrient‐efficiency differences can drive broad metabolic reprogramming at the transcriptome level (Prodhan et al. 2022; Woo et al. 2012; Wu et al. 2003). The organellar and energy‐related enrichments observed here (Figure 6) are consistent with well‐documented lineage differences in organellar transcriptomes and organelle‐associated regulatory processes across plants (Grewe et al. 2014; Oldenkott et al. 2014).

In our dataset, transcription factor families contributed disproportionately to expression divergence, with bHLH and bZIP showing the largest variability in differentially expressed TF transcript counts across contrasts (Figure 5e). Because TF families often sit at the interface of growth regulation and abiotic‐stress signaling, this TF‐family skew is consistent with a regulatory dimension of the lineage‐structured expression differences observed here; it is not direct evidence of stress adaptation per se.

Sequence‐level analyses of synonymous (Ks) and nonsynonymous (Ka) substitutions indicated that orthologous protein‐coding genes are predominantly under purifying selection, the clearest evolutionary signal in this study. A small fraction showed Ka/Ks > 1, but these ratios were concentrated at low Ks where estimates are unstable and represent gene‐wide pairwise averages that cannot by themselves demonstrate positive selection. The predominance of purifying selection with a tail of more rapidly diverging genes is widely observed across plant genomes, where functional constraint dominates but genes involved in stress response, transport, and regulatory processes often show comparatively higher nonsynonymous substitution rates (Ingvarsson 2010; Yang and Nielsen 2002). Consistent with our analyses showing that several candidate transcripts are annotated as stress‐ and transport‐related functions (Figure S16; Table S4), similar functional categories have been reported in wild rice relatives adapted to saline habitats (Padmavathi et al. 2023). These results do not establish adaptive causation, but they highlight candidate functional classes for future testing in a phylogenetic and ecological framework.

The taxa sampled here span contrasting habitats and leaf forms, but our design included neither habitat measurements nor controlled treatments, so we cannot definitively attribute observed expression differences to either ecology or leaf morphology. We instead use the data to nominate candidate processes. Leaf shape and size emerge from coordinated cell proliferation, expansion, and cell wall remodeling (Bortolami et al. 2024), and our screen recovered key regulators of these processes, CIN‐TCP, GRF/GIF, YABBY, and HD‐ZIP III/REVOLUTA (Figure 9), alongside transcripts annotated for cell wall modification and organellar function. These regulators are present and, at the sequence level, conserved under purifying selection, so although they are strong candidates for involvement in producing diversity of leaf forms in Spiraeeae, their contribution is more likely to act through regulatory rather than coding differences between lineages. The class I KNOX program associated with compound leaf development was essentially absent, so we explicitly do not propose a KNOX‐based mechanism for the compound leaves of *Aruncus*. Our observations identify a bounded set of regulators and pathways that future studies can test using quantitative leaf morphometrics and ecophysiological measurements, ideally in a common garden design.

The five species span a wide range of leaf architecture: simple, entire‐margined leaves in *P. caespitosum* , simple toothed leaves in *Spiraea*, and two to three times compound, toothed leaves in *Aruncus*. These are qualitative, taxonomically diagnostic differences. Against this morphological divergence, the canonical regulators of leaf shape, size, and polarity (CIN‐TCP, GRF/GIF, YABBY, HD‐ZIP III/REVOLUTA, and BELL/WOX homeobox genes) are recoverable in all five transcriptomes and are uniformly under strong purifying selection. Conservation of these CDSs across lineages with such divergent leaf forms is consistent with morphological differences arising through changes in the regulatory deployment of a conserved developmental toolkit rather than through changes in the encoded proteins.

A further constraint arises from the quantification strategy. Because expression was measured against a reference assembled from species‐specific Iso‐Seq contigs, reads mapped more efficiently to transcripts contributed by their own species (70.5%–85.1%) than to those contributed by others (25.1%–77.0%). Normalization corrects for differences in library size but not for differential mappability, so the magnitude of between‐species expression differences reported here is expected to be inflated relative to true biological divergence. Comparisons within a genus, where reference representation is more even, are correspondingly less affected than comparisons between genera.

Together, these results indicate that differences among closely related Spiraeeae lineages are reflected across multiple transcriptomic layers, including expression profiles, coexpression network structure, and protein‐CDS evolution. However, transcriptomic patterns alone cannot establish causal links between regulatory divergence and ecological differentiation. The convergence of expression, network, and sequence‐level signals instead highlights specific biological processes and gene families as candidates for further investigation. Integrating transcriptomic data with ecological measurements, physiological traits, and population‐level sampling will be essential for disentangling ecologically relevant regulatory variation from lineage‐specific constraint, as emphasized in comparative genomic studies of oaks, pines, and *Arabidopsis* relatives (Lowry et al. 2013; Plomion et al. 2018).

## 5 Conclusion

We present long‐read transcriptomes for five species of the tribe Spiraeeae and use them to describe transcriptome‐wide expression patterns, coding‐sequence divergence among orthologs, and the recovery and sequence conservation of key leaf‐developmental regulators. Expression differences among species are pervasive and group samples by species and genus; however, because species identity and growth conditions were not experimentally separated, we interpret them as species‐associated patterns and make no adaptive or mechanistic claims. The clearest evolutionary signal comes from orthologous CDSs, which are predominantly under purifying selection, with a small set of loci showing elevated Ka/Ks. Together, these data provide a comparative resource for a poorly characterized Rosaceae clade and a bounded set of candidate genes, pathways, and rapidly evolving loci to be tested with controlled developmental and ecological sampling.

## Author Contributions

**Daniel Potter** conceived and supervised the study; **Armin M. Mafakheri** performed the analyses and wrote the draft; **Armin M. Mafakheri** and **Daniel Potter** revised the manuscript.

## Funding

This work was supported by the National Institute of Food and Agriculture (CA‐D‐PLS‐6273‐H).

## Conflicts of Interest

The authors declare no conflicts of interest.

## Supporting information


**Figure S1:** Up‐ and downregulated differentially expressed transcripts (DETs) across pairwise species contrasts. Horizontal bars show the number of DETs that are significantly upregulated (red) or downregulated (blue) in the first species listed in each contrast relative to the second, based on limma–voom analysis of filtered, TMM‐normalized counts (|log2FC|≥ 2; FDR < 0.01). Species abbreviations: Aa, *Aruncus aethusifolius*; Ad, *Aruncus dioicus* ; Pc, *Petrophytum caespitosum* ; Sl, *Spiraea lucida* ; Ss, *Spiraea splendens* .
**Figure S2:** Volcano plot of differential transcript expression between *A. dioicus* and *A. aethusifolius*. Visualization parameters and significance thresholds are consistent with those described in the Methods.
**Figure S3:** Volcano plot of differential transcript expression between *A. aethusifolius* and *S. splendens* . Visualization parameters and significance thresholds are consistent with those described in the Methods.
**Figure S4:** Volcano plot of differential transcript expression between *A. dioicus* and *P. caespitosum* . Visualization parameters and significance thresholds are consistent with those described in the Methods.
**Figure S5:** Volcano plot of differential transcript expression between *A. dioicus* and *S. lucida* . Visualization parameters and significance thresholds are consistent with those described in the Methods.
**Figure S6:** Volcano plot of differential transcript expression between *A. dioicus* and *S. splendens* . Visualization parameters and significance thresholds are consistent with those described in the Methods.
**Figure S7:** Volcano plot of differential transcript expression between *S. splendens* and *P. caespitosum* . Visualization parameters and significance thresholds are consistent with those described in the Methods.
**Figure S8:** Volcano plot of differential transcript expression between *S. lucida* and *S. splendens* . Visualization parameters and significance thresholds are consistent with those described in the Methods.
**Figure S9:** Scale‐free topology fit and mean connectivity analysis for WGCNA soft‐threshold selection. Left panel shows the scale‐free topology model fit (signed *R* ^2^) across candidate soft‐thresholding powers, and the right panel shows the corresponding mean connectivity.
**Figure S10:** Weighted gene coexpression network analysis (WGCNA). Hierarchical clustering dendrogram of transcripts based on topological overlap, with module assignments shown below. Colors indicate gene modules identified using dynamic tree cutting and subsequent module merging based on eigengene similarity.
**Figure S11:** Heatmap of module–trait relationships showing correlations between module eigengenes and species identity. Values represent Pearson correlation coefficients, with corresponding *p*‐values shown in parentheses. Color intensity reflects the strength and direction of the correlation.
**Figure S12:** Cytoscape network visualization of the cyan WGCNA module. Nodes represent transcripts and edges represent coexpression relationships, visualized to highlight highly connected genes within the module.
**Figure S13:** Cytoscape network visualization of the dark gray WGCNA module. Nodes represent transcripts and edges represent coexpression relationships, visualized to highlight highly connected genes within the module.
**Figure S14:** Cytoscape network visualization of the green–yellow WGCNA module. Network structure illustrates coexpression relationships among transcripts identified within this module.
**Figure S15:** Cytoscape network visualization of the light green WGCNA module. Nodes and edges represent transcript‐level coexpression relationships inferred from the WGCNA adjacency matrix.
**Figure S16:** Comparative analysis of nonsynonymous (Ka) and synonymous (Ks) substitution rates for the remaining six species pairs. Each scatter plot shows the nonsynonymous substitution rate (Ka) plotted against the synonymous substitution rate (Ks) for orthologous gene pairs. The diagonal line indicates Ka = Ks (Ka/Ks = 1), corresponding to neutral expectations. Genes with Ka/Ks > 1 were flagged as candidate loci with elevated Ka/Ks ratios under our screening criteria (red points). Blue points represent ortholog pairs with very low Ks values (Ks < 0.05), which were excluded from inference due to unstable rate estimates but are shown for completeness.
**Table S1:** Characteristics of the genera of Rosaceae tribe Spiraeeae (from Potter et al. 2007).
**Table S2:** Taxa sampled for this study. Voucher specimens are all deposited in the herbarium of the UC Davis Center for Plant Diversity (DAV). Information from Lis (2014a, 2014b) and Potter and Rosatti (2012). Biological Replicates refers to number of individuals sampled.
**Table S3:** Functional annotation of top‐ranked genes identified in volcano plot analyses across species contrasts.
**Table S4:** Functional annotation summary of candidate loci with elevated Ka/Ks ratios.
**Table S5:** Leaf‐developmental regulator transcripts recovered in the combined reference, with best hit gene, family assignment, and percent identity.
**Table S6:** Ortholog pairs evaluated and retained after Ks filtering in each pairwise species comparison.

## Data Availability

Raw sequencing data for the five species have been deposited in the NCBI Sequence Read Archive (SRA) under BioProject accession number PRJNA1394637 and are publicly available. The BioProject comprises 25 runs distributed across 20 individual‐level BioSamples (SAMN62390016–SAMN62390035): 20 TagSeq libraries (Illumina HiSeq 4000, single‐end 93 bp), one per individual, which underlie all expression analyses reported here, and five Iso‐Seq FLNC read sets (PacBio Sequel II), one per species, generated from the representative individuals used to construct the reference transcriptomes (*A. aethusifolius* Aa7, SAMN62390019, SRR40183236; *A. dioicus* Ad7, SAMN62390022, SRR40183235; *P. caespitosum* P10, SAMN62390029, SRR40183234; *S. lucida* Sl7, SAMN62390030, SRR40183233; *S. splendens* Ss1, SAMN62390033, SRR40183232). The Iso‐Seq spot counts deposited in the SRA correspond exactly to the full‐length read counts (num_reads_fl) reported in Table 1. The five reference transcriptome assemblies have been deposited at DDBJ/ENA/GenBank under the same BioProject as Transcriptome Shotgun Assembly projects, under accessions GLQD00000000 (*A. aethusifolius* Aa7), GLQC00000000 ( *A. dioicus* Ad7), GLQE00000000 ( *P. caespitosum* P10), GLQF00000000 ( *S. lucida* Sl7), and GLQG00000000 ( *S. splendens* Ss1). The versions described in this paper are the first versions (GLQD01000000, GLQC01000000, GLQE01000000, GLQF01000000, and GLQG01000000). Supplementary figures and tables are included with the submission. Analysis scripts and processed expression data are available in a public GitHub repository: https://github.com/arminmm91/spiraeeae‐comparative‐transcriptomics.

## References

[pld370202-bib-0001] Badouin, H. , J. Gouzy , C. J. Grassa , et al. 2017. “The Sunflower Genome Provides Insights Into Oil Metabolism, Flowering and Asterid Evolution.” Nature 546: 148–152. 10.1038/nature22380 28538728

[pld370202-bib-0002] Blanco‐Ulate, B. , E. Vincenti , A. L. Powell , and D. Cantu . 2013. “Tomato Transcriptome and Mutant Analyses Suggest a Role for Plant Stress Hormones in the Interaction Between Fruit and *Botrytis cinerea* .” Frontiers in Plant Science 4: 142. 10.3389/fpls.2013.00142 PMC3653111 23717322

[pld370202-bib-0003] Bortolami, G. , T. A. de Werk , M. Larter , et al. 2024. “Integrating Gene Expression Analysis and Ecophysiological Responses to Water Deficit in Leaves of Tomato Plants.” Scientific Reports 14: 29024. 10.1038/s41598-024-80261-0 PMC11584733 39578554

[pld370202-bib-0004] Brousseau, L. , A. Tinaut , C. Duret , T. Lang , P. Garnier‐Gere , and I. Scotti . 2014. “High‐Throughput Transcriptome Sequencing and Preliminary Functional Analysis in Four Neotropical Tree Species.” BMC Genomics 15: 238. 10.1186/1471-2164-15-238 PMC3986928 24673733

[pld370202-bib-0005] Bryant, D. M. , K. Johnson , T. DiTommaso , et al. 2017. “A Tissue‐Mapped Axolotl De Novo Transcriptome Enables Identification of Limb Regeneration Factors.” Cell Reports 18: 762–776. 10.1016/j.celrep.2016.12.063 PMC5419050 28099853

[pld370202-bib-0006] Byrne, K. P. , and K. H. Wolfe . 2007. “Consistent Patterns of Rate Asymmetry and Gene Loss Indicate Widespread Neofunctionalization of Yeast Genes After Whole‐Genome Duplication.” Genetics 175: 1341–1350. 10.1534/genetics.106.066951 PMC1840088 17194778

[pld370202-bib-0007] Chang, E. , X. Liu , J. Chen , J. Sun , S. Yang , and J. Liu . 2023. “Comparative Transcriptomes Reveal the Disjunction Adaptive Strategy of *Thuja* Species in East Asia and North America.” Journal of Forest Research 34: 1963–1974.

[pld370202-bib-0008] Chapman, M. A. , S. J. Hiscock , and D. A. Filatov . 2013. “Genomic Divergence During Speciation Driven by Adaptation to Altitude.” Molecular Biology and Evolution 30: 2553–2567. 10.1093/molbev/mst168 PMC3840311 24077768

[pld370202-bib-0009] Conway, J. R. , A. Lex , and N. Gehlenborg . 2017. “UpSetR: An R Package for the Visualization of Intersecting Sets and Their Properties.” Bioinformatics 33: 2938–2940. 10.1093/bioinformatics/btx364 PMC5870712 28645171

[pld370202-bib-0010] Emelianova, K. , and C. Kidner . 2022. “Comparative Transcriptome Analysis of Two Closely Related Begonia Species Reveals Divergent Patterns in Key Light‐Regulated Pathways.” Edinburgh Journal of Botany 79: 1–18.

[pld370202-bib-0011] Fay, J. C. , and C. I. Wu . 2003. “Sequence Divergence, Functional Constraint, and Selection in Protein Evolution.” Annual Review of Genomics and Human Genetics 4: 213–235. 10.1146/annurev.genom.4.020303.162528 14527302

[pld370202-bib-0012] Fu, L. , B. Niu , Z. Zhu , S. Wu , and W. Li . 2012. “CD‐HIT: Accelerated for Clustering the Next‐Generation Sequencing Data.” Bioinformatics 28: 3150–3152. 10.1093/bioinformatics/bts565 PMC3516142 23060610

[pld370202-bib-0013] Gao, C.‐H. , G. Yu , and P. Cai . 2021. “ggVennDiagram: An Intuitive, Easy‐to‐Use, and Highly Customizable R Package to Generate Venn Diagram.” Frontiers in Genetics 12: 706907. 10.3389/fgene.2021.706907 PMC8452859 34557218

[pld370202-bib-0014] Ge, S. X. , D. Jung , and R. Yao . 2020. “ShinyGO: A Graphical Gene‐Set Enrichment Tool for Animals and Plants.” Bioinformatics 36: 2628–2629. 10.1093/bioinformatics/btz931 PMC7178415 31882993

[pld370202-bib-0015] Greenberg, A. J. , J. R. Moran , S. Fang , and C. I. Wu . 2006. “Adaptive Loss of an Old Duplicated Gene During Incipient Speciation.” Molecular Biology and Evolution 23: 401–410. 10.1093/molbev/msj045 16251509

[pld370202-bib-0016] Grewe, F. , P. P. Edger , I. Keren , et al. 2014. “Comparative Analysis of 11 Brassicales Mitochondrial Genomes and the Mitochondrial Transcriptome of *Brassica oleracea* .” Mitochondrion 19: 135–143. 10.1016/j.mito.2014.05.008 24907441

[pld370202-bib-0017] Gul, H. , Z. Tong , X. Han , et al. 2019. “Comparative Transcriptome Analysis Between Ornamental Apple Species Provides Insights Into Mechanism of Double Flowering.” Agronomy 9: 112.

[pld370202-bib-0018] Guo, W. , H. Wu , Z. Zhang , et al. 2017. “Comparative Analysis of Transcriptomes in Rhizophoraceae Provides Insights Into the Origin and Adaptive Evolution of Mangrove Plants in Intertidal Environments.” Frontiers in Plant Science 8: 795. 10.3389/fpls.2017.00795 PMC5432612 28559911

[pld370202-bib-0019] Haas, B. J. , A. Papanicolaou , M. Yassour , et al. 2013. “De Novo Transcript Sequence Reconstruction From RNA‐Seq Using the Trinity Platform for Reference Generation and Analysis.” Nature Protocols 8: 1494–1512. 10.1038/nprot.2013.084 PMC3875132 23845962

[pld370202-bib-0020] Hefer, C. A. , E. Mizrachi , A. A. Myburg , C. J. Douglas , and S. D. Mansfield . 2015. “Comparative Interrogation of the Developing Xylem Transcriptomes of Two Wood‐Forming Species: *Populus trichocarpa* and *Eucalyptus grandis* .” New Phytologist 206: 1391–1405. 10.1111/nph.13277 25659405

[pld370202-bib-0021] Hummer, K. E. , and J. Janick . 2009. “Rosaceae: Taxonomy, Economic Importance, Genomics.” In Genetics and Genomics of Rosaceae, edited by K. M. Folta and S. E. Gardiner , 1–17. Springer.

[pld370202-bib-0022] Illa, E. , D. J. Sargent , E. Lopez Girona , et al. 2011. “Comparative Analysis of Rosaceous Genomes and the Reconstruction of a Putative Ancestral Genome for the Family.” BMC Evolutionary Biology 11: 9. 10.1186/1471-2148-11-9 PMC3033827 21226921

[pld370202-bib-0023] Ingvarsson, P. K. 2010. “Natural Selection on Synonymous and Nonsynonymous Mutations Shapes Patterns of Polymorphism in *Populus tremula* .” Molecular Biology and Evolution 27: 650–660. 10.1093/molbev/msp255 19837657

[pld370202-bib-0024] Jia, Y. , M. L. Liu , M. Yue , Z. Zhao , G. F. Zhao , and Z. H. Li . 2017. “Comparative Transcriptome Analysis Reveals Adaptive Evolution of *Notopterygium incisum* and *Notopterygium franchetii*, Two High‐Alpine Herbal Species Endemic to China.” Molecules 22: 1158. 10.3390/molecules22071158 PMC6152189 28696392

[pld370202-bib-0025] Koenig, D. , J. M. Jimenez‐Gomez , S. Kimura , et al. 2013. “Comparative Transcriptomics Reveals Patterns of Selection in Domesticated and Wild Tomato.” Proceedings of the National Academy of Sciences of the United States of America 110: E2655–E2662. 10.1073/pnas.1309606110 PMC3710864 23803858

[pld370202-bib-0026] Langfelder, P. , and S. Horvath . 2008. “WGCNA: An R Package for Weighted Correlation Network Analysis.” BMC Bioinformatics 9: 559. 10.1186/1471-2105-9-559 PMC2631488 19114008

[pld370202-bib-0063] Lis, R. A. 2014a. “Petrophytum.” In Flora of North America North of Mexico, Vol. 9: Magnoliophyta: Picramniaceae to Rosaceae, edited by Flora of North America Editorial Committee , 411–413. Oxford University Press.

[pld370202-bib-0064] Lis, R. A. 2014b. “Spiraea.” In Flora of North America North of Mexico, Vol. 9: Magnoliophyta: Picramniaceae to Rosaceae, edited by Flora of North America Editorial Committee , 398–409. Oxford University Press.

[pld370202-bib-0028] Lowry, D. B. , T. L. Logan , L. Santuari , et al. 2013. “Expression Quantitative Trait Locus Mapping Across Water Availability Environments Reveals Contrasting Associations With Genomic Features in *Arabidopsis* .” Plant Cell 25: 3266–3279. 10.1105/tpc.113.115352 PMC3809531 24045022

[pld370202-bib-0029] Manni, M. , M. R. Berkeley , M. Seppey , F. A. Simao , and E. M. Zdobnov . 2021. “BUSCO Update: Novel and Streamlined Workflows Along With Broader and Deeper Phylogenetic Coverage for Scoring of Eukaryotic, Prokaryotic, and Viral Genomes.” Molecular Biology and Evolution 38: 4647–4654. 10.1093/molbev/msab199 PMC8476166 34320186

[pld370202-bib-0030] Monzó, C. , T. Liu , and A. Conesa . 2025. “Transcriptomics in the Era of Long‐Read Sequencing.” Nature Reviews Genetics 26: 681–701. 10.1038/s41576-025-00828-z 40155769

[pld370202-bib-0067] Moreno‐Hagelsieb, G. , and K. Latimer . 2008. “Choosing BLAST Options for Better Detection of Orthologs as Reciprocal Best Hits.” Bioinformatics 24: 319–324. 10.1093/bioinformatics/btm585 18042555

[pld370202-bib-0066] Nei, M. , and T. Gojobori . 1986. “Simple Methods for Estimating the Numbers of Synonymous and Nonsynonymous Nucleotide Substitutions.” Molecular Biology and Evolution 3: 418–426. 10.1093/oxfordjournals.molbev.a040410 3444411

[pld370202-bib-0031] Ng, W. L. , W. Wu , P. Zou , and R. Zhou . 2019. “Comparative Transcriptomics Sheds Light on Differential Adaptation and Species Diversification Between Two *Melastoma* Species and Their F1 Hybrid.” AoB Plants 11: plz019. 10.1093/aobpla/plz019 PMC6481908 31037213

[pld370202-bib-0032] Oldenkott, B. , K. Yamaguchi , S. Tsuji‐Tsukinoki , N. Knie , and V. Knoop . 2014. “Chloroplast RNA Editing Going Extreme: More Than 3400 Events of C‐to‐U Editing in the Chloroplast Transcriptome of the Lycophyte *Selaginella uncinata* .” RNA 20: 1499–1506. 10.1261/rna.045575.114 PMC4174432 25142065

[pld370202-bib-0033] Padmavathi, G. , U. Bangale , K. N. Rao , et al. 2023. “Progress and Prospects in Harnessing Wild Relatives for Genetic Enhancement of Salt Tolerance in Rice.” Frontiers in Plant Science 14: 1253726. 10.3389/fpls.2023.1253726 PMC10870985 38371332

[pld370202-bib-0034] Pavey, S. A. , H. Collin , P. Nosil , and S. M. Rogers . 2010. “The Role of Gene Expression in Ecological Speciation.” Annals of the New York Academy of Sciences 1206: 110–129. 10.1111/j.1749-6632.2010.05765.x PMC3066407 20860685

[pld370202-bib-0035] Plomion, C. , J. M. Aury J. Amselem , et al. 2018. “Oak Genome Reveals Facets of Long Lifespan.” Nature Plants 4: 440–452. 10.1038/s41477-018-0172-3 PMC6086335 29915331

[pld370202-bib-0068] Potter, D. , and T. J. Rosatti . 2012. “Spiraea.” In The Jepson Manual: Vascular Plants of California, edited by B. G. Baldwin , D. H. Goldman , D. J. Keil , R. Patterson , T. J. Rosatti , and D. H. Wilken , 2nd ed., 1205. University of California Press.

[pld370202-bib-0036] Potter, D. , S. M. Still , T. Grebenc , et al. 2007. “Phylogenetic Relationships in Tribe *Spiraeeae* (Rosaceae) Inferred From Nucleotide Sequence Data.” Plant Systematics and Evolution 266: 105–118.

[pld370202-bib-0037] Preston, J. C. , J. Zhong , M. McKeown , M. den Bakker , and J. Friedman . 2016. “Comparative Transcriptomics Indicates a Role for Short Vegetative Phase (SVP) Genes in *Mimulus guttatus* Vernalization Response.” G3, Genes|Genomes|Genetics 6: 1239–1249. 10.1534/g3.115.026468 PMC4856076 26921300

[pld370202-bib-0038] Prodhan, M. A. , J. Pariasca‐Tanaka , Y. Ueda , P. E. Hayes , and M. Wissuwa . 2022. “Comparative Transcriptome Analysis Reveals a Rapid Response to Phosphorus Deficiency in a Phosphorus‐Efficient Rice Genotype.” Scientific Reports 12: 9460. 10.1038/s41598-022-13709-w PMC9177723 35676419

[pld370202-bib-0039] Qiao, Q. , Q. Wang , H. Sun , Y. Zhong , J. Huang , and T. Zhang . 2016. “Comparative Transcriptomics of Strawberries (*Fragaria* spp.) Provides Insights Into Evolutionary Patterns.” Frontiers in Plant Science 7: 234015. 10.3389/fpls.2016.01839 PMC5156730 28018379

[pld370202-bib-0040] Robinson, M. D. , D. J. McCarthy , and G. K. Smyth . 2010. “edgeR: A Bioconductor Package for Differential Expression Analysis of Digital Gene Expression Data.” Bioinformatics 26: 139–140. 10.1093/bioinformatics/btp616 PMC2796818 19910308

[pld370202-bib-0041] Semon, M. , and K. H. Wolfe . 2007. “Consequences of Genome Duplication.” Current Opinion in Genetics & Development 17: 505–512. 10.1016/j.gde.2007.09.007 18006297

[pld370202-bib-0042] Semon, M. , and K. H. Wolfe . 2008. “Preferential Subfunctionalization of Slow‐Evolving Genes After Allopolyploidization in *Xenopus laevis* .” Proceedings of the National Academy of Sciences of the United States of America 105: 8333–8338. 10.1073/pnas.0708705105 PMC2448837 18541921

[pld370202-bib-0043] Shannon, P. , A. Markiel , O. Ozier , et al. 2003. “Cytoscape: A Software Environment for Integrated Models of Biomolecular Interaction Networks.” Genome Research 13: 2498–2504. 10.1101/gr.1239303 PMC403769 14597658

[pld370202-bib-0045] Spokevicius, A. V. , J. Tibbits , P. Rigault , M. A. Nolin , C. Muller , and A. Merchant . 2017. “Medium Term Water Deficit Elicits Distinct Transcriptome Responses in *Eucalyptus* Species of Contrasting Environmental Origin.” BMC Genomics 18: 284. 10.1186/s12864-017-3664-z PMC5383985 28388878

[pld370202-bib-0046] Su, Y. , Z. Yu , S. Jin , et al. 2024. “Comprehensive Assessment of mRNA Isoform Detection Methods for Long‐Read Sequencing Data.” Nature Communications 15: 3972. 10.1038/s41467-024-48117-3 PMC11087464 38730241

[pld370202-bib-0047] Szklarczyk, D. , R. Kirsch , M. Koutrouli , et al. 2023. “The STRING Database in 2023: Protein–Protein Association Networks and Functional Enrichment Analyses for Any Sequenced Genome of Interest.” Nucleic Acids Research 51: D638–D646. 10.1093/nar/gkac1000 PMC9825434 36370105

[pld370202-bib-0048] Tian, F. , D.‐C. Yang , Y.‐Q. Meng , J. Jin , and G. Gao . 2020. “PlantRegMap: Charting Functional Regulatory Maps in Plants.” Nucleic Acids Research 48: D1104–D1113. 10.1093/nar/gkz1020 PMC7145545 31701126

[pld370202-bib-0049] Wang, B. , E. Tseng , M. Regulski , et al. 2016. “Unveiling the Complexity of the Maize Transcriptome by Single‐Molecule Long‐Read Sequencing.” Nature Communications 7: 11708. 10.1038/ncomms11708 PMC4931018 27339440

[pld370202-bib-0050] Ward, J. A. , L. Ponnala , and C. A. Weber . 2012. “Strategies for Transcriptome Analysis in Nonmodel Plants.” American Journal of Botany 99: 267–276. 10.3732/ajb.1100334 22301897

[pld370202-bib-0051] Wickham, H. 2016. “Data Analysis.” In ggplot2: Elegant Graphics for Data Analysis, 189–201. Springer.

[pld370202-bib-0052] Woo, J. , C. R. MacPherson , J. Liu , et al. 2012. “The Response and Recovery of the *Arabidopsis thaliana* Transcriptome to Phosphate Starvation.” BMC Plant Biology 12: 62. 10.1186/1471-2229-12-62 PMC3520718 22553952

[pld370202-bib-0053] Wu, P. , L. Ma , X. Hou , et al. 2003. “Phosphate Starvation Triggers Distinct Alterations of Genome Expression in *Arabidopsis* Roots and Leaves.” Plant Physiology 132: 1260–1271. 10.1104/pp.103.021022 PMC167066 12857808

[pld370202-bib-0054] Wu, Q. , W. Tong , H. Zhao , et al. 2022. “Comparative Transcriptomic Analysis Unveils the Deep Phylogeny and Secondary Metabolite Evolution of 116 Camellia Plants.” Plant Journal 111: 406–421. 10.1111/tpj.15799 35510493

[pld370202-bib-0055] Xia, E. H. , J. J. Jiang , H. Huang , L. P. Zhang , H. B. Zhang , and L. Z. Gao . 2014. “Transcriptome Analysis of the Oil‐Rich Tea Plant, *Camellia oleifera*, Reveals Candidate Genes Related to Lipid Metabolism.” PLoS ONE 9: e104150. 10.1371/journal.pone.0104150 PMC4138098 25136805

[pld370202-bib-0056] Xiang, Y. , C. H. Huang , Y. Hu , et al. 2017. “Evolution of Rosaceae Fruit Types Based on Nuclear Phylogeny in the Context of Geological Times and Genome Duplication.” Molecular Biology and Evolution 34: 262–281. 10.1093/molbev/msw242 PMC5400374 27856652

[pld370202-bib-0057] Yang, Y. , M. J. Moore , S. F. Brockington , et al. 2015. “Dissecting Molecular Evolution in the Highly Diverse Plant Clade Caryophyllales Using Transcriptome Sequencing.” Molecular Biology and Evolution 32: 2001–2014. 10.1093/molbev/msv081 PMC4833068 25837578

[pld370202-bib-0058] Yang, Z. , and R. Nielsen . 2002. “Codon‐Substitution Models for Detecting Molecular Adaptation at Individual Sites Along Specific Lineages.” Molecular Biology and Evolution 19: 908–917. 10.1093/oxfordjournals.molbev.a004148 12032247

[pld370202-bib-0059] Zhang, Z. , J. Li , X.‐Q. Zhao , J. Wang , G. K.‐S. Wong , and J. Yu . 2006. “KaKs_Calculator: Calculating Ka and Ks Through Model Selection and Model Averaging.” Genomics, Proteomics & Bioinformatics 4: 259–263. 10.1016/S1672-0229(07)60007-2 PMC5054075 17531802

[pld370202-bib-0060] Zhao, L. , X. Gong , J. Gao , et al. 2019. “Transcriptomic and Evolutionary Analyses of White Pear (*Pyrus bretschneideri*) *β*‐Amylase Genes Reveals Their Importance for Cold and Drought Stress Responses.” Gene 689: 102–113. 10.1016/j.gene.2018.11.092 30576803

[pld370202-bib-0061] Zhao, L. , N. Zhang , P. F. Ma , Q. Liu , D. Z. Li , and Z. H. Guo . 2013. “Phylogenomic Analyses of Nuclear Genes Reveal the Evolutionary Relationships Within the BEP Clade and the Evidence of Positive Selection in *Poaceae* .” PLoS ONE 8: e64642. 10.1371/journal.pone.0064642 PMC3667173 23734211

