# Supplementary material for: Long‐Read Reference Transcriptomes for Five Species of Rosaceae Tribe Spiraeeae: A Resource for Investigating Sequence Divergence, Species‐Associated Expression Variation, and Leaf‐Developmental Candidate Genes
Source: Plant Direct. 2026 Nov 2;10(11):e70202. doi: 10.1002/pld3.70202 (PMC13728836; doi:10.1002/pld3.70202)
Supplement: Supplementary file 1 [file PLD3-10-e70202-s001.pdf]

Supporting Information

Long-Read Reference Transcriptomes for Five Species of Rosaceae Tribe Spiraeeae: A Resource for Investigating Sequence Divergence, Species-Associated Expression Variation, and Leaf-Developmental Candidate Genes

Armin M. Mafakheri, Daniel Potter

Contents

Supplemental Figure 1. Up- and down-regulated differentially expressed transcripts (DETs) across pairwise species contrasts.

Supplemental Figure 2. Volcano plot of differential transcript expression between *A. dioicus* and *A. aethusifolius*.

Supplemental Figure 3. Volcano plot of differential transcript expression between *A. aethusifolius* and *S. splendens*.

Supplemental Figure 4. Volcano plot of differential transcript expression between *A. dioicus* and *P. caespitosum*.

Supplemental Figure 5. Volcano plot of differential transcript expression between *A. dioicus* and *S. lucida*.

Supplemental Figure 6. Volcano plot of differential transcript expression between *A. dioicus* and *S. splendens*.

Supplemental Figure 7. Volcano plot of differential transcript expression between *S. splendens* and *P. caespitosum*.

Supplemental Figure 8. Volcano plot of differential transcript expression between *S. lucida* and *S. splendens*.

Supplemental Figure 9. Scale-free topology fit and mean connectivity analysis for WGCNA soft-threshold selection.

Supplemental Figure 10. Weighted gene co-expression network analysis (WGCNA).

Supplemental Figure 11. Heatmap of module–trait relationships showing correlations between module eigengenes and species identity.

Supplemental Figure 12. Cytoscape network visualization of the cyan WGCNA module.

Supplemental Figure 13. Cytoscape network visualization of the dark gray WGCNA module.

Supplemental Figure 14. Cytoscape network visualization of the green–yellow WGCNA module.

Supplemental Figure 15. Cytoscape network visualization of the light green WGCNA module.

Supplemental Figure 16. Comparative analysis of nonsynonymous (Ka) and synonymous (Ks) substitution rates for additional species pairs.

Supplemental Table 1. Characteristics of the genera of Rosaceae tribe Spiraeeae (from Potter et al. 2007).

Supplemental Table 2. Taxa sampled for this study. Voucher specimens are all deposited in the herbarium of the UC Davis Center for Plant Diversity (DAV). Information from Lis (2014 a, b) and Potter and Rosatti (2012). Biological Replicates refers to number of individuals sampled.

Supplemental Table 3. Functional annotation of top-ranked genes identified in volcano plot analyses across species contrasts.

Supplemental Table 4. Functional annotation summary of candidate loci with elevated Ka/Ks ratios.

Supplemental Table 5. Leaf-developmental regulator transcripts recovered in the combined reference, with best-hit gene, family assignment, and percent identity.

Supplemental Table 6. Ortholog pairs evaluated and retained after Ks filtering in each pairwise species comparison.

Supplemental Figure 1

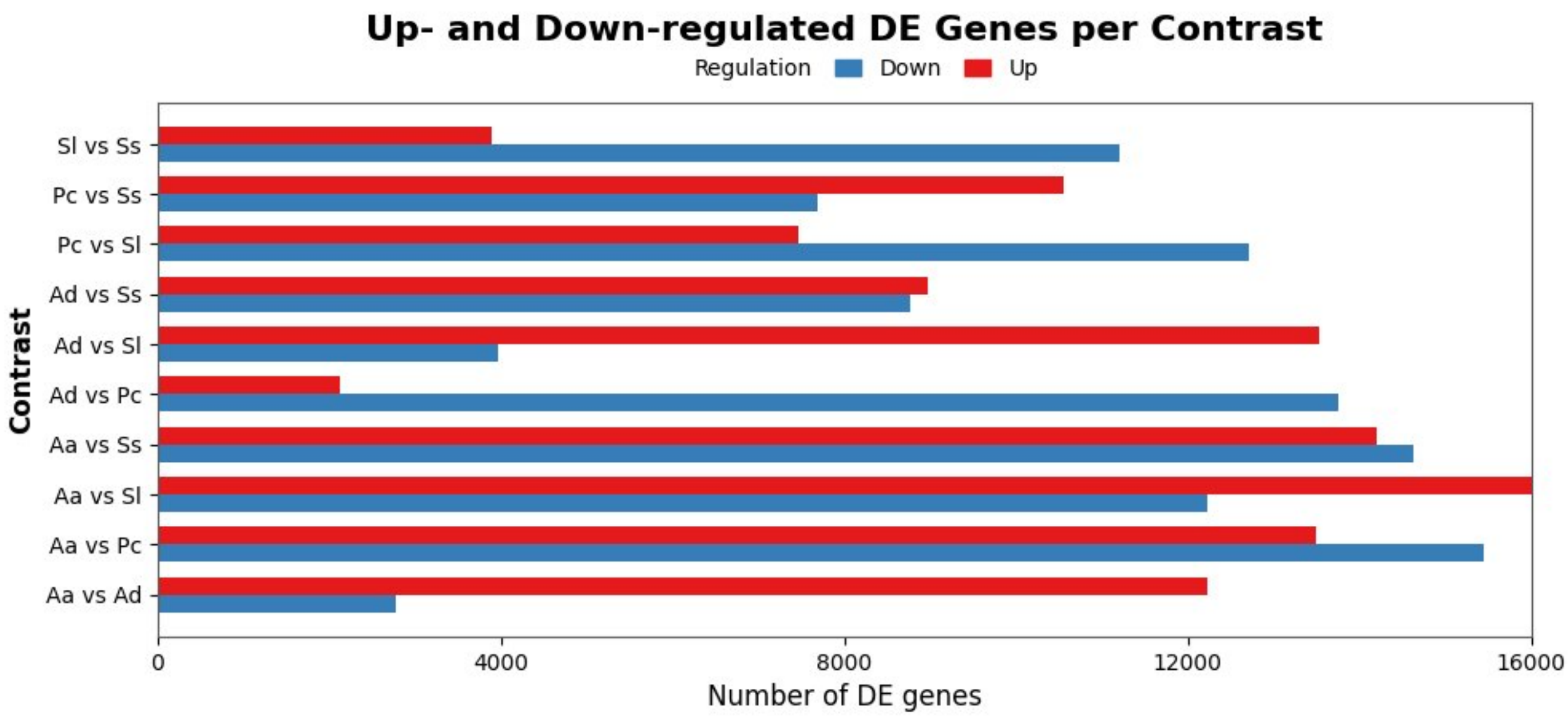

Supplemental Figure 1. Up- and down-regulated differentially expressed transcripts (DETs) across pairwise species contrasts. Horizontal bars show the number of DETs that are significantly upregulated (red) or downregulated (blue) in the first species listed in each contrast relative to the second, based on limma–voom analysis of filtered, TMM-normalized counts (|log2FC| ≥ 2; FDR < 0.01). Species abbreviations: Aa, *Aruncus aethusifolius*; Ad, *Aruncus dioicus*; Pc, *Petrophytum caespitosum*; Sl, *Spiraea lucida*; Ss, *Spiraea splendens*.

Supplemental Figure 2

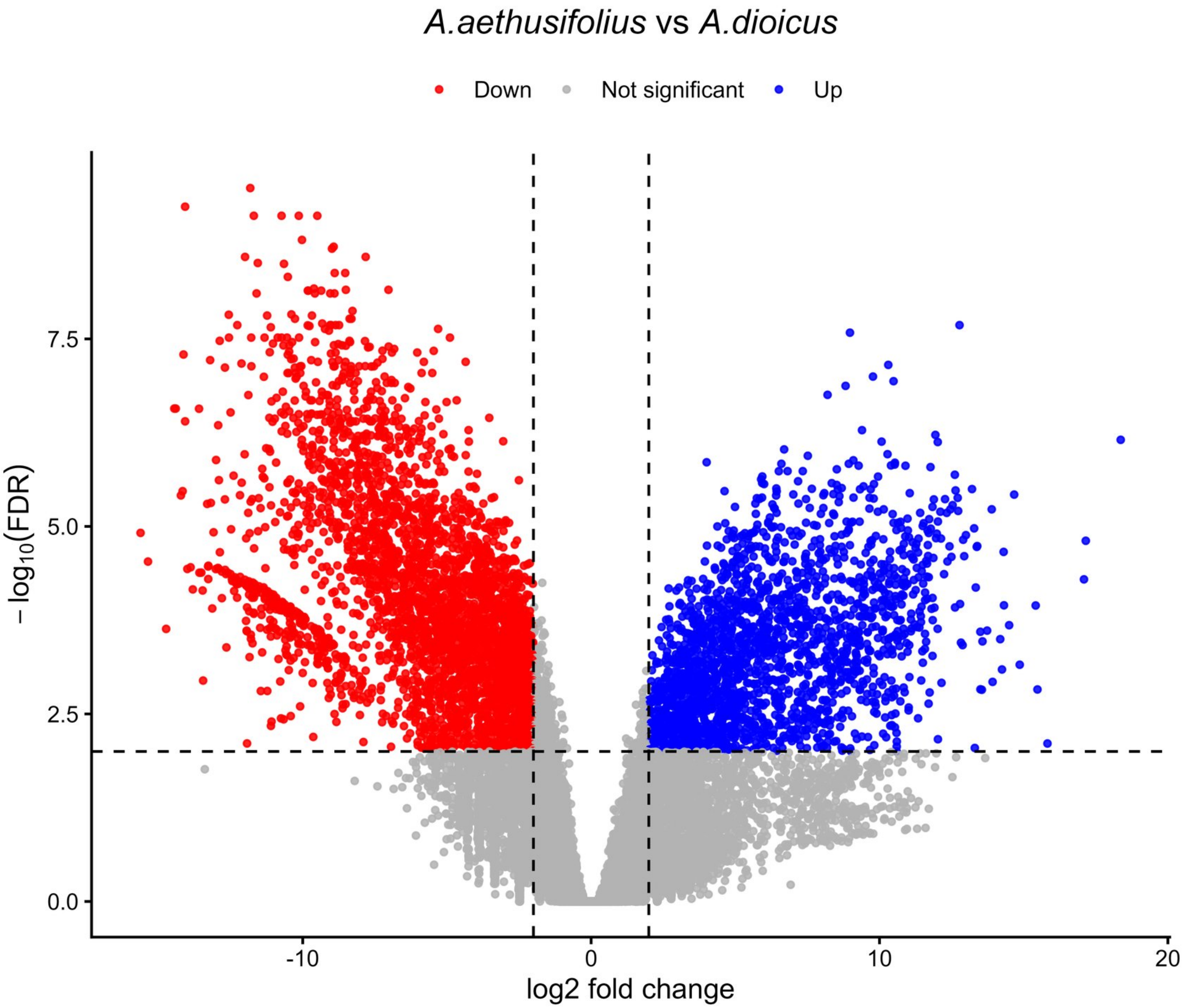

Supplemental Figure 2. Volcano plot of differential transcript expression between *A. dioicus* and *A. aethusifolius*. Visualization parameters and significance thresholds are consistent with those described in the Methods.

Supplemental Figure 3

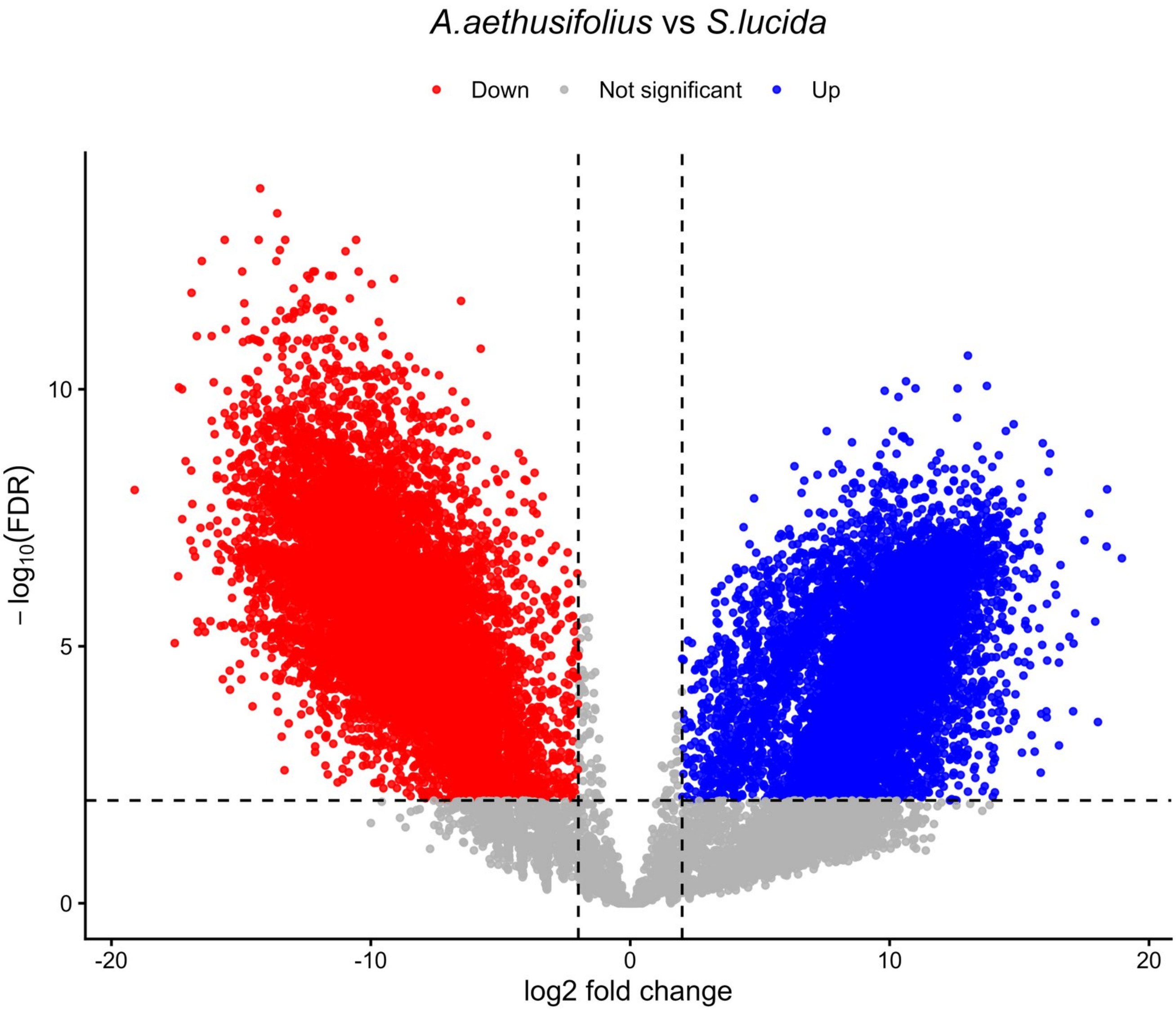

Supplemental Figure 3. Volcano plot of differential transcript expression between *A. aethusifolius* and *S. splendens*. Visualization parameters and significance thresholds are consistent with those described in the Methods.

Supplemental Figure 4

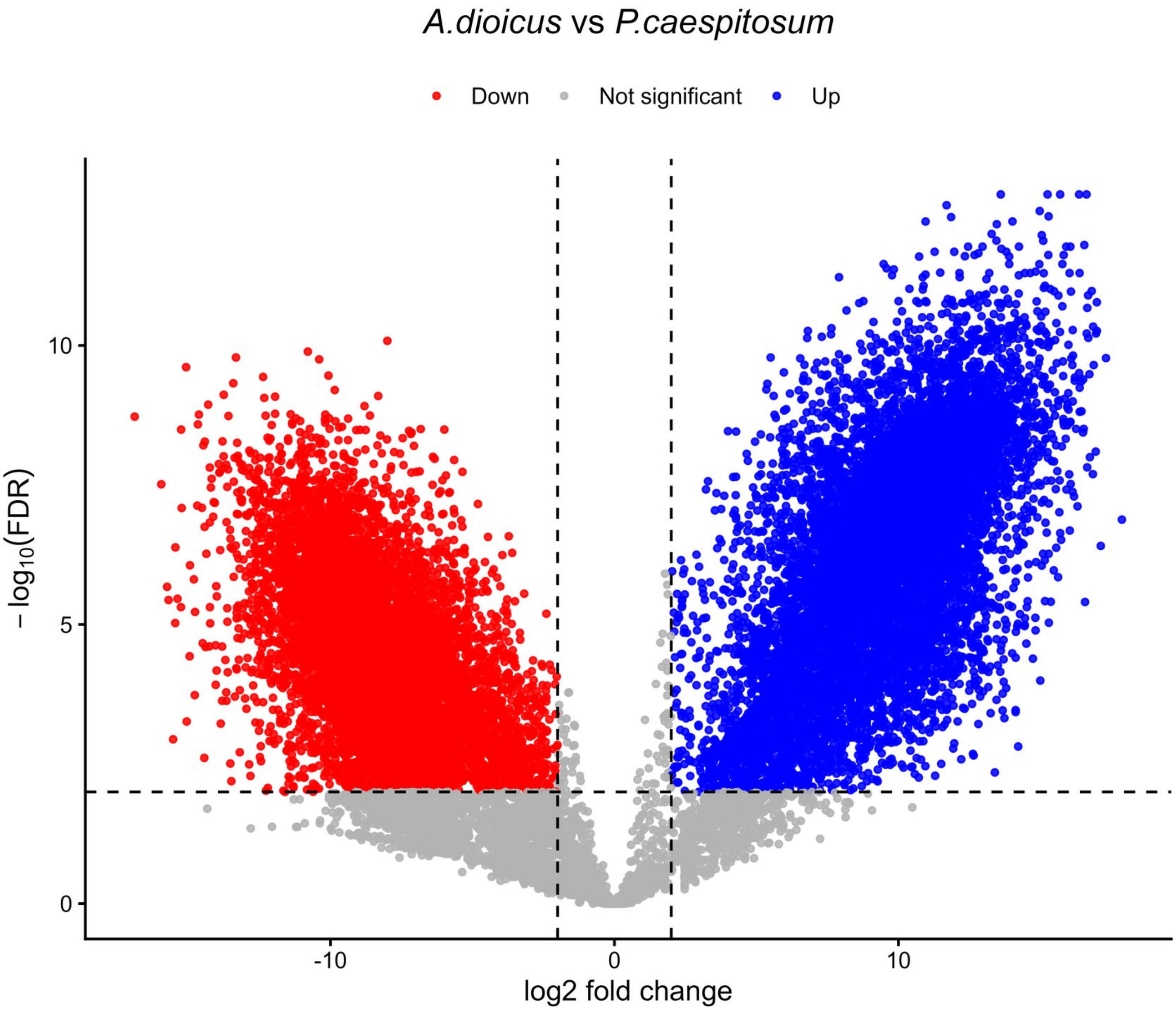

Supplemental Figure 4. Volcano plot of differential transcript expression between *A. dioicus* and *P. caespitosum*. Visualization parameters and significance thresholds are consistent with those described in the Methods.

Supplemental Figure 5

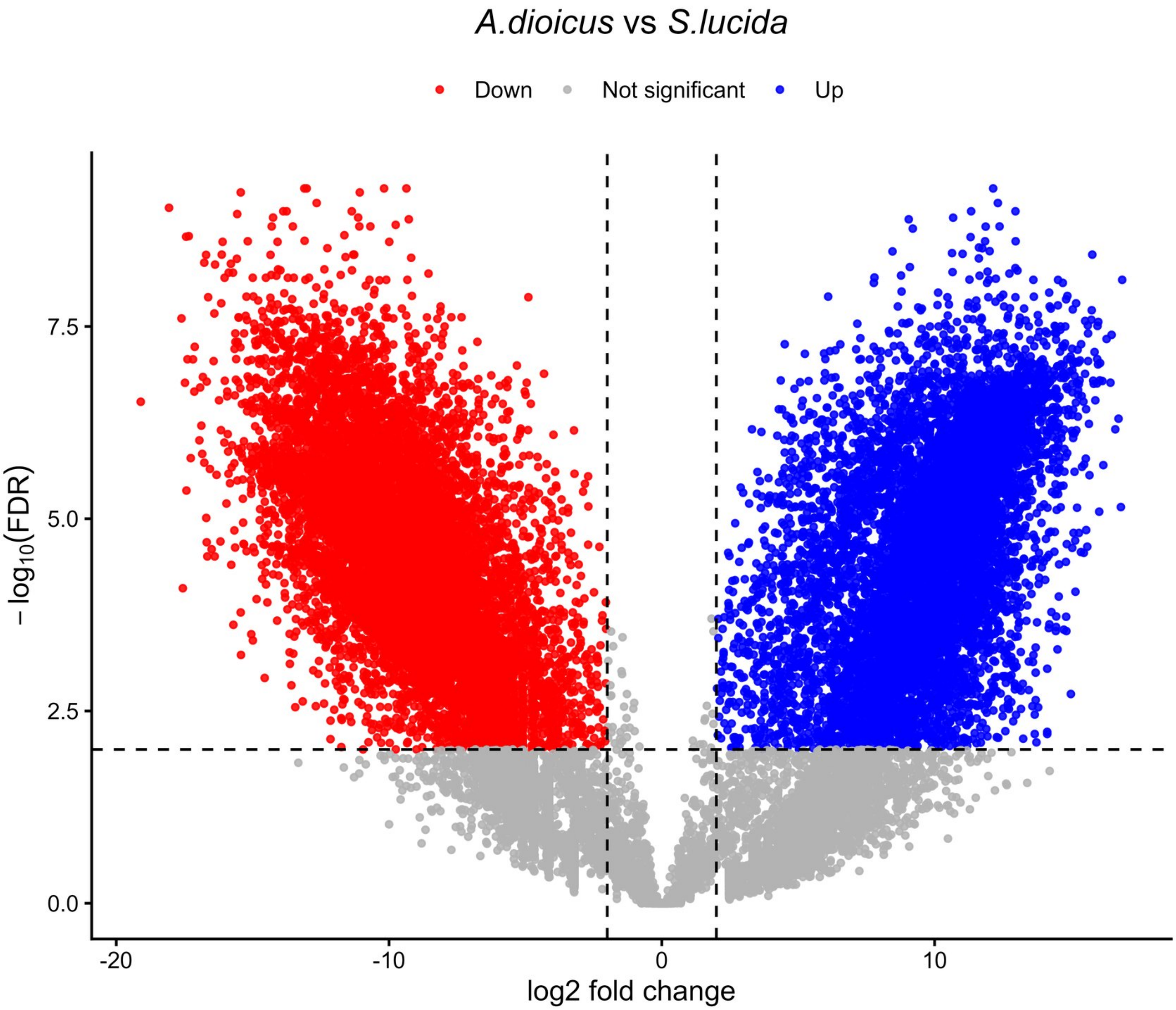

Supplemental Figure 5. Volcano plot of differential transcript expression between *A. dioicus* and *S. lucida*. Visualization parameters and significance thresholds are consistent with those described in the Methods.

Supplemental Figure 6

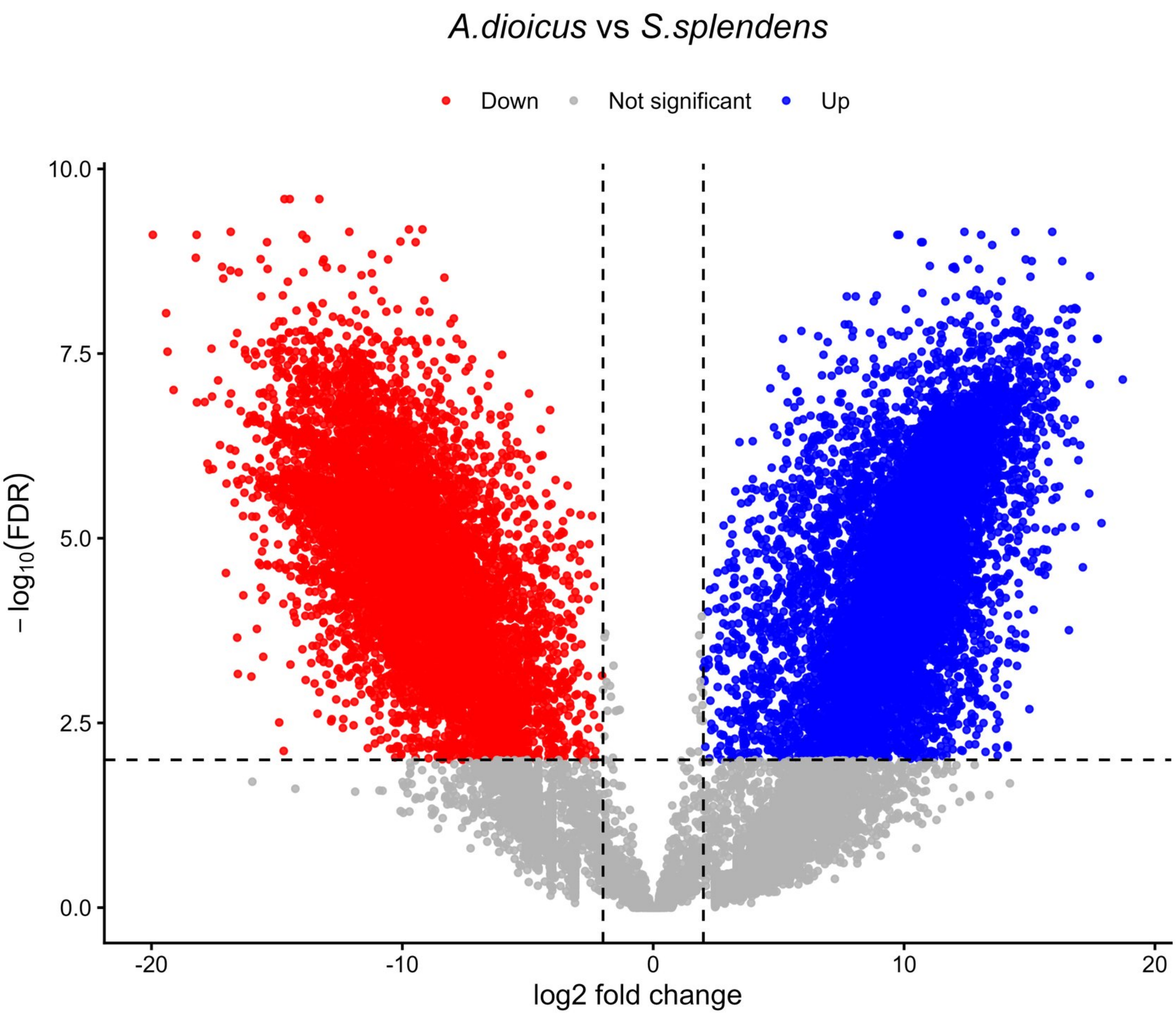

Supplemental Figure 6. Volcano plot of differential transcript expression between *A. dioicus* and *S. splendens*. Visualization parameters and significance thresholds are consistent with those described in the Methods.

Supplemental Figure 7

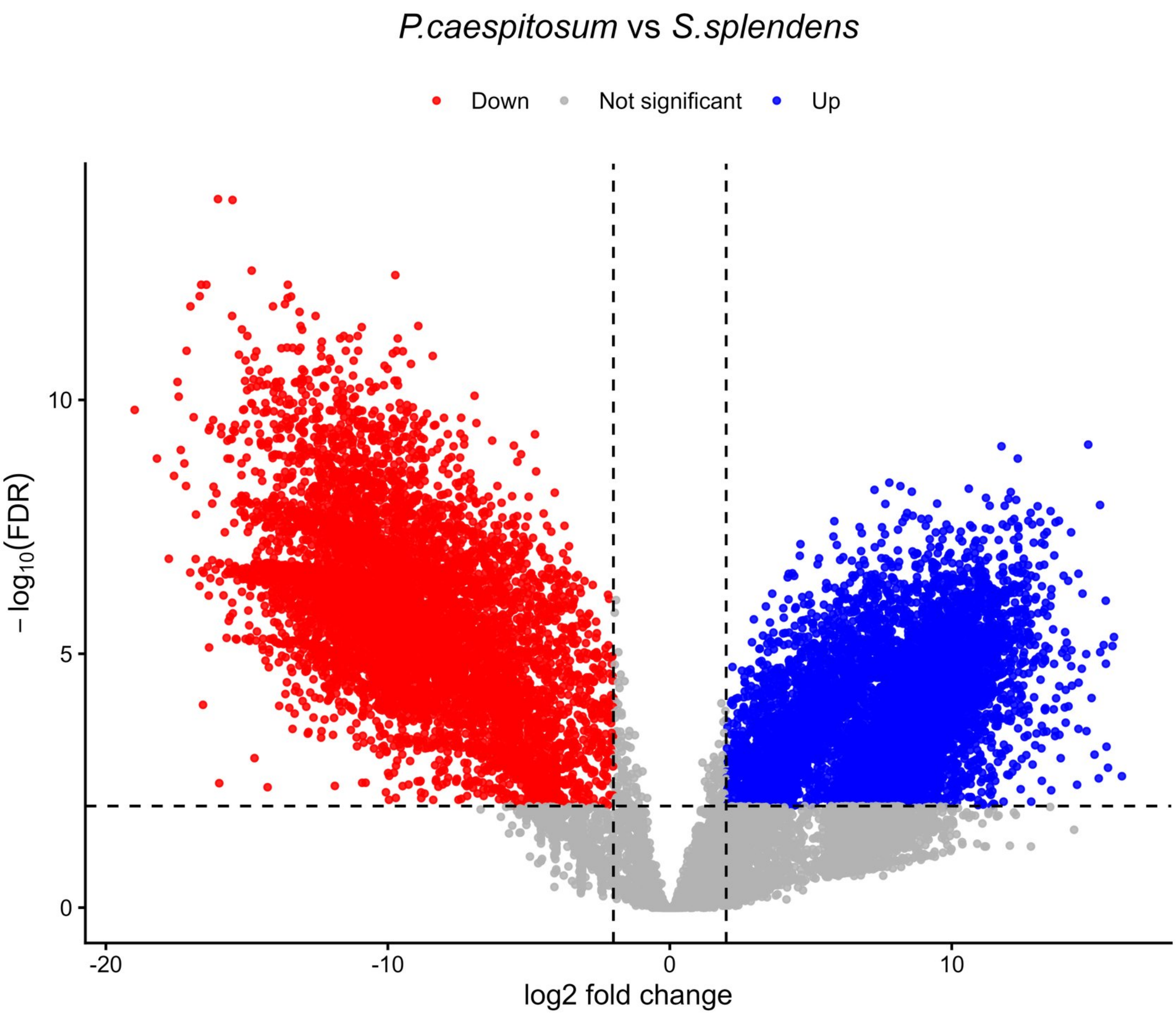

Supplemental Figure 7. Volcano plot of differential transcript expression between *S. splendens* and *P. caespitosum*. Visualization parameters and significance thresholds are consistent with those described in the Methods.

Supplemental Figure 8

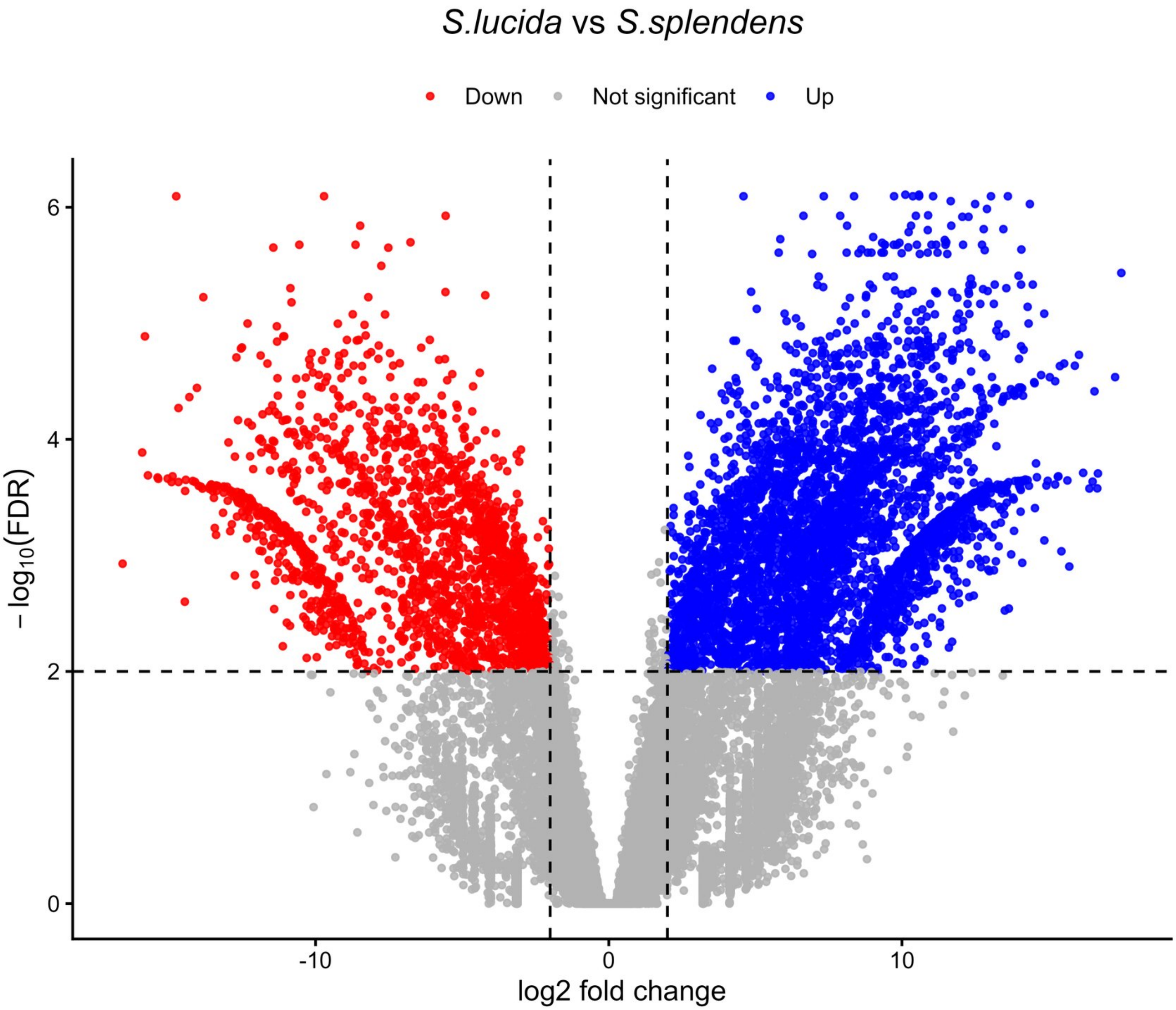

Supplemental Figure 8. Volcano plot of differential transcript expression between *S. lucida* and *S. splendens*. Visualization parameters and significance thresholds are consistent with those described in the Methods.

Supplemental Figure 9

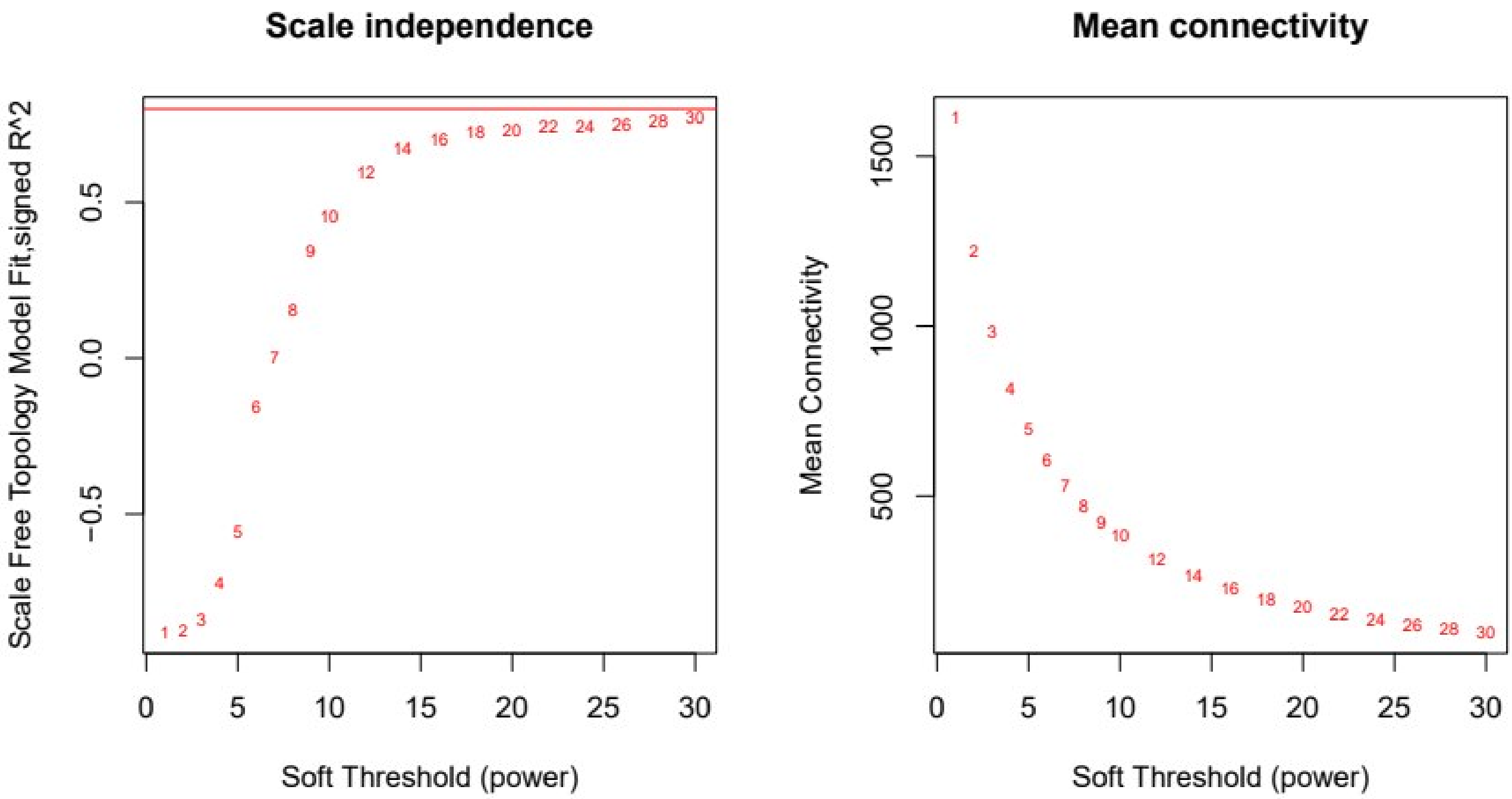

Supplemental Figure 9. Scale-free topology fit and mean connectivity analysis for WGCNA soft-threshold selection. Left panel shows the scale-free topology model fit (signed R^2) across candidate soft-thresholding powers, and the right panel shows the corresponding mean connectivity.

Supplemental Figure 10

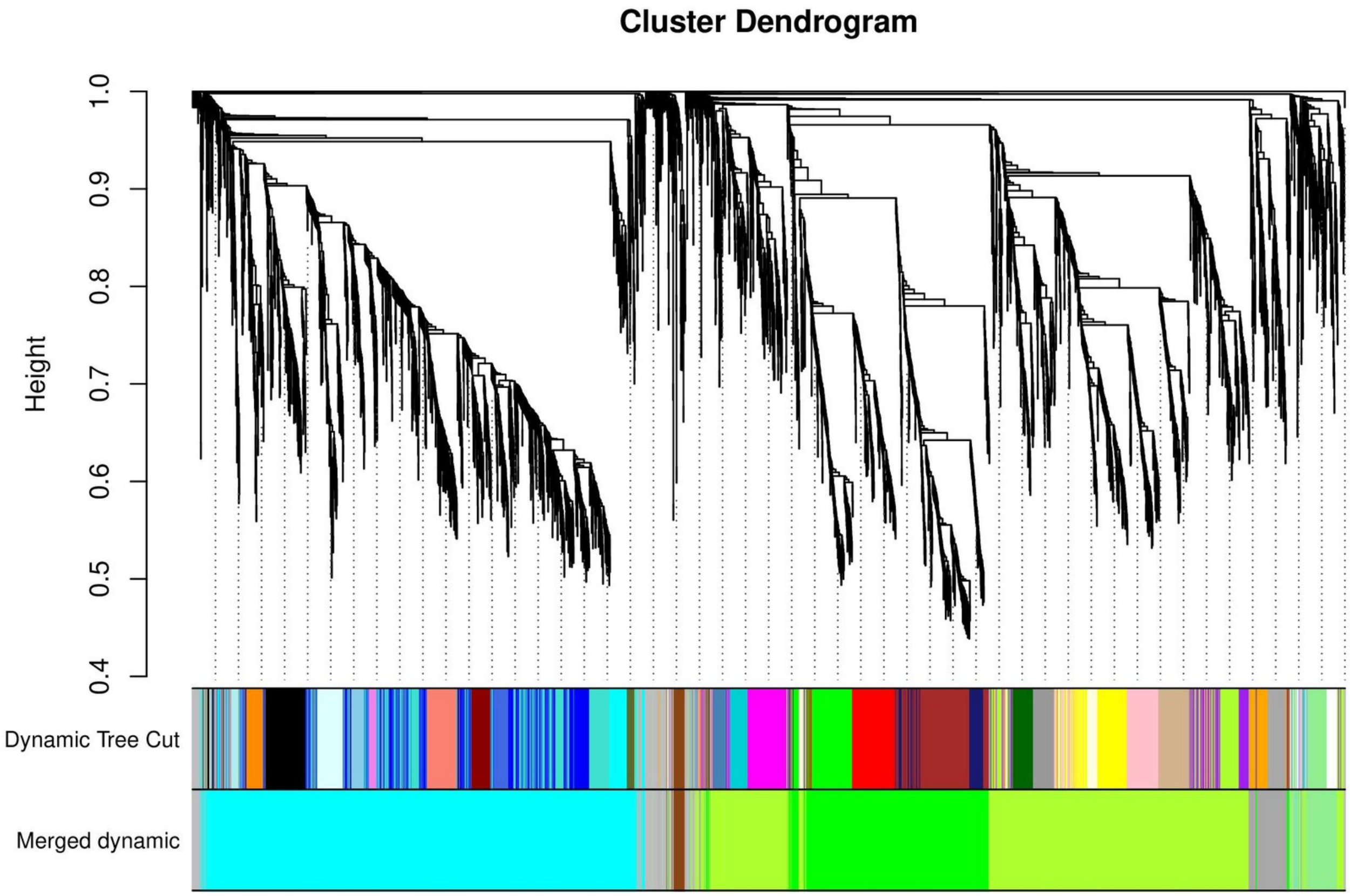

Supplemental Figure 10. Weighted gene co-expression network analysis (WGCNA). Hierarchical clustering dendrogram of transcripts based on topological overlap, with module assignments shown below. Colors indicate gene modules identified using dynamic tree cutting and subsequent module merging based on eigengene similarity.

Supplemental Figure 11

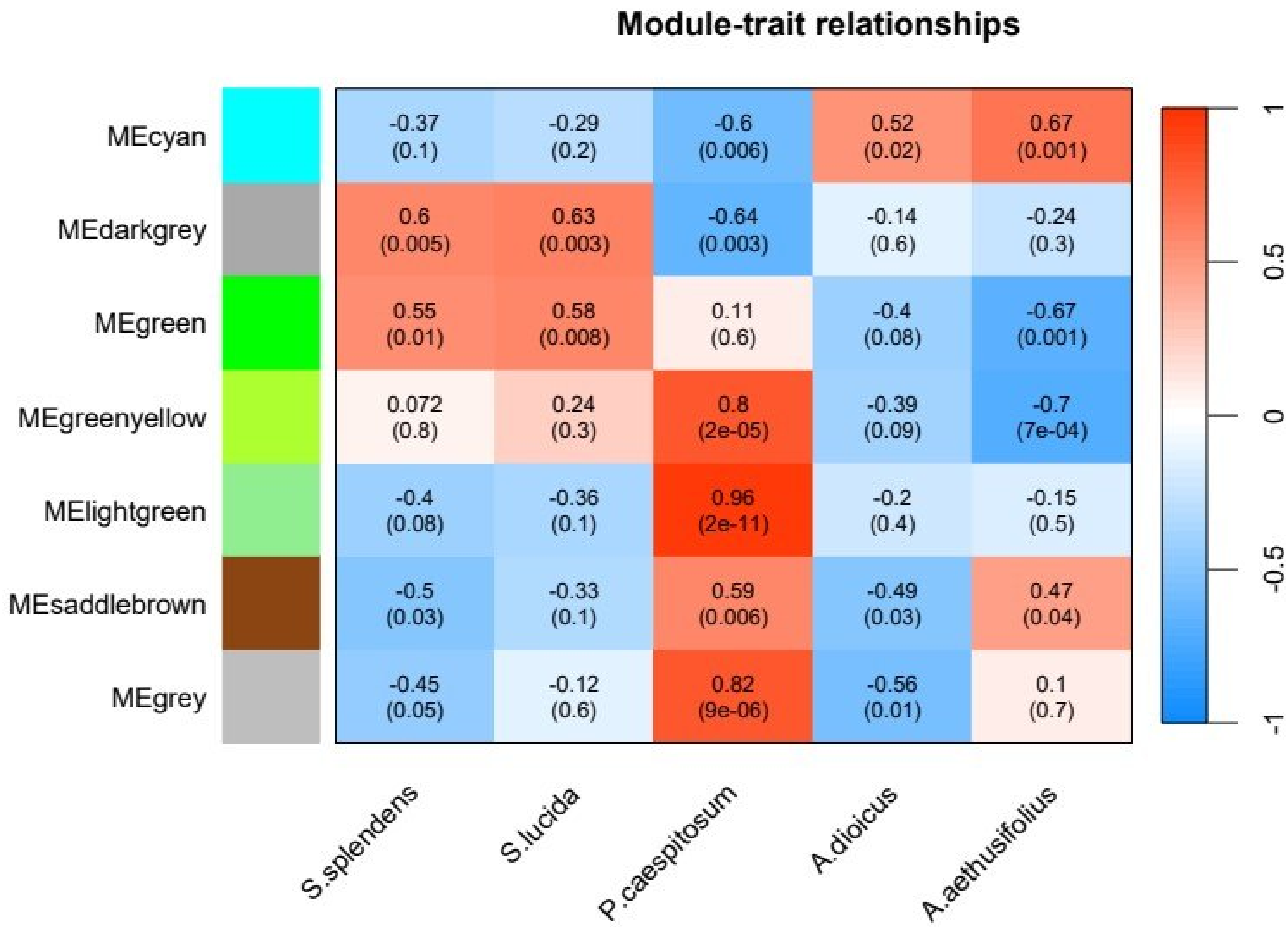

Supplemental Figure 11. Heatmap of module–trait relationships showing correlations between module eigengenes and species identity. Values represent Pearson correlation coefficients, with corresponding p-values shown in parentheses. Color intensity reflects the strength and direction of the correlation.

Supplemental Figure 12

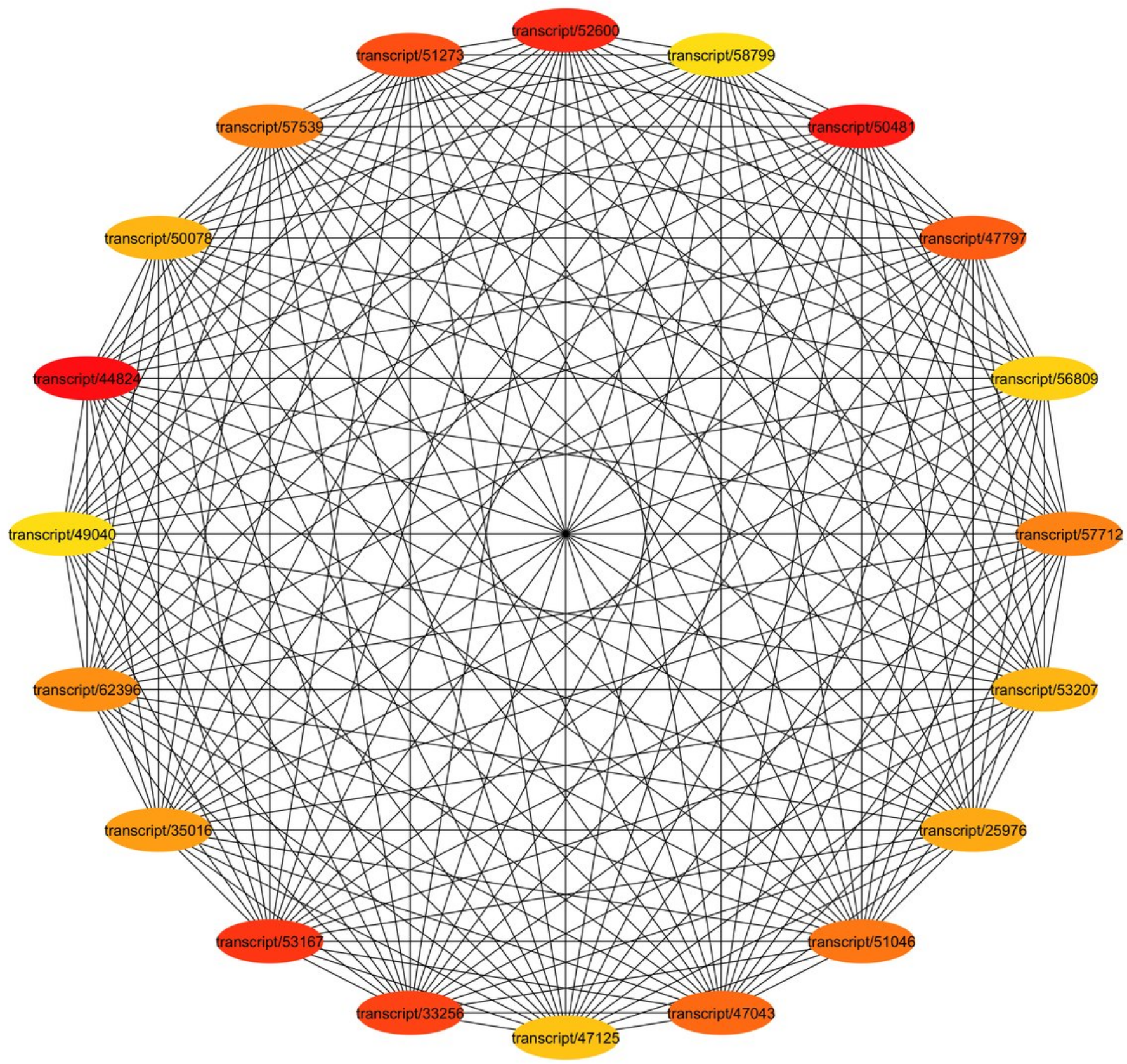

Supplemental Figure 12. Cytoscape network visualization of the cyan WGCNA module. Nodes represent transcripts and edges represent co-expression relationships, visualized to highlight highly connected genes within the module.

Supplemental Figure 13

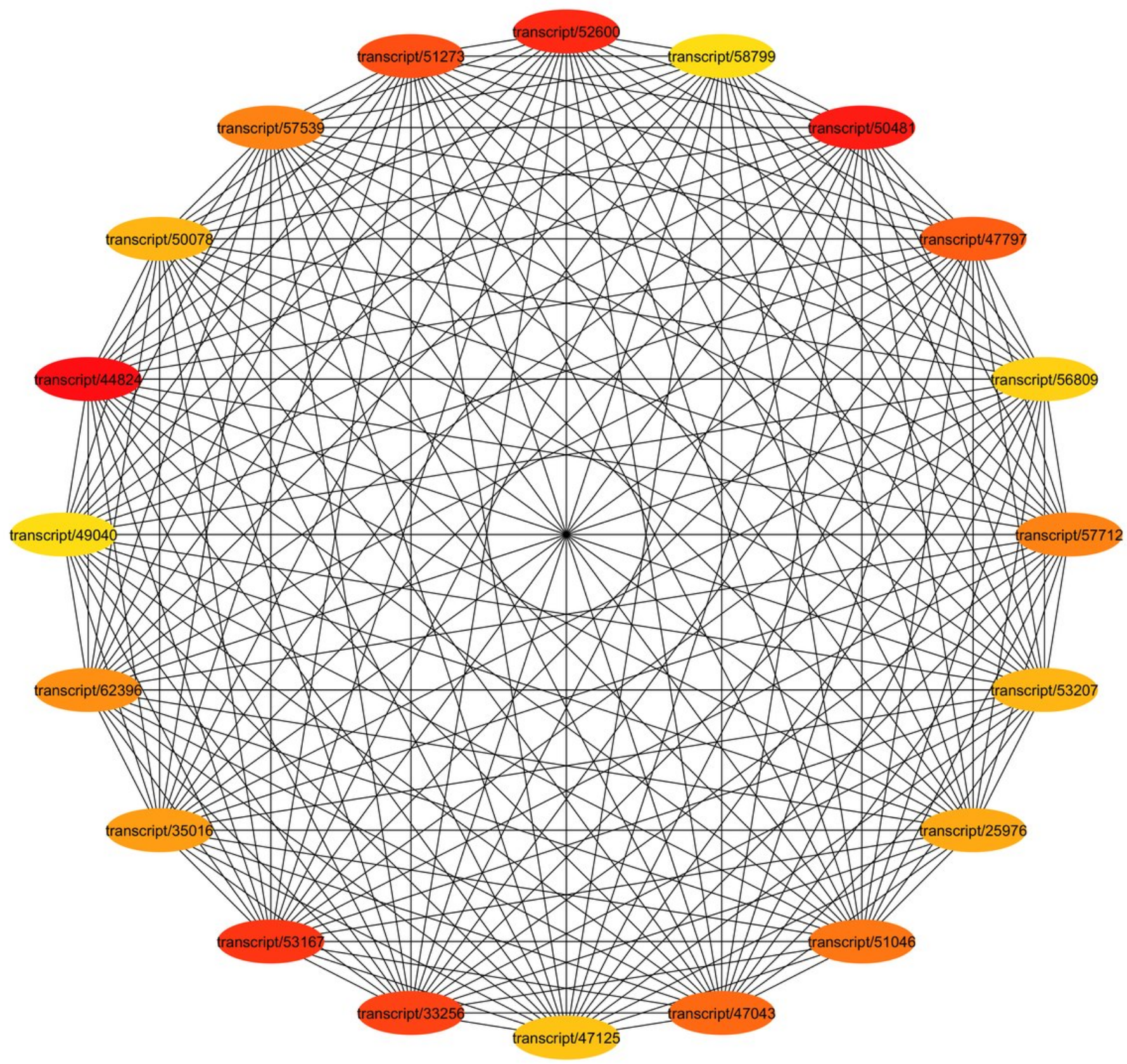

Supplemental Figure 13. Cytoscape network visualization of the dark gray WGCNA module. Nodes represent transcripts and edges represent co-expression relationships, visualized to highlight highly connected genes within the module.

Supplemental Figure 14

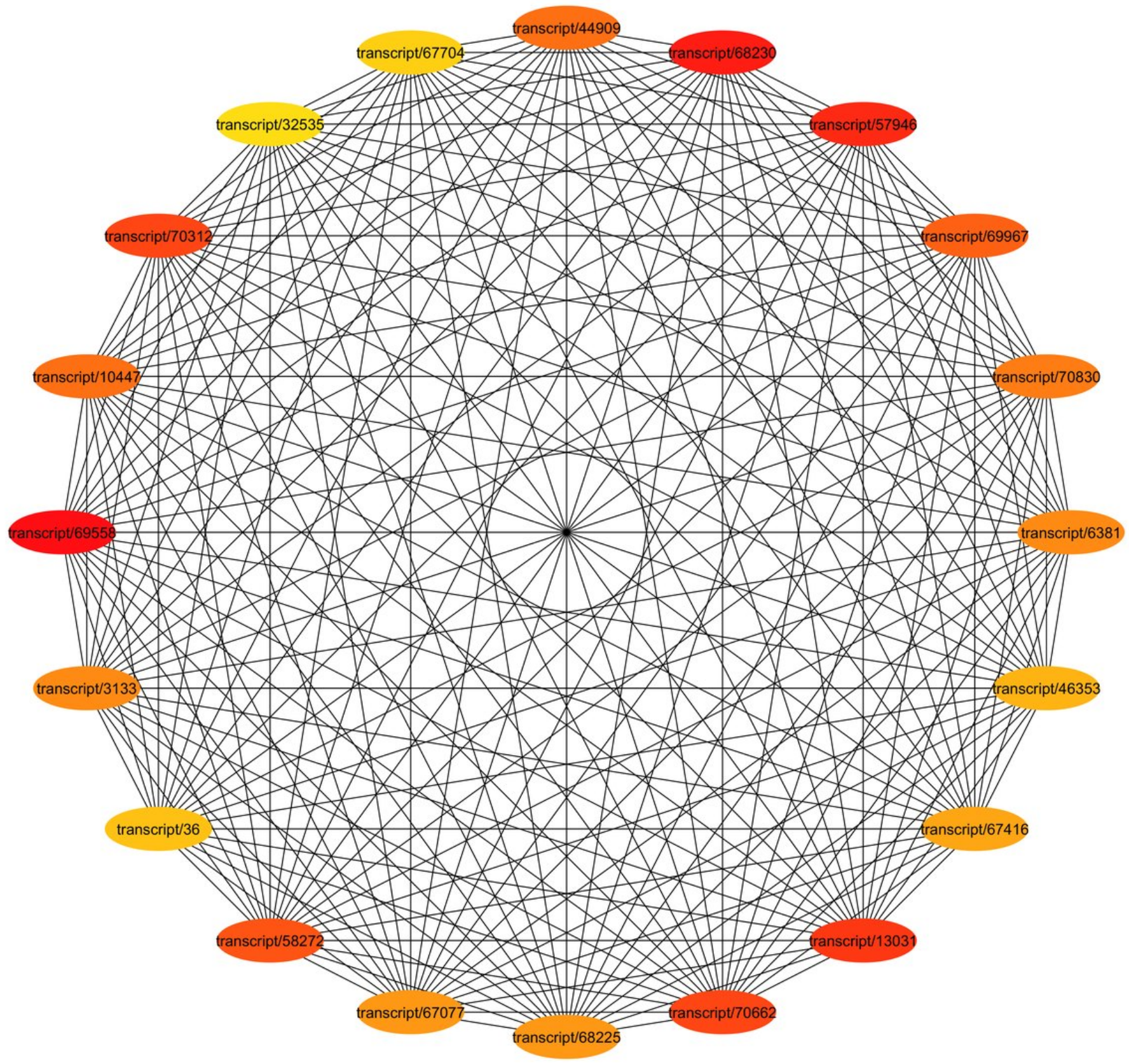

Supplemental Figure 14. Cytoscape network visualization of the green–yellow WGCNA module. Network structure illustrates co-expression relationships among transcripts identified within this module.

Supplemental Figure 15

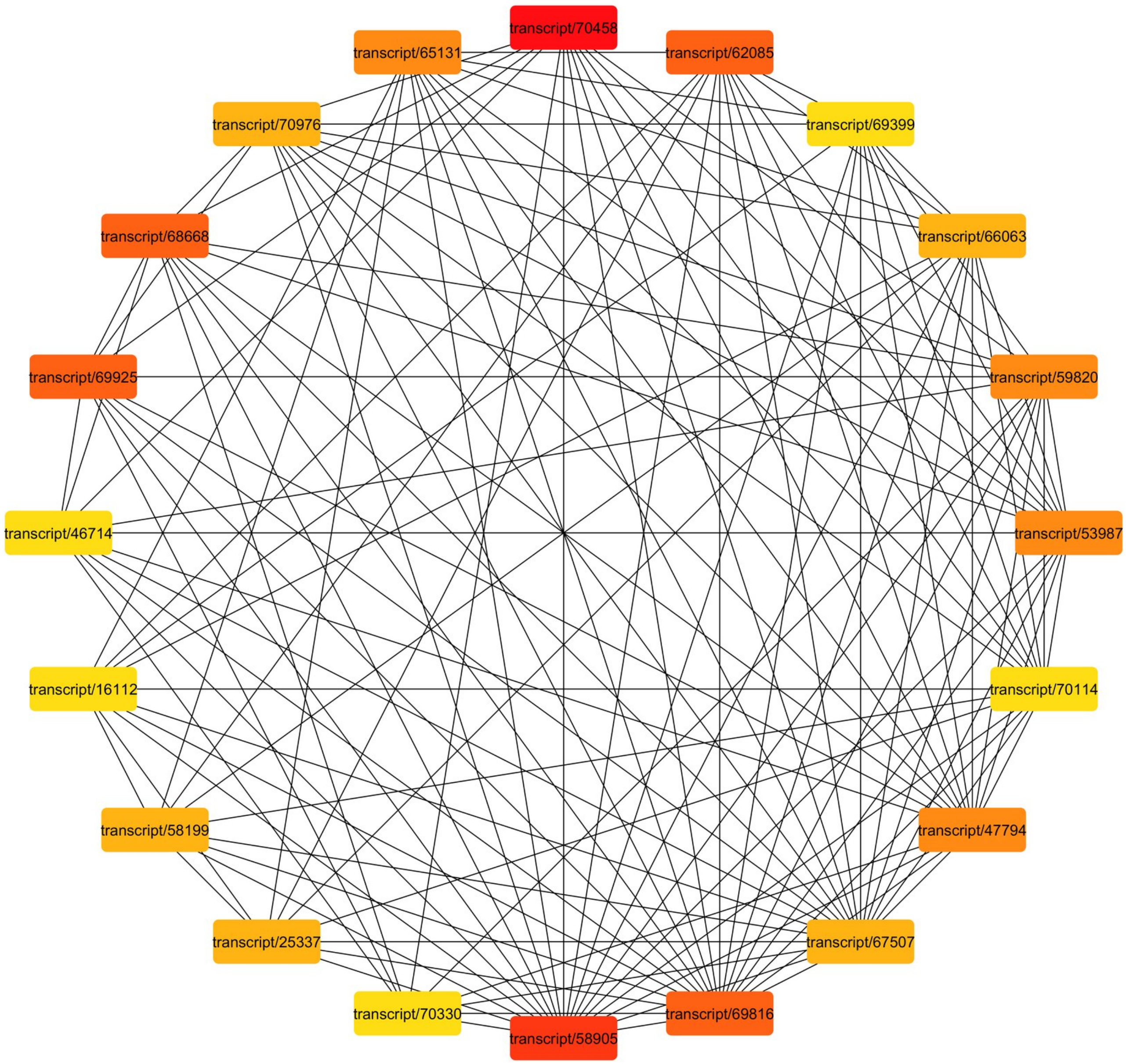

Supplemental Figure 15. Cytoscape network visualization of the light green WGCNA module. Nodes and edges represent transcript-level co-expression relationships inferred from the WGCNA adjacency matrix.

Supplemental Figure 16

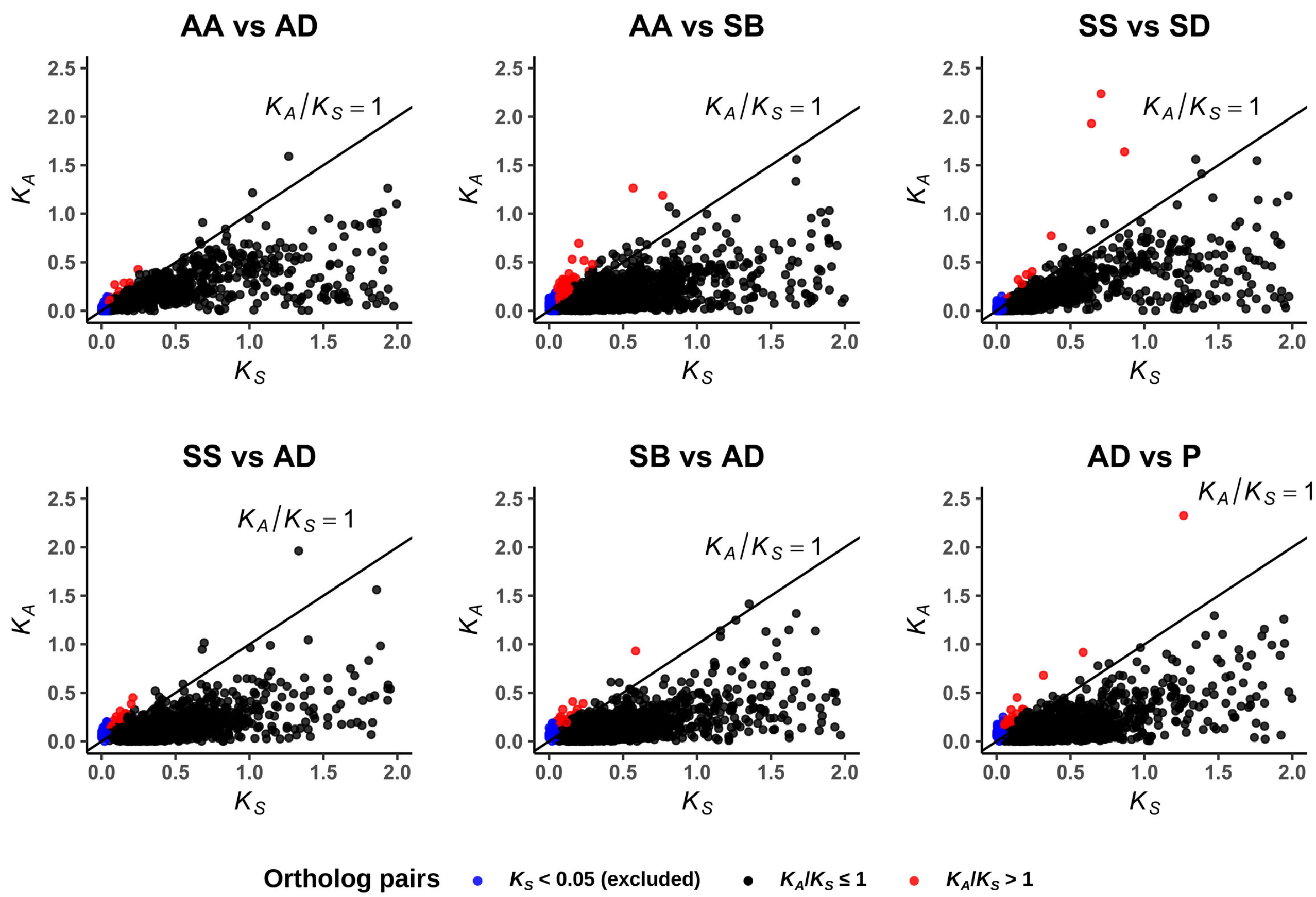

Supplemental Figure 16. Comparative analysis of nonsynonymous (Ka) and synonymous (Ks) substitution rates for the remaining six species pairs. Each scatter plot shows the nonsynonymous substitution rate (Ka) plotted against the synonymous substitution rate (Ks) for orthologous gene pairs. The diagonal line indicates Ka = Ks (Ka/Ks = 1), corresponding to neutral expectations. Genes with Ka/Ks > 1 were flagged as candidate loci with elevated Ka/Ks ratios under our screening criteria (red points). Blue points represent ortholog pairs with very low Ks values (Ks < 0.05), which were excluded from inference due to unstable rate estimates but are shown for completeness.

Supplemental Table 1

Supplemental Table 1. Characteristics of the genera of Rosaceae tribe Spiraeeae (from Potter et al. 2007).

Genus	Number of Species	Habit	Leaves	Inflorescence	Distribution
Aruncus Adans.	1-5	perennial herb	2-3 pinnately compound, serrate	panicle	n temp
Holodiscus Maxim.	2-5	erect shrub	simple serrate	panicle	w N Am - n S Am
Kelseya Rydb.	1	cespitose shrub	simple entire	solitary	MT, WY
Luetkea Bong.	1	trailing subshrub	biternate	raceme	w N Am
Pentactina Nakai	1	erect shrub	simple serrate	raceme	Korea
Petrophytum Rydb.	3	cespitose shrub	simple entire	raceme	w N Am
Sibiraea Maxim.	4-7	erect shrub	simple entire	panicle	se Eu - e Asia
Spiraea L.	50-80	erect shrub	simple serrate	variable	n temp
Xerospiraea Henrickson	1	erect shrub	simple entire	raceme / panicle	Mexico

Supplemental Table 2

Supplemental Table 2. Taxa sampled for this study. Voucher specimens are all deposited in the herbarium of the UC Davis Center for Plant Diversity (DAV). Information from Lis (2014 a, b) and Potter and Rosatti (2012). Biological Replicates refers to number of individuals sampled.

Taxon	Native Range and Habitat	Leaf Size	Voucher Specimen	Voucher Specimen Details	Biological Replicates
Aruncus aethusifolius (H.Lév.) Nakai1	Jeju Island, South Korea, moist areas, 400–1800 m	5–16 × 5–15 cm	Potter 951	1 Sep 2019. 38.56858 N, -121.75378 W, 15 m. Yolo Co., CA, from potted plant cultivated at 417 Iris Place, Davis; originally from Digging Dog Nursery, Albion, CA	6
Aruncus dioicus (Walter) Fernald var. dioicus	Southeastern US, moist areas,	15–60 × 10–20 cm	Potter 952	1 Sep 2019. 38.56858 N, -121.75378 W, 15 m. Yolo Co., CA, from potted plant cultivated at 417 Iris Place, Davis; originally from Gardens of the Blue Ridge Nursery, Newland, NC	3
	10–1500 m				
Petrophytum caespitosum (Nutt.) Rydbg. subsp. caespitosum	Western North America, rocky outcrops and cliffs, 100–3000 m	0.4–1.8 × 0.2–0.4 cm	Potter 947	22 Aug 2019. 43.94952 N, -113.44591W, 1950 m. Butte Co., ID, Pass Creek Road, ca. 6 miles north of intersection with Highway 93 at Leslie. On limestone cliffs on the east side of the road	5
Spiraea lucida Douglas ex Greene	Western North America, open woods and meadows, streams, rocky outcrops	2–8 × 1–3 cm	Potter 950	1 Sep 2019. 38.56858 N, -121.75378 W, 15 m. Yolo Co., CA. From potted plant cultivated at 417 Iris Place, Davis; originally from Dancing Oaks Nursery, Monmouth, OR.	3
	10–3300 m				
Spiraea	Western North America, open woods and meadows, streams, rocky outcrops, 500–3500 m	1–4 × 1–3 cm	Potter 946	20 Aug 2019. 40.69959 N, -121.60355W; 2200 m. Shasta Co., CA, Thousand Lakes Wilderness, southwest corner of Magee Lake.	3
splendens Baumann ex K. Koch					
1 Some recent treatments (e.g., Mellichamp 2014) adopt the view that Aruncus comprises just one widely distributed and highly polymorphic species, A. dioicus, Here we follow treatments that divide the taxon into segregate species (e.g., Yun et al. 2017). Aruncus aethusifolius is distinguished from other taxa in the genus due to its shorter stature and highly dissected leaflets.					

Supplemental Table 3

Supplemental Table 3. Functional annotation of top-ranked genes identified in volcano plot analyses across species contrasts.

**Ss_Aa**	
Gene.123	beta-hexosaminidase 2
Gene.797	Protein kinase superfamily protein
Gene.1939	Ribosomal protein L21
Gene.2272	SPA1-related 4
Gene.2680	RNA-binding protein 1
Gene.3398	MATE efflux family protein
Gene.3775	ACT-like protein tyrosine kinase family protein
Gene.4316	glutamate receptor 2.9
Gene.4625	Insulinase (Peptidase family M16) family protein
Gene.6141	CRM family member 2
Gene.6340	TCP domain protein 16
Gene.6352	Calcineurin-like metallo-phosphoesterase superfamily protein
Gene.6648	GTP-binding protein, HflX
Gene.6789	Phototropic-responsive NPH3 family protein
Gene.7129	ENTH/VHS family protein
Gene.7894	glycine-rich protein
Gene.8388	RING/U-box superfamily protein
Gene.8391	NRAMP metal ion transporter family protein
Gene.8636	conserved oligomeric Golgi complex component-related / COG complex component-like protein
Gene.8694	Thiamin diphosphate-binding fold (THDP-binding) superfamily protein
Gene.9659	GRAS family transcription factor
Gene.10023	trigger factor type chaperone family protein
Gene.10228	heat shock protein 81-2
Gene.10773	UDP-glucuronic acid decarboxylase 3
Gene.12641	farnesylcysteine lyase
Gene.13254	RAB GTPase homolog H1A
Gene.13498	dolichyl-diphosphooligosaccharide-protein glycosyltransferase 48kDa subunit family protein
P_sb	
Gene.10751	Oligosaccharyltransferase complex/magnesium transporter family protein
Gene.15071	Endosomal targeting BRO1-like domain-containing protein
Gene.18002	Calcineurin-like metallo-phosphoesterase superfamily protein
Gene.18707	zinc finger (C2H2 type) family protein
Gene.21415	D111/G-patch domain-containing protein
Gene.22540	forkhead-associated (FHA) domain-containing protein
Gene.25474	VIER F-box protein 1
Gene.25579	Cystathionine beta-synthase (CBS) family protein
Gene.28797	plastid developmental protein DAG
Gene.29200	DNAJ heat shock N-terminal domain-containing protein
Gene.29932	DNAJ heat shock N-terminal domain-containing protein
Gene.32110	purple acid phosphatase 9
Gene.32346	Auxin-responsive family protein
Gene.32826	pleckstrin homologue 1
Gene.33356	TCP domain protein 10
Gene.34467	Glycolipid transfer protein (GLTP) family protein
Gene.34588	Nucleotidyltransferase family protein
Gene.36522	SIN3 associated polypeptide P18
Gene.36862	WRKY DNA-binding protein 43
Gene.38065	mediator of RNA polymerase II transcription subunit
Gene.39476	mitochondrial processing peptidase alpha subunit
Gene.43435	PLANT HOMOLOGOUS TO PARAFIBROMIN
Gene.44305	TCP domain protein 16
Gene.45692	GTP-binding protein, HflX
Gene.47492	LisH/CRA/RING-U-box domains-containing protein

**Ss_Aa**	
Gene.48725	F-box family protein
Gene.48962	cyclase associated protein 1
Gene.50357	prohibitin 5
Gene.51187	RGA-like protein 3
Gene.51723	zinc finger (Ran-binding) family protein
Gene.52388	RNA polymerase II transcription mediator
Gene.52719	protease Do-like protein
Ss_Pc	
Gene.895	Bifunctional inhibitor/lipid-transfer protein/seed storage 2S albumin superfamily protein
Gene.3156	peroxin 7
Gene.3275	Coatomer, alpha subunit
Gene.3689	Plant neutral invertase family protein
Gene.4687	Duplicated homeodomain-like superfamily protein
Gene.5166	2-oxoglutarate (2OG) and Fe(II)-dependent oxygenase superfamily protein
Gene.6490	mediator of RNA polymerase II transcription subunit
Gene.7046	Pentatricopeptide repeat (PPR) superfamily protein
Gene.7470	Tudor/PWWP/MBT superfamily protein
Gene.7754	TCP domain protein 16
Gene.8081	Pentatricopeptide repeat (PPR) superfamily protein
Gene.9387	CRINKLY4 related 3
Gene.9924	HOPW1-1-interacting 2
Gene.11155	Galactosyltransferase family protein
Gene.11503	Adenine nucleotide alpha hydrolases-like superfamily protein
Gene.12008	RAC-like 10
Gene.13399	RNA-binding KH domain-containing protein
Aa_Pc	
Gene.4746	basic helix-loop-helix (bHLH) DNA-binding superfamily protein
Gene.9674	Protein kinase superfamily protein
Gene.11145	Rubisco methyltransferase family protein
Gene.13240	Calcineurin-like metallo-phosphoesterase superfamily protein
Gene.13616	zinc finger (C2H2 type) family protein
Gene.13931	homeobox knotted-like protein
Gene.15220	Pentatricopeptide repeat (PPR) superfamily protein
Gene.16446	Heavy metal transport/detoxification superfamily protein
Gene.16772	Endonuclease/exonuclease/phosphatase family protein
Gene.16773	ZIP metal ion transporter family
Gene.17142	fatty acid biosynthesis 1
Gene.17261	Tetratricopeptide repeat (TPR)-like superfamily protein
Gene.18872	proteinaceous RNase P 2
Gene.19653	TCP domain protein 10
Gene.20209	CRM family member 2
Gene.21626	Adenine nucleotide alpha hydrolases-like superfamily protein
Gene.22533	Major facilitator superfamily protein
Gene.22657	DNAJ heat shock N-terminal domain-containing protein
Gene.23039	mitochondrial processing peptidase alpha subunit
Gene.23386	TCP domain protein 16
Gene.24228	GTP-binding protein, HflX
Gene.24358	SAC domain-containing protein 8
Gene.24660	RNA-binding (RRM/RBD/RNP motifs) family protein with retrovirus zinc finger-like domain-containing protein
Gene.25405	F-box family protein
Gene.25444	Pentatricopeptide repeat (PPR) superfamily protein
Gene.26340	GRIP-related ARF-binding domain-containing protein 1
Gene.26541	RNA-binding (RRM/RBD/RNP motifs) family protein
Gene.27431	DNAJ heat shock family protein
Gene.27895	Pyridoxal phosphate (PLP)-dependent transferases superfamily protein
Gene.28553	glycine-rich protein
Gene.28601	arginine/serine-rich splicing factor 35
Gene.28913	RNA binding Plectin/S10 domain-containing protein

**Ss_Aa**	
Gene.29583	vesicle-associated membrane protein 711
Gene.29943	UvrB/UvrC domain protein (DUF3506)
Gene.30445	PHD finger family protein / bromo-adjacent homology (BAH) domain-containing protein
Gene.30715	RING/U-box superfamily protein
Gene.31174	Eukaryotic aspartyl protease family protein
Gene.31590	ankyrin-like1
Gene.32156	Pentatricopeptide repeat (PPR) superfamily protein
Gene.33129	prohibitin 5
Gene.33139	Thiamine pyrophosphate dependent pyruvate decarboxylase family protein
Gene.33746	Leucine-rich repeat (LRR) family protein
Gene.33956	cytochrome P450, family 71, subfamily B, polypeptide 13
Gene.34607	S-locus lectin protein kinase family protein
Gene.34643	cycling DOF factor 2
Gene.35597	Tetratricopeptide repeat (TPR)-like superfamily protein
Gene.35784	Y-family DNA polymerase H
Gene.35985	protein phosphatase X 2
Gene.36075	formin homolog 6
Aa_Ad	
Gene.21136	cellulose synthase like E1
Gene.10082	Pentatricopeptide repeat (PPR) superfamily protein
Gene.11683	uncoupling protein 5
Gene.2161	Pentatricopeptide repeat (PPR) superfamily protein
Gene.4259	membrane-anchored ubiquitin-fold protein 4
Gene.33430	histone deacetylase 3
Gene.4259	plant intracellular ras group-related LRR 7
Gene.34643	PHD finger family protein / bromo-adjacent homology (BAH) domain-containing protein
Gene.21398	decapping 5-like protein
Ad_Pc	
Gene.11148	MBOAT (membrane bound O-acyl transferase) family protein
Gene.9887	Pectin lyase-like superfamily protein
Gene.8299	homeobox knotted-like protein
Gene.18415	Heavy metal transport/detoxification superfamily protein
Gene.6039	myb-like HTH transcriptional regulator family protein
Gene.14722	TRICHOME BIREFRINGENCE-LIKE 13
Gene.7669	kinase with tetratricopeptide repeat domain-containing protein
Gene.7014	casein kinase I-like 5
Gene.9314	receptor like protein 25
Gene.17364	LIGHT-DEPENDENT SHORT HYPOCOTYLS-like protein (DUF640)
Gene.18302	PRA1 (Prenylated rab acceptor) family protein
Gene.13753	PLANT HOMOLOGOUS TO PARAFIBROMIN
Gene.8094	dehydratase family
Gene.5010	TCP domain protein 16
Gene.13661	Mitochondrial substrate carrier family protein
Gene.5410	GRIP-related ARF-binding domain-containing protein 1
Gene.133	glucan synthase-like 1
Gene.14579	Oxidoreductase family protein
Gene.18764	transferases, transferring glycosyl groups
Gene.3134	Tetratricopeptide repeat (TPR)-like superfamily protein
Gene.17448	tetraspanin5
Gene.14356	RING/U-box superfamily protein
Gene.9532	urophorphyrin methylase 1
Gene.11709	Pentatricopeptide repeat (PPR) superfamily protein
Gene.12019	farnesylcysteine lyase
Ss_Sl	
Gene.8393	NAC domain containing protein 6
Gene.13238	arabinogalactan protein 19
Gene.10272	Pyridoxal-5'-phosphate-dependent enzyme family protein
Gene.9868	UDP-glucosyl transferase 88A1
Gene.8590	NAD(P)-binding Rossmann-fold superfamily protein

**Ss_Aa**	
Sb_ad	
Gene.1683	RAD3-like DNA-binding helicase protein
Gene.27048	HVA22-like protein H (ATHVA22H)
Gene.8116	SPla/RYanodine receptor (SPRY) domain-containing protein
Gene.24283	SC35-like splicing factor 33
Gene.1125	SIN3-like 5
Gene.18777	RNA recognition motif (RRM)-containing protein
Gene.13847	mediator subunit 8
Gene.11731	peroxin 10
Gene.3680	pumilio 2
Gene.23892	alpha/beta-Hydrolases superfamily protein
Gene.26346	Uncharacterized conserved protein ycf60
Gene.13908	Mevalonate/galactokinase family protein
Gene.6365	Protein kinase superfamily protein
Gene.25116	WUSCHEL related homeobox 1
Gene.13239	dehydratase family
Gene.13169	proteolysis 1
Gene.7567	TCP domain protein 16
Gene.27182	Ribosomal protein L24e family protein
Gene.23340	alpha/beta-Hydrolases superfamily protein
Gene.27945	mRNA splicing factor, thioredoxin-like U5 snRNP
Gene.10628	heme oxygenase-like, multi-helical
Gene.24308	PQ-loop repeat family protein / transmembrane family protein
Aa_Sl	
Gene.1317	Rubisco methyltransferase family protein
Gene.3335	Protein kinase superfamily protein
Gene.33129	Calcineurin-like metallo-phosphoesterase superfamily protein
Gene.19674	pentatricopeptide (PPR) repeat-containing protein
Gene.31130	SPla/RYanodine receptor (SPRY) domain-containing protein
Gene.33411	cofactor assembly of complex C
Gene.27994	Phosphatidylinositol-4-phosphate 5-kinase family protein
Gene.11145	Endonuclease/exonuclease/phosphatase family protein
Gene.25405	ZIP metal ion transporter family
Gene.16671	cullin 3B
Gene.16971	DEA(D/H)-box RNA helicase family protein
Gene.31849	transcription factor IIIA
Gene.26587	DNAJ heat shock N-terminal domain-containing protein
Gene.19653	Tetratricopeptide repeat (TPR)-like superfamily protein
Gene.36153	Ctr copper transporter family
Gene.10458	Insulinase (Peptidase family M16) family protein
Gene.9044	SNF2 domain-containing protein / helicase domain-containing protein
Gene.13931	CRM family member 2
Gene.32244	Mov34/MPN/PAD-1 family protein
Gene.27895	mitochondrial processing peptidase alpha subunit
Gene.35078	WUSCHEL related homeobox 1
Gene.20736	Polynucleotidyl transferase, ribonuclease H-like superfamily protein
Gene.35751	membrane-anchored ubiquitin-fold protein 4
Gene.9096	Tudor/PWWP/MBT superfamily protein
Gene.13240	TCP domain protein 16
Gene.36006	Translation protein SH3-like family protein
Gene.31859	galacturonosyltransferase-like 2
Gene.35314	Ribosomal protein L2 family
Gene.1689	glucan synthase-like 1
Gene.9736	OSBP(oxysterol binding protein)-related protein 2B
Gene.20964	Potassium transporter family protein
Gene.23430	ubiquitin-associated (UBA)/TS-N domain-containing protein / octicosapeptide/Phox/Bemp1 (PB1) domain-containing protein
Gene.16772	arginine/serine-rich splicing factor 35
Gene.30762	Senescence/dehydration-associated protein-like protein

**Ss_Aa**	
Gene.35157	alpha/beta-Hydrolases superfamily protein
Gene.30942	glycine-rich protein
Gene.34643	PHD finger family protein / bromo-adjacent homology (BAH) domain-containing protein
Gene.24228	RING/U-box superfamily protein
Gene.27191	Polynucleotidyl transferase, ribonuclease H-like superfamily protein
Gene.1317	tudor/PWWP/MBT domain protein
Gene.21481	galacturonosyltransferase 14
Gene.33823	NAC domain containing protein 87
Gene.33746	Leucine-rich repeat (LRR) family protein
Gene.26119	P-loop containing nucleoside triphosphate hydrolases superfamily protein
Gene.29264	PRP38 family protein
Gene.22962	SMAD/FHA domain-containing protein
Gene.32057	homogentisate 1,2-dioxygenase
Gene.3286	potassium channel tetramerization domain-containing protein / pentapeptide repeat-containing protein
Gene.30715	protein phosphatase X 2
Gene.31722	D6 protein kinase
Gene.24408	RNA polymerase II fifth largest subunit, C
Gene.22991	damaged DNA binding 2
Gene.27842	RNA-binding (RRM/RBD/RNP motifs) family protein
Gene.31440	Copine (Calcium-dependent phospholipid-binding protein) family
Gene.21188	NAC domain containing protein 105
Ss_Ad	
Gene.2680	homeobox protein 32
Gene.4402	kinase family with ARM repeat domain-containing protein
Gene.4980	DNA double-strand break repair protein
Gene.6193	RNA-binding protein 1
Gene.8716	Trm112p-like protein
Gene.9466	Ribosomal protein L30/L7 family protein
Gene.9847	ubiquitin-specific protease 4
Gene.11635	glutamate receptor 2.9
Gene.11691	Mevalonate/galactokinase family protein
Gene.12371	Protein kinase superfamily protein
Gene.12394	Calcineurin-like metallo-phosphoesterase superfamily protein
Gene.13121	NADH-ubiquinone oxidoreductase-like protein
Gene.13498	conserved oligomeric Golgi complex subunit 4
Gene.14138	myb domain protein 16
Gene.14311	AGAMOUS-like 90
Gene.14445	Ribosomal protein S24e family protein

Supplemental Table 4

Supplemental Table 4. Functional annotation summary of candidate loci with elevated Ka/Ks ratios

**Ss-Aa**		
Functional Category	Description	Examples
Signal Transduction	Genes involved in cellular signaling pathways that respond to environmental or internal cues.	Protein kinases, GTPases
Environmental Stress Response	Genes that help organisms respond to environmental stresses such as heat, cold, and oxidative stress.	Heat shock proteins, ENTH/VHS family proteins
Metabolic Processes	Enzymes and transporters that are essential for metabolism.	UDP-glucuronic acid decarboxylase, MATE efflux family proteins
Developmental Processes	Genes that influence development.	Homeobox proteins, TCP domain proteins
Defense and Detoxification	Genes involved in the organism's defense mechanisms against pathogens or environmental toxins.	Metal ion transporters, peptidases
Pc-Sl		
Functional Category	Description	Examples
Protein Processing and Transport	Genes involved in the modification, folding, and transport of proteins within the cell.	Oligosaccharyltransferase complex/magnesium transporter family protein, Endosomal targeting BRO1-like domain-containing protein
Signal Transduction and Response	Genes that play roles in cellular signaling and response to environmental or developmental signals.	Calcineurin-like metallo-phosphoesterase superfamily protein, Auxin-responsive family protein, pleckstrin homologue 1
DNA Binding and Transcription Regulation	Genes involved in transcription regulation and DNA binding activities.	zinc finger (C2H2 type) family protein, WRKY DNA-binding protein 43, mediator of RNA polymerase II transcription subunit, RNA polymerase II transcription mediator
Enzymatic Function	Enzymes involved in biochemical reactions essential for various metabolic and developmental processes.	Cystathionine beta-synthase (CBS) family protein, purple acid phosphatase 9, mitochondrial processing peptidase alpha subunit
Protein Degradation	Genes associated with the ubiquitin-proteasome pathway and protein turnover.	VIER F-box protein 1, LisH/CRA/RING-U-box domains-containing protein, F-box family protein
Genetic and Cellular Integrity	Proteins that contribute to maintaining genetic and cellular structure integrity.	D111/G-patch domain-containing protein, SIN3 associated polypeptide P18, prohibitin 5, protease Do-like protein
Developmental Processes	Genes that influence development and growth.	forkhead-associated (FHA) domain-containing protein, plastid developmental protein DAG
Stress Response	Genes that help the organism respond to physiological and environmental stresses.	DNAJ heat shock N-terminal domain-containing proteins
Nucleic Acid Metabolism	Proteins involved in the synthesis and modification of nucleic acids.	Nucleotidyltransferase family protein, cyclase associated protein 1
GTPase Activity	Proteins involved in GTP binding and hydrolysis, which are crucial for cellular signaling and energy transactions.	GTP-binding protein, HflX
Transcription Factors	Proteins that regulate gene expression by binding to specific DNA sequences.	TCP domain protein 10, TCP domain protein 16
Metal Ion Transport and Binding	Proteins involved in the transport and binding of metal ions, essential for numerous cellular functions.	zinc finger (Ran-binding) family protein
Protein Regulation and Cell Cycle	Proteins involved in regulating other proteins and cellular processes including the cell cycle.	RGA-like protein 3, PLANT HOMOLOGOUS TO PARAFIBROMIN
Ss-Pc		
Functional Category	Description	Examples
Transcription Regulation	Genes involved in transcription regulation, impacting gene expression at various levels.	Mediator of RNA polymerase II transcription subunit
Seed Storage and Lipid Transfer	Proteins involved in storing energy and transferring lipids, critical for seed development and metabolism.	Bifunctional inhibitor/lipid-transfer protein/seed storage 2S albumin superfamily protein
Vesicular Transport	Proteins involved in the transport and sorting of proteins within cellular compartments.	Coatomer, alpha subunit
Carbohydrate Metabolism	Enzymes that catalyze the breakdown and synthesis of sugars, which are essential for energy production and structural components.	Plant neutral invertase family protein
Oxygen Transport and Reaction	Proteins that facilitate oxygen transport and catalyze reactions involving oxygen.	2-oxoglutarate (2OG) and Fe(II)-dependent oxygenase superfamily protein
Protein-DNA Interaction	Proteins that interact with DNA to affect chromatin structure and gene expression.	Duplicated homeodomain-like superfamily protein, Tudor/PWWP/MBT superfamily protein
RNA Processing and Regulation	Proteins involved in RNA splicing, stability, and regulation, critical for post-transcriptional control.	Pentatricopeptide repeat (PPR) superfamily protein (two instances), RNA-binding KH domain-containing protein
Transcription Factors	Proteins that bind to specific DNA sequences to control the transcription of genetic information.	TCP domain protein 16
Developmental Regulation	Proteins involved in developmental pathways.	CRINKLY4 related 3

**Ss-Aa**		
Protein-Protein Interaction	Proteins that mediate interactions between proteins, affecting various cellular processes including signaling and structural assembly.	HOPW1-1-interacting 2
Enzymatic Function	Proteins that catalyze biochemical reactions, essential for metabolic processes.	Galactosyltransferase family protein, Adenine nucleotide alpha hydrolases-like superfamily protein
Signal Transduction	Proteins involved in transmitting signals within the cell to respond to environmental changes.	RAC-like 10
Aa-Pc		
Functional Category	Description	Examples
Transcription Factors and DNA Binding	Genes involved in DNA binding and the regulation of transcription.	basic helix-loop-helix (bHLH) DNA-binding superfamily protein, homeobox knotted-like protein, TCP domain proteins
Signal Transduction	Proteins involved in cellular signaling pathways.	Protein kinase superfamily protein, GTP-binding protein, HflX, S-locus lectin protein kinase family protein
Metabolic Enzymes	Enzymes involved in essential metabolic processes.	Rubisco methyltransferase family protein, fatty acid biosynthesis 1, Thiamine pyrophosphate dependent pyruvate decarboxylase family protein, Pyridoxal phosphate (PLP)-dependent transferases superfamily protein
Ion Transport and Metal Handling	Proteins involved in the transport and handling of ions and metals.	Heavy metal transport/detoxification superfamily protein, ZIP metal ion transporter family, Major facilitator superfamily protein
Protein Processing and Cellular Stress Response	Proteins involved in processing other proteins and responding to cellular stress.	Calcineurin-like metallo-phosphoesterase superfamily protein, Endonuclease/exonuclease/phosphatase family protein, mitochondrial processing peptidase alpha subunit, DNAJ heat shock N-terminal domain-containing protein
RNA Binding and Processing	Proteins that bind RNA and are involved in its processing.	RNA-binding (RRM/RBD/RNP motifs) family protein, RNA binding Plectin/S10 domain-containing protein, Pentatricopeptide repeat (PPR) superfamily proteins
Protein Degradation and Ubiquitination	Proteins involved in tagging other proteins for degradation.	F-box family protein, RING/U-box superfamily protein
Cellular Structure and Transport	Proteins that contribute to cellular structure or are involved in vesicle transport.	ankyrin-like1, vesicle-associated membrane protein 711, formin homolog 6
Genome Maintenance and Repair	Proteins involved in maintaining genome integrity and repairing DNA damage.	UvrB/UvrC domain protein (DUF3506), Y-family DNA polymerase H
Regulatory and Developmental Proteins	Proteins that regulate other proteins and contribute to developmental processes.	GRIP-related ARF-binding domain-containing protein 1, cycling DOF factor 2
Specialized Enzymatic Functions	Enzymes with specific roles in biochemical pathways.	Eukaryotic aspartyl protease family protein, cytochrome P450, family 71, subfamily B, polypeptide 13

Supplemental Table 5

Supplemental Table 5. Leaf-developmental regulator transcripts recovered in the combined reference, with best-hit gene, family assignment, and percent identity.

transcript_id	swissprot	gene_symbol	protein_name	pident	evalue	family
transcript/181	Q9FGV1	ARF8	Auxin response factor 8	61	6.34e-169	ARF
transcript/525	Q9FGV1	ARF8	Auxin response factor 8	55	2.24e-287	ARF
transcript/1129	Q9FGV1	ARF8	Auxin response factor 8	71.6	5.20999999999999e-93	ARF
transcript/1491	Q9FGV1	ARF8	Auxin response factor 8	63.2	9.06e-166	ARF
transcript/1771	Q2LAJ3	ARF2A	Auxin response factor 2A	68.8	0	ARF
transcript/2444	Q9FGV1	ARF8	Auxin response factor 8	70.4	0	ARF
transcript/2506	Q9SJ56	BLH1	BEL1-like homeodomain protein 1	47.3	9.32e-72	BELL/BLH
transcript/2601	Q9FGV1	ARF8	Auxin response factor 8	61.4	1.38e-170	ARF
transcript/2789	Q9FGV1	ARF8	Auxin response factor 8	61.4	1.89e-184	ARF
transcript/3323	Q9SJ56	BLH1	BEL1-like homeodomain protein 1	54.3	1.19e-174	BELL/BLH
transcript/3341	Q9XED8	ARF9	Auxin response factor 9	59.5	2.06e-259	ARF
transcript/3857	Q9FGV1	ARF8	Auxin response factor 8	59.6	2.22999999999999e-68	ARF
transcript/3892	Q9FWS9	BLH3	BEL1-like homeodomain protein 3	50	7.57e-86	BELL/BLH
transcript/3912	Q9FGV1	ARF8	Auxin response factor 8	83.6	6.76e-170	ARF
transcript/4045	Q9FGV1	ARF8	Auxin response factor 8	55.1	8.28e-291	ARF
transcript/4574	Q2LAJ3	ARF2A	Auxin response factor 2A	68.3	0	ARF
transcript/4680	Q8L7G0	ARF1	Auxin response factor 1	47.1	1.76e-270	ARF
transcript/5107	Q94KL5	BLH4	BEL1-like homeodomain protein 4	80.2	3.52e-125	BELL/BLH
transcript/5635	Q9FGV1	ARF8	Auxin response factor 8	57.3	2.25e-290	ARF
transcript/6405	Q9SJ56	BLH1	BEL1-like homeodomain protein 1	56.8	8.02e-139	BELL/BLH
transcript/6516	Q9FGV1	ARF8	Auxin response factor 8	51.3	2.18999999999999e-255	ARF
transcript/7134	Q2LAJ3	ARF2A	Auxin response factor 2A	65.3	0	ARF
transcript/7672	Q94JM3	ARF2	Auxin response factor 2	65.7	0	ARF
transcript/8400	Q9FGV1	ARF8	Auxin response factor 8	68.7	0	ARF
transcript/8550	Q9FWS9	BLH3	BEL1-like homeodomain protein 3	49.7	2.98e-85	BELL/BLH
transcript/9363	Q9FGV1	ARF8	Auxin response factor 8	38.9	6.61e-76	ARF
transcript/10116	Q9XED8	ARF9	Auxin response factor 9	59.8	1.83e-265	ARF
transcript/10673	Q94KL5	BLH4	BEL1-like homeodomain protein 4	81.4	6.21e-128	BELL/BLH
transcript/10806	Q9SJ56	BLH1	BEL1-like homeodomain protein 1	53.4	8.14e-183	BELL/BLH
transcript/10895	Q9FGV1	ARF8	Auxin response factor 8	69.5	0	ARF
transcript/10899	O23661	ARF3	Auxin response factor 3	69.2	1.18999999999999e-197	ARF
transcript/10972	Q9SJ56	BLH1	BEL1-like homeodomain protein 1	58.1	1.18e-107	BELL/BLH
transcript/12210	Q9SJ56	BLH1	BEL1-like homeodomain protein 1	53.4	3.56e-183	BELL/BLH
transcript/12352	Q9SJJ3	BLH8	BEL1-like homeodomain protein 8	42.1	1.04e-79	BELL/BLH
transcript/12362	Q9SJ56	BLH1	BEL1-like homeodomain protein 1	51.1	4.95e-177	BELL/BLH
transcript/13722	Q8LPR5	TCP4	Transcription factor TCP4	47.9	1.21999999999999e-24	CIN-type TCP (TCP2/3/4/10/24)
transcript/14164	Q9XED8	ARF9	Auxin response factor 9	59.3	1.18e-261	ARF
transcript/14965	Q9SJ56	BLH1	BEL1-like homeodomain protein 1	57	1.94e-152	BELL/BLH
transcript/15692	Q8LPR5	TCP4	Transcription factor TCP4	48.4	2.30999999999999e-82	CIN-type TCP (TCP2/3/4/10/24)
transcript/16105	Q8LPR5	TCP4	Transcription factor TCP4	48.4	6.91e-82	CIN-type TCP (TCP2/3/4/10/24)

transcript_id	swissprot	gene_symbol	protein_name	pident	evalue	family
transcript/17548	Q38909	XTH28	Probable xyloglucan endotransglucosylase/hydrolase protein 28	64.2	9.5e-66	XTH
transcript/18456	Q8L7G0	ARF1	Auxin response factor 1	67.3	2.15e-254	ARF
transcript/19064	Q9ZPY6	ARF11	Auxin response factor 11	49.4	6.42e-202	ARF
transcript/19315	O23661	ARF3	Auxin response factor 3	47.7	8.9e-47	ARF
transcript/19506	Q9ZPY6	ARF11	Auxin response factor 11	57	1.35999999999999e-236	ARF
transcript/19529	Q9ZPY6	ARF11	Auxin response factor 11	56.9	1.68e-235	ARF
transcript/19571	Q8L7G0	ARF1	Auxin response factor 1	59.1	2.33e-257	ARF
transcript/20140	Q8L7G0	ARF1	Auxin response factor 1	70.7	0	ARF
transcript/20537	Q9XED8	ARF9	Auxin response factor 9	58.1	2.38e-240	ARF
transcript/21856	Q8LDS2	XTH27	Probable xyloglucan endotransglucosylase/hydrolase protein 27	48.2	1.96e-139	XTH
transcript/22090	O48818	EXPA4	Expansin-A4	41.8	6.96e-117	Expansin
transcript/22770	O04136		Homeobox protein knotted-1-like 3	81.2	4.41e-238	KNOX (other knotted-like)
transcript/23344	Q38827	IAA9	Auxin-responsive protein IAA9	49.6	8.84999999999999e-104	Aux/IAA
transcript/23730	Q8L7G0	ARF1	Auxin response factor 1	78.8	2.3e-220	ARF
transcript/24041	Q9XED8	ARF9	Auxin response factor 9	55.6	1.48999999999999e-234	ARF
transcript/24120	A0A067XRK9	XTH6	Xyloglucan endotransglucosylase protein 6	69.8	2.44e-77	XTH
transcript/24712	Q9S7W5	TCP13	Transcription factor TCP13	48.6	1.04e-26	CIN-type TCP (TCP2/3/4/10/24)
transcript/28463	Q9C9L2	TCP15	Transcription factor TCP15	94.1	3.49999999999999e-42	Other TCP
transcript/28512	Q9FWS9	BLH3	BEL1-like homeodomain protein 3	51.5	6.84e-105	BELL/BLH
transcript/31494	Q9FWS9	BLH3	BEL1-like homeodomain protein 3	51.2	1.06e-103	BELL/BLH
transcript/32014	Q9FMX2	TCP7	Transcription factor TCP7	66.1	5.27e-73	Other TCP
transcript/32095	O04136		Homeobox protein knotted-1-like 3	83.4	1.95e-245	KNOX (other knotted-like)
transcript/33635	O23661	ARF3	Auxin response factor 3	37.8	1.03999999999999e-68	ARF
transcript/34102	Q8LDS2	XTH27	Probable xyloglucan endotransglucosylase/hydrolase protein 27	49.8	4.01e-46	XTH
transcript/34851	Q9LZM8	BLH9	BEL1-like homeodomain protein 9	44.7	4.74e-122	BELL/BLH
transcript/35592	Q8LDS2	XTH27	Probable xyloglucan endotransglucosylase/hydrolase protein 27	73.9	4.16e-77	XTH
transcript/36491	Q8LPR5	TCP4	Transcription factor TCP4	47.8	3.63e-85	CIN-type TCP (TCP2/3/4/10/24)
transcript/37248	O48818	EXPA4	Expansin-A4	80.7	2.79e-86	Expansin
transcript/38867	Q9SJ56	BLH1	BEL1-like homeodomain protein 1	57.1	1.05e-130	BELL/BLH
transcript/39651	A0A067XRK9	XTH6	Xyloglucan endotransglucosylase protein 6	38.2	4.21e-127	XTH
transcript/44969	Q8LAL2	IAA26	Auxin-responsive protein IAA26	47.9	3.85999999999999e-74	Aux/IAA
transcript/45072	Q38909	XTH28	Probable xyloglucan endotransglucosylase/hydrolase protein 28	54.8	1.3e-117	XTH
transcript/46242	O22874	EXPA8	Expansin-A8	81.1	4.81999999999999e-146	Expansin
transcript/46509	Q8LAL2	IAA26	Auxin-responsive protein IAA26	47.9	4.63e-74	Aux/IAA
transcript/47718	Q9C9L2	TCP15	Transcription factor TCP15	79.8	1.13999999999999e-42	Other TCP
transcript/47825	Q38831	IAA13	Auxin-responsive protein IAA13	56.1	3.55e-67	Aux/IAA
transcript/48199	Q9C9L2	TCP15	Transcription factor TCP15	85.4	2.74999999999999e-42	Other TCP

transcript_id	swissprot	gene_symbol	protein_name	pident	evalue	family
transcript/49444	Q8LDS2	XTH27	Probable xyloglucan endotransglucosylase/hydrolase protein 27	60	4.5e-120	XTH
transcript/49899	Q9ZSY8	IAA27	Auxin-responsive protein IAA27	50.3	1.02e-90	Aux/IAA
transcript/50107	P48000	KNAT3	Homeobox protein knotted-1-like 3	80.1	1.87e-149	KNOX class II (KNAT3/4/5/7)
transcript/52062	Q38832	IAA14	Auxin-responsive protein IAA14	72	1.26e-109	Aux/IAA
transcript/52240	Q38909	XTH28	Probable xyloglucan endotransglucosylase/hydrolase protein 28	67.8	8.49e-163	XTH
transcript/52569	Q9S7W5	TCP13	Transcription factor TCP13	59.7	1.18e-18	CIN-type TCP (TCP2/3/4/10/24)
transcript/52722	Q38827	IAA9	Auxin-responsive protein IAA9	61.7	4.14e-141	Aux/IAA
transcript/53859	Q9FFD0	PIN5	Auxin efflux carrier component 5	70.5	3.67e-174	PIN
transcript/53903	O64647	TCP9	Transcription factor TCP9	43.7	3.86e-51	Other TCP
transcript/53941	Q38831	IAA13	Auxin-responsive protein IAA13	49.4	2.15e-60	Aux/IAA
transcript/54069	Q9LSD5	TCP20	Transcription factor TCP20	59	1.05e-73	Other TCP
transcript/55022	Q9FPQ8	KNAT7	Homeobox protein knotted-1-like 7	80.7	2.1e-159	KNOX class II (KNAT3/4/5/7)
transcript/55361	Q8LPR5	TCP4	Transcription factor TCP4	50.8	2.95e-11	CIN-type TCP (TCP2/3/4/10/24)
transcript/55630	Q9S7W5	TCP13	Transcription factor TCP13	41.6	7.51e-45	CIN-type TCP (TCP2/3/4/10/24)
transcript/56552	Q9LZM8	BLH9	BEL1-like homeodomain protein 9	47.6	9.51e-74	BELL/BLH
transcript/57520	P35694		Xyloglucan endotransglucosylase/hydrolase 2	83.3	6.03e-170	XTH
transcript/58147	Q9FMX2	TCP7	Transcription factor TCP7	66.1	6.97e-76	Other TCP
transcript/58331	Q9C554	EXPA1	Expansin-A1	83.2	6.29e-157	Expansin
transcript/58745	Q8LAL2	IAA26	Auxin-responsive protein IAA26	67	2.24e-68	Aux/IAA
transcript/59646	Q38909	XTH28	Probable xyloglucan endotransglucosylase/hydrolase protein 28	58.9	1.88e-123	XTH
transcript/60918	O48818	EXPA4	Expansin-A4	84	4.28999999999999e-159	Expansin
transcript/61185	Q8LF99	XTH6	Probable xyloglucan endotransglucosylase/hydrolase protein 6	76.2	2.66e-169	XTH
transcript/61437	Q38909	XTH28	Probable xyloglucan endotransglucosylase/hydrolase protein 28	61.2	1.08999999999999e-104	XTH
transcript/61583	Q9LSD5	TCP20	Transcription factor TCP20	50.9	2.89e-53	Other TCP
transcript/62014	O22874	EXPA8	Expansin-A8	80.8	3.1e-145	Expansin
transcript/62206	Q93VH6	GIF3	GRF1-interacting factor 3	60.8	4.73e-61	GIF/AN3
transcript/62481	O24407	IAA16	Auxin-responsive protein IAA16	68.3	1.28e-107	Aux/IAA
transcript/63351	A0A067XRK9	XTH6	Xyloglucan endotransglucosylase protein 6	76.3	7.99e-174	XTH
transcript/64212	G5DAC6	XTH1	Xyloglucan endotransglucosylase protein 1	67.1	5.95e-70	XTH
transcript/64442	Q38825	IAA7	Auxin-responsive protein IAA7	71.9	3.54e-103	Aux/IAA
transcript/64470	Q38825	IAA7	Auxin-responsive protein IAA7	72.2	1.06e-109	Aux/IAA
transcript/64843	G5DAC6	XTH1	Xyloglucan endotransglucosylase protein 1	69.4	6.95e-145	XTH

transcript_id	swissprot	gene_symbol	protein_name	pident	evalue	family
transcript/65159	Q8LC45	XTH33	Probable xyloglucan endotransglucosylase/hydrolase protein 33	66.9	1.06e-135	XTH
transcript/65291	Q38910	XTH23	Probable xyloglucan endotransglucosylase/hydrolase protein 23	76	5.57999999999999e-158	XTH
transcript/65577	G5DAC6	XTH1	Xyloglucan endotransglucosylase protein 1	71.7	4.47e-149	XTH
transcript/65712	O22874	EXPA8	Expansin-A8	80.2	3.21e-147	Expansin
transcript/66405	G5DAC6	XTH1	Xyloglucan endotransglucosylase protein 1	71.7	3.39e-149	XTH
transcript/66843	Q9XFB0	YAB2	Putative axial regulator YABBY 2	55.9	2.84e-60	YABBY
transcript/67216	Q9C9L2	TCP15	Transcription factor TCP15	84.1	4.94e-28	Other TCP
transcript/67723	Q9XFB0	YAB2	Putative axial regulator YABBY 2	37.1	2.12e-17	YABBY
transcript/67995	Q5VRD1	IAA1	Auxin-responsive protein IAA1	63.8	9.59e-51	Aux/IAA
transcript/344	Q9SJ56	BLH1	BEL1-like homeodomain protein 1	40.7	1.04999999999999e-40	BELL/BLH
transcript/353	Q9FGV1	ARF8	Auxin response factor 8	59.5	3.43e-166	ARF
transcript/880	Q9FGV1	ARF8	Auxin response factor 8	55.1	1.03e-287	ARF
transcript/944	Q9FGV1	ARF8	Auxin response factor 8	47.7	3.63e-102	ARF
transcript/1540	Q9FGV1	ARF8	Auxin response factor 8	67.8	2.93e-57	ARF
transcript/1755	Q9SE43	REV	Homeobox-leucine zipper protein REVOLUTA	85.1	8.19e-211	HD-ZIP III (REV/PHB/PHV)
transcript/1830	Q9FGV1	ARF8	Auxin response factor 8	69.6	4.27e-83	ARF
transcript/2195	Q9FGV1	ARF8	Auxin response factor 8	39.7	9.39e-77	ARF
transcript/2493	Q9SJ56	BLH1	BEL1-like homeodomain protein 1	73.8	4.14e-23	BELL/BLH
transcript/2577	Q94KL5	BLH4	BEL1-like homeodomain protein 4	79.8	2.28e-130	BELL/BLH
transcript/2966	Q9ZTX9	ARF4	Auxin response factor 4	63.2	4.1e-314	ARF
transcript/4407	Q9SJ56	BLH1	BEL1-like homeodomain protein 1	53.6	8.43e-185	BELL/BLH
transcript/4907	Q9SJ56	BLH1	BEL1-like homeodomain protein 1	62.3	1.03e-154	BELL/BLH
transcript/4953	Q8L7G0	ARF1	Auxin response factor 1	42.9	3e-143	ARF
transcript/5261	Q9C6B8	PIN1	Auxin efflux carrier component 1	55.2	3.52e-207	PIN
transcript/5406	Q9SJ56	BLH1	BEL1-like homeodomain protein 1	53.5	7.24e-185	BELL/BLH
transcript/6357	Q8LPR5	TCP4	Transcription factor TCP4	40	2.36e-24	CIN-type TCP (TCP2/3/4/10/24)
transcript/6714	Q9ZPY6	ARF11	Auxin response factor 11	74.7	6.49e-100	ARF
transcript/6951	Q9FGV1	ARF8	Auxin response factor 8	56.2	1.59e-289	ARF
transcript/7126	Q9XED8	ARF9	Auxin response factor 9	59.5	4.86e-261	ARF
transcript/8629	O04136		Homeobox protein knotted-1-like 3	97.9	2.46e-156	KNOX (other knotted-like)
transcript/9074	Q8LPR5	TCP4	Transcription factor TCP4	54.4	3.11e-96	CIN-type TCP (TCP2/3/4/10/24)
transcript/9203	Q8L7G0	ARF1	Auxin response factor 1	71	0	ARF
transcript/9343	Q2LAJ3	ARF2A	Auxin response factor 2A	64.8	8.54e-310	ARF
transcript/11164	Q8LPR5	TCP4	Transcription factor TCP4	42.5	4.99e-54	CIN-type TCP (TCP2/3/4/10/24)
transcript/12319	O81001	GRF1	Growth-regulating factor 1	42.1	5.39e-80	GRF
transcript/12688	Q9LZM8	BLH9	BEL1-like homeodomain protein 9	42.1	1.05e-37	BELL/BLH
transcript/13174	Q9C6B8	PIN1	Auxin efflux carrier component 1	70.4	9.64e-291	PIN

transcript_id	swissprot	gene_symbol	protein_name	pident	evalue	family
transcript/14310	Q9C9L2	TCP15	Transcription factor TCP15	94.1	6.71999999999999e-42	Other TCP
transcript/16148	Q8L8A6	GRF5	Growth-regulating factor 5	54.8	3.57e-20	GRF
transcript/17590	Q9FPQ8	KNAT7	Homeobox protein knotted-1-like 7	80.7	1.98e-158	KNOX class II (KNAT3/4/5/7)
transcript/19607	Q38827	IAA9	Auxin-responsive protein IAA9	60	1.97e-135	Aux/IAA
transcript/20276	Q8LPR5	TCP4	Transcription factor TCP4	50.8	4.09e-12	CIN-type TCP (TCP2/3/4/10/24)
transcript/20495	Q8L7G0	ARF1	Auxin response factor 1	71	0	ARF
transcript/20582	Q9ZSY8	IAA27	Auxin-responsive protein IAA27	52.2	8.71e-92	Aux/IAA
transcript/20680	Q9FMX2	TCP7	Transcription factor TCP7	67.1	2.66e-76	Other TCP
transcript/22790	O24407	IAA16	Auxin-responsive protein IAA16	69.5	1.23e-107	Aux/IAA
transcript/23202	Q8LF99	XTH6	Probable xyloglucan endotransglucosylase/hydrolase protein 6	75.3	4.89999999999999e-172	XTH
transcript/26186	O22152	YAB1	Axial regulator YABBY 1	67.1	3.76999999999999e-92	YABBY
transcript/27241	Q8GW46	YAB5	Axial regulator YABBY 5	72.4	1.46e-78	YABBY
transcript/27336	Q5VRD1	IAA1	Auxin-responsive protein IAA1	54.8	1.78999999999999e-54	Aux/IAA
transcript/27652	G5DAC6	XTH1	Xyloglucan endotransglucosylase protein 1	70.6	5.6e-147	XTH
transcript/825	Q9FGV1	ARF8	Auxin response factor 8	60.8	1.59e-168	ARF
transcript/1291	Q9FGV1	ARF8	Auxin response factor 8	57.7	9.27e-143	ARF
transcript/1578	Q9FGV1	ARF8	Auxin response factor 8	60.1	9.63e-164	ARF
transcript/1925	Q9FGV1	ARF8	Auxin response factor 8	60.1	3.04e-164	ARF
transcript/2839	Q6AWY4	GRF5	Growth-regulating factor 5	49.7	8.58e-39	GRF
transcript/4797	Q9FGV1	ARF8	Auxin response factor 8	55.3	9.15e-289	ARF
transcript/6970	Q8L7G0	ARF1	Auxin response factor 1	48.2	1.16e-276	ARF
transcript/7221	Q9SJ56	BLH1	BEL1-like homeodomain protein 1	53.5	2.77e-185	BELL/BLH
transcript/7414	Q2LAJ3	ARF2A	Auxin response factor 2A	67.8	0	ARF
transcript/7615	Q2LAJ3	ARF2A	Auxin response factor 2A	67.7	0	ARF
transcript/7943	Q9SJ56	BLH1	BEL1-like homeodomain protein 1	61.7	5.38e-143	BELL/BLH
transcript/9590	Q9ZTX9	ARF4	Auxin response factor 4	61.7	6.63999999999999e-244	ARF
transcript/9826	Q9FGV1	ARF8	Auxin response factor 8	39.3	2.25e-76	ARF
transcript/11832	Q93VH6	GIF3	GRF1-interacting factor 3	51.4	8.88e-24	GIF/AN3
transcript/11848	Q9ZPY6	ARF11	Auxin response factor 11	52.7	6.3e-82	ARF
transcript/12933	Q8L7G0	ARF1	Auxin response factor 1	55.7	1.13e-298	ARF
transcript/13784	Q9FWS9	BLH3	BEL1-like homeodomain protein 3	50.9	4.28e-90	BELL/BLH
transcript/14802	Q38827	IAA9	Auxin-responsive protein IAA9	76.1	5.47e-15	Aux/IAA
transcript/14811	Q9XED8	ARF9	Auxin response factor 9	59.2	2.82e-258	ARF
transcript/18263	Q9ZTX9	ARF4	Auxin response factor 4	63.7	6.57e-181	ARF
transcript/18908	Q9FGV1	ARF8	Auxin response factor 8	59.3	1.45e-179	ARF
transcript/18970	Q94KL5	BLH4	BEL1-like homeodomain protein 4	65.2	3.58e-97	BELL/BLH
transcript/19192	Q38909	XTH28	Probable xyloglucan endotransglucosylase/hydrolase protein 28	63	2.6e-65	XTH
transcript/20829	Q9ZPY6	ARF11	Auxin response factor 11	56.9	7.97e-236	ARF
transcript/20858	Q9XED8	ARF9	Auxin response factor 9	52.3	3.04e-141	ARF
transcript/20960	Q9XED8	ARF9	Auxin response factor 9	58.7	1.02e-210	ARF

transcript_id	swissprot	gene_symbol	protein_name	pident	evalue	family
transcript/21168	Q9ZPY6	ARF11	Auxin response factor 11	57	1.27e-236	ARF
transcript/21462	Q8LDS2	XTH27	Probable xyloglucan end otransglucosylase/hydrol ase protein 27	43	1.05e-130	XTH
transcript/21791	Q9XED8	ARF9	Auxin response factor 9	53.3	1.51e-215	ARF
transcript/22722	Q9XED8	ARF9	Auxin response factor 9	54.5	1.16e-216	ARF
transcript/22832	Q8L7G0	ARF1	Auxin response factor 1	28.7	7.91e-53	ARF
transcript/24552	Q9C6B8	PIN1	Auxin efflux carrier component 1	70.5	3.19999999999999e-287	PIN
transcript/25270	Q9M0I2	EXPB3	Expansin-B3	75.7	1.48e-93	Expansin
transcript/28104	O04136		Homeobox protein knotted-1-like 3	83.2	4.6e-182	KNOX (other knotted-like)
transcript/28765	Q9C518	TCP8	Transcription factor TCP8	46.3	2.28999999999999e-67	Other TCP
transcript/29111	Q9FPQ8	KNAT7	Homeobox protein knotted-1-like 7	78.4	7.06e-27	KNOX class II (KNAT3/4/5/7)
transcript/30811	Q9FWS9	BLH3	BEL1-like homeodomain protein 3	52.6	3.14e-108	BELL/BLH
transcript/31585	O48818	EXPA4	Expansin-A4	88	3.47e-49	Expansin
transcript/31777	Q8LDS2	XTH27	Probable xyloglucan end otransglucosylase/hydrol ase protein 27	72.2	1.36e-76	XTH
transcript/32454	Q38909	XTH28	Probable xyloglucan end otransglucosylase/hydrol ase protein 28	63	2.72e-66	XTH
transcript/33982	Q8LPR5	TCP4	Transcription factor TCP4	50.8	3.64e-30	CIN-type TCP (TCP2/3/4/10/24)
transcript/35850	Q8LAL2	IAA26	Auxin-responsive protein IAA26	49.3	3.95e-77	Aux/IAA
transcript/36391	Q9ZSY8	IAA27	Auxin-responsive protein IAA27	50.9	6.67e-94	Aux/IAA
transcript/37127	Q9C554	EXPA1	Expansin-A1	84	2.16e-157	Expansin
transcript/39076	Q94KL5	BLH4	BEL1-like homeodomain protein 4	46.4	1.01e-09	BELL/BLH
transcript/44238	O24407	IAA16	Auxin-responsive protein IAA16	69.4	1.77e-110	Aux/IAA
transcript/44409	Q38831	IAA13	Auxin-responsive protein IAA13	48.2	1.48e-57	Aux/IAA
transcript/44900	Q9FPQ8	KNAT7	Homeobox protein knotted-1-like 7	80.1	1.57e-156	KNOX class II (KNAT3/4/5/7)
transcript/45366	Q9S7W5	TCP13	Transcription factor TCP13	81.1	2.56999999999999e-42	CIN-type TCP (TCP2/3/4/10/24)
transcript/45515	Q9C554	EXPA1	Expansin-A1	78.2	5.81e-142	Expansin
transcript/45858	Q94KL5	BLH4	BEL1-like homeodomain protein 4	46.4	7.81e-10	BELL/BLH
transcript/46599	Q8GW46	YAB5	Axial regulator YABBY 5	71.8	1.24e-74	YABBY
transcript/47264	O22874	EXPA8	Expansin-A8	82.8	1.3e-146	Expansin
transcript/47471	Q38831	IAA13	Auxin-responsive protein IAA13	57.7	2.29e-51	Aux/IAA
transcript/47716	Q9LSD5	TCP20	Transcription factor TCP20	58.3	1.99e-77	Other TCP
transcript/47811	Q8LF99	XTH6	Probable xyloglucan end otransglucosylase/hydrol ase protein 6	77.9	1.6e-69	XTH
transcript/48201	Q9LSD5	TCP20	Transcription factor TCP20	62.5	1.72e-61	Other TCP
transcript/48533	Q9FFD0	PIN5	Auxin efflux carrier component 5	71.2	9.16999999999999e-176	PIN
transcript/48586	Q5QMM3	WOX8	WUSCHEL-related homeobox 8	73.7	3.43e-79	WOX
transcript/49157	Q9FMX2	TCP7	Transcription factor TCP7	65.6	2.57e-75	Other TCP
transcript/49171	Q9FFD0	PIN5	Auxin efflux carrier component 5	71	1.16e-175	PIN
transcript/49407	Q9FMX2	TCP7	Transcription factor TCP7	65.6	2.43e-75	Other TCP

transcript_id	swissprot	gene_symbol	protein_name	pident	evalue	family
transcript/49438	Q8LF99	XTH6	Probable xyloglucan endotransglucosylase/hydrolase protein 6	77.7	5.88999999999999e-170	XTH
transcript/49976	Q84JS6	KNAT6	Homeobox protein knotted-1-like 6	62.9	4.90999999999999e-104	KNOX class I (STM/BP/KNAT2/6)
transcript/51352	G5DAC6	XTH1	Xyloglucan endotransglucosylase protein 1	77	1.17e-77	XTH
transcript/51814	Q6AWY4	GRF5	Growth-regulating factor 5	41.7	5.98e-52	GRF
transcript/52201	Q38825	IAA7	Auxin-responsive protein IAA7	57.2	2.41e-32	Aux/IAA
transcript/52780	O24407	IAA16	Auxin-responsive protein IAA16	68.1	4.01e-107	Aux/IAA
transcript/53756	Q9LSD5	TCP20	Transcription factor TCP20	51.5	2.03999999999999e-54	Other TCP
transcript/53782	Q8LF99	XTH6	Probable xyloglucan endotransglucosylase/hydrolase protein 6	50.8	1.71e-97	XTH
transcript/54176	G5DAC6	XTH1	Xyloglucan endotransglucosylase protein 1	69.3	2.94e-149	XTH
transcript/54720	Q8GW46	YAB5	Axial regulator YABBY 5	70.8	1.60999999999999e-67	YABBY
transcript/55291	Q5QMM3	WOX8	WUSCHEL-related homeobox 8	77	6.89e-77	WOX
transcript/55295	G5DAC6	XTH1	Xyloglucan endotransglucosylase protein 1	70.4	6.3e-146	XTH
transcript/55331	G5DAC6	XTH1	Xyloglucan endotransglucosylase protein 1	70.6	2.09e-149	XTH
transcript/55771	G5DAC6	XTH1	Xyloglucan endotransglucosylase protein 1	72.1	4.49e-146	XTH
transcript/55879	O48818	EXPA4	Expansin-A4	81.4	1.84e-162	Expansin
transcript/536	Q9FGV1	ARF8	Auxin response factor 8	60.1	3.49999999999999e-165	ARF
transcript/1236	Q9FGV1	ARF8	Auxin response factor 8	40.9	9.45e-168	ARF
transcript/1426	Q9FGV1	ARF8	Auxin response factor 8	59.6	6.55e-166	ARF
transcript/1647	Q9FGV1	ARF8	Auxin response factor 8	41.7	9.86999999999999e-180	ARF
transcript/1769	Q9FGV1	ARF8	Auxin response factor 8	48	3.58e-232	ARF
transcript/2014	Q2LAJ3	ARF2A	Auxin response factor 2A	67.7	0	ARF
transcript/2214	Q9SE43	REV	Homeobox-leucine zipper protein REVOLUTA	82.6	0	HD-ZIP III (REV/PHB/PHV)
transcript/2981	Q2LAJ3	ARF2A	Auxin response factor 2A	67.8	0	ARF
transcript/5255	O23661	ARF3	Auxin response factor 3	67.9	1.02999999999999e-190	ARF
transcript/6452	Q94KL5	BLH4	BEL1-like homeodomain protein 4	80.2	7.48e-126	BELL/BLH
transcript/7035	Q9C6B8	PIN1	Auxin efflux carrier component 1	55.8	3.27e-206	PIN
transcript/7971	Q9SJ56	BLH1	BEL1-like homeodomain protein 1	51.4	6.43e-176	BELL/BLH
transcript/9461	Q9S7W5	TCP13	Transcription factor TCP13	69	7.08e-26	CIN-type TCP (TCP2/3/4/10/24)
transcript/9900	Q9M0I2	EXPB3	Expansin-B3	75.7	1.97999999999999e-94	Expansin
transcript/12530	Q8L7G0	ARF1	Auxin response factor 1	33	2.14e-79	ARF
transcript/12988	Q9C6B8	PIN1	Auxin efflux carrier component 1	64.4	6.9e-259	PIN
transcript/15027	O81001	GRF1	Growth-regulating factor 1	40.3	1.54e-79	GRF
transcript/15394	A0A067XRK9	XTH6	Xyloglucan endotransglucosylase protein 6	31.5	8.98e-108	XTH
transcript/16310	O04136		Homeobox protein knotted-1-like 3	86.2	8.63e-252	KNOX (other knotted-like)

transcript_id	swissprot	gene_symbol	protein_name	pident	evalue	family
transcript/19654	Q9C518	TCP8	Transcription factor TCP8	82.6	1.09e-46	Other TCP
transcript/19814	Q9SJ56	BLH1	BEL1-like homeodomain protein 1	39.3	3.81e-41	BELL/BLH
transcript/22012	O81001	GRF1	Growth-regulating factor 1	52.3	1.53999999999999e-30	GRF
transcript/24255	Q94KL5	BLH4	BEL1-like homeodomain protein 4	46.4	1.06e-09	BELL/BLH
transcript/24287	Q38827	IAA9	Auxin-responsive protein IAA9	43.9	1.97999999999999e-61	Aux/IAA
transcript/26761	Q9C9L2	TCP15	Transcription factor TCP15	80.2	6.85e-44	Other TCP
transcript/27476	A0A067XRK9	XTH6	Xyloglucan endotransglucosylase protein 6	43.4	1.13e-138	XTH
transcript/29273	Q38827	IAA9	Auxin-responsive protein IAA9	49.4	2.09999999999999e-104	Aux/IAA
transcript/30846	Q9FGV1	ARF8	Auxin response factor 8	47.7	1.89e-42	ARF
transcript/30985	O64647	TCP9	Transcription factor TCP9	53.3	3.11999999999999e-62	Other TCP
transcript/33593	Q8L8A6	GRF5	Growth-regulating factor 5	71.2	2.30999999999999e-48	GRF
transcript/34513	Q6AWY4	GRF5	Growth-regulating factor 5	46.5	7.72e-79	GRF
transcript/34858	P48000	KNAT3	Homeobox protein knotted-1-like 3	79.7	1.34e-148	KNOX class II (KNAT3/4/5/7)
transcript/35883	Q38831	IAA13	Auxin-responsive protein IAA13	41	1e-31	Aux/IAA
transcript/36022	Q9S7W5	TCP13	Transcription factor TCP13	43.5	5.47e-44	CIN-type TCP (TCP2/3/4/10/24)
transcript/36549	Q6AWX7	GRF12	Growth-regulating factor 12	54.6	2.79e-26	GRF
transcript/36646	Q9M0I2	EXPB3	Expansin-B3	75.9	1.5e-138	Expansin
transcript/37089	Q9C554	EXPA1	Expansin-A1	84	1.58e-157	Expansin
transcript/37112	G5DAC6	XTH1	Xyloglucan endotransglucosylase protein 1	55.3	1.38e-106	XTH
transcript/37396	O64647	TCP9	Transcription factor TCP9	52.4	8.12e-75	Other TCP
transcript/38203	Q38909	XTH28	Probable xyloglucan endotransglucosylase/hydrolase protein 28	72.2	5.51e-79	XTH
transcript/38994	A0A067XRK9	XTH6	Xyloglucan endotransglucosylase protein 6	77	2.14e-151	XTH
transcript/38997	Q6AWY4	GRF5	Growth-regulating factor 5	39.4	1.01e-58	GRF
transcript/39858	Q9ZSY8	IAA27	Auxin-responsive protein IAA27	54.9	1.94e-99	Aux/IAA
transcript/40097	O48818	EXPA4	Expansin-A4	83.6	4.85e-158	Expansin
transcript/40257	Q8LPR5	TCP4	Transcription factor TCP4	48.9	3.03e-36	CIN-type TCP (TCP2/3/4/10/24)
transcript/40638	O22152	YAB1	Axial regulator YABBY 1	65	3.41e-88	YABBY
transcript/41146	G5DAC6	XTH1	Xyloglucan endotransglucosylase protein 1	55.3	2.53e-107	XTH
transcript/41220	O48818	EXPA4	Expansin-A4	54.5	1.14e-90	Expansin
transcript/41517	O22152	YAB1	Axial regulator YABBY 1	60.5	2.08e-80	YABBY
transcript/41670	O24407	IAA16	Auxin-responsive protein IAA16	67.7	1.60999999999999e-106	Aux/IAA
transcript/42183	O48818	EXPA4	Expansin-A4	88.1	8.67e-166	Expansin
transcript/43423	Q9SJL9	XTH32	Probable xyloglucan endotransglucosylase/hydrolase protein 32	80.9	2.26e-186	XTH
transcript/43586	Q38909	XTH28	Probable xyloglucan endotransglucosylase/hydrolase protein 28	68.1	3.63e-164	XTH

transcript_id	swissprot	gene_symbol	protein_name	pident	evalue	family
transcript/45167	Q9SJL9	XTH32	Probable xyloglucan endotransglucosylase/hydrolase protein 32	73.2	8.01e-153	XTH
transcript/46758	Q9C554	EXPA1	Expansin-A1	78.2	1.05e-142	Expansin
transcript/47290	P35694		Xyloglucan endotransglucosylase/hydrolase 2	81.2	5.97e-89	XTH
transcript/47393	Q8LC45	XTH33	Probable xyloglucan endotransglucosylase/hydrolase protein 33	64.2	6.16e-143	XTH
transcript/48022	Q38910	XTH23	Probable xyloglucan endotransglucosylase/hydrolase protein 23	73	9.99e-160	XTH
transcript/48047	Q9XFB0	YAB2	Putative axial regulator YABBY 2	48.5	1.05e-48	YABBY
transcript/48858	Q9XFB0	YAB2	Putative axial regulator YABBY 2	67.1	6.0899999999999e-30	YABBY
transcript/64016	Q9ZTX9	ARF4	Auxin response factor 4	72.5	4.55e-17	ARF
transcript/1457	Q9FGV1	ARF8	Auxin response factor 8	59.8	6.41e-167	ARF
transcript/2836	Q9FGV1	ARF8	Auxin response factor 8	55.4	1.72e-285	ARF
transcript/3229	Q9SJ56	BLH1	BEL1-like homeodomain protein 1	46.5	2.24e-77	BELL/BLH
transcript/3459	Q9SE43	REV	Homeobox-leucine zipper protein REVOLUTA	85	2.67e-296	HD-ZIP III (REV/PHB/PHV)
transcript/4110	Q2LAJ3	ARF2A	Auxin response factor 2A	82.1	1.49e-213	ARF
transcript/5008	Q2LAJ3	ARF2A	Auxin response factor 2A	35.6	6.94e-38	ARF
transcript/5154	Q9FGV1	ARF8	Auxin response factor 8	70.7	0	ARF
transcript/5176	Q9FGV1	ARF8	Auxin response factor 8	39.7	1.1e-76	ARF
transcript/5207	Q9FGV1	ARF8	Auxin response factor 8	70.9	0	ARF
transcript/5598	Q94JM3	ARF2	Auxin response factor 2	90.2	3.25e-152	ARF
transcript/6091	Q94JM3	ARF2	Auxin response factor 2	44.5	1.12e-83	ARF
transcript/7198	Q94KL5	BLH4	BEL1-like homeodomain protein 4	79.8	8.93e-132	BELL/BLH
transcript/7827	Q9C6B8	PIN1	Auxin efflux carrier component 1	55.3	5.91e-207	PIN
transcript/9703	Q9S7W5	TCP13	Transcription factor TCP13	69	7.09e-26	CIN-type TCP (TCP2/3/4/10/24)
transcript/9792	Q8LPR5	TCP4	Transcription factor TCP4	49.8	3.14999999999999e-84	CIN-type TCP (TCP2/3/4/10/24)
transcript/9953	Q9XED8	ARF9	Auxin response factor 9	57.9	8.80999999999999e-242	ARF
transcript/9989	Q8L7G0	ARF1	Auxin response factor 1	70.6	0	ARF
transcript/10106	Q94KL5	BLH4	BEL1-like homeodomain protein 4	87.3	6.68e-116	BELL/BLH
transcript/10629	Q9S7W5	TCP13	Transcription factor TCP13	76.4	5.15e-26	CIN-type TCP (TCP2/3/4/10/24)
transcript/12123	Q9FWS9	BLH3	BEL1-like homeodomain protein 3	51.5	2.45e-106	BELL/BLH
transcript/12286	Q9S7W5	TCP13	Transcription factor TCP13	50	2.74e-26	CIN-type TCP (TCP2/3/4/10/24)
transcript/12827	Q8L7G0	ARF1	Auxin response factor 1	31.3	2.77e-76	ARF
transcript/12949	Q9ZPY6	ARF11	Auxin response factor 11	72.4	9.99e-95	ARF
transcript/17030	O04136		Homeobox protein knotted-1-like 3	83.5	4.48e-248	KNOX (other knotted-like)
transcript/17285	O23661	ARF3	Auxin response factor 3	38.8	1.54e-65	ARF
transcript/17676	O04136		Homeobox protein knotted-1-like 3	81.3	2.32e-244	KNOX (other knotted-like)
transcript/17884	Q9FWS9	BLH3	BEL1-like homeodomain protein 3	34.2	6.44e-10	BELL/BLH
transcript/30189	Q9FFD0	PIN5	Auxin efflux carrier component 5	70.3	4.1e-171	PIN
transcript/31030	Q38827	IAA9	Auxin-responsive protein IAA9	52.4	4.44e-105	Aux/IAA
transcript/33262	Q9FFD0	PIN5	Auxin efflux carrier component 5	69.1	5.44999999999999e-170	PIN

transcript_id	swissprot	gene_symbol	protein_name	pident	evalue	family
transcript/35116	O64647	TCP9	Transcription factor TCP9	61.3	1.40999999999999e-61	Other TCP
transcript/37093	Q9S7W5	TCP13	Transcription factor TCP13	40.2	1.47e-46	CIN-type TCP (TCP2/3/4/10/24)
transcript/37256	Q9C554	EXPA1	Expansin-A1	84.5	5.01e-156	Expansin
transcript/37983	Q9LZM8	BLH9	BEL1-like homeodomain protein 9	47.2	4.95e-72	BELL/BLH
transcript/38115	Q38831	IAA13	Auxin-responsive protein IAA13	56.3	2.27999999999999e-68	Aux/IAA
transcript/38719	Q38831	IAA13	Auxin-responsive protein IAA13	56.8	3.77e-68	Aux/IAA
transcript/38748	Q9FMX2	TCP7	Transcription factor TCP7	67.1	9.45e-77	Other TCP
transcript/40812	O24407	IAA16	Auxin-responsive protein IAA16	69.4	8.52e-110	Aux/IAA
transcript/40866	Q9LSD5	TCP20	Transcription factor TCP20	52.1	1.96e-53	Other TCP
transcript/41202	O48818	EXPA4	Expansin-A4	82.6	5.17e-162	Expansin
transcript/43130	Q9LSD5	TCP20	Transcription factor TCP20	61.7	7.11e-11	Other TCP
transcript/44226	Q93VH6	GIF3	GRF1-interacting factor 3	59.9	6.74e-59	GIF/AN3
transcript/44564	O22152	YAB1	Axial regulator YABBY 1	58.3	8.41e-75	YABBY
transcript/46272	Q9M0I2	EXPB3	Expansin-B3	29.6	1.07999999999999e-24	Expansin
transcript/46828	Q5QMM3	WOX8	WUSCHEL-related homeobox 8	76.6	3.73e-79	WOX
transcript/46936	O48818	EXPA4	Expansin-A4	87.4	3.16e-165	Expansin
transcript/47272	Q5QMM3	WOX8	WUSCHEL-related homeobox 8	77.2	4.19e-80	WOX
transcript/48261	P35694		Xyloglucan endotransglucosylase/hydrolase 2	83.3	6.76999999999999e-172	XTH
transcript/48949	O64647	TCP9	Transcription factor TCP9	62.8	1.54999999999999e-24	Other TCP

Supplemental Table 6

Supplemental Table 6. Ortholog pairs evaluated and retained after Ks filtering in each pairwise species comparison.

**Comparison**	**Phylogenetic relationship**	**RBH pairs evaluated**	**Excluded Ks < 0.05**	**Excluded Ks ≥ 2.0**	**Retained (%)**	**Ka/Ks > 1 (%)**	**Median Ks**
A. aethusifolius vs. *A. dioicus*	Congeneric (*Aruncus*)	4,571	2,751	89	1,731 (37.9)	86 (4.97)	0.032
S. splendens vs. *S. lucida*	Congeneric (*Spiraea*)	3,784	1,341	110	2,333 (61.7)	111 (4.76)	0.066
P. caespitosum vs. *S. lucida*	Intergeneric, within subclade	7,864	544	115	7,205 (91.6)	259 (3.59)	0.140
S. splendens vs. *P. caespitosum*	Intergeneric, within subclade	5,021	331	78	4,612 (91.9)	147 (3.19)	0.142
A. aethusifolius vs. *P. caespitosum*	Intergeneric, between subclades	7,988	251	116	7,621 (95.4)	232 (3.04)	0.226
A. dioicus vs. *P. caespitosum*	Intergeneric, between subclades	6,110	160	88	5,862 (95.9)	158 (2.70)	0.227
A. aethusifolius vs. *S. lucida*	Intergeneric, between subclades	6,566	169	96	6,301 (96.0)	221 (3.51)	0.254
S. lucida vs. *A. dioicus*	Intergeneric, between subclades	4,920	100	91	4,729 (96.1)	133 (2.81)	0.256
S. splendens vs. *A. aethusifolius*	Intergeneric, between subclades	4,138	101	68	3,969 (95.9)	107 (2.70)	0.259
S. splendens vs. *A. dioicus*	Intergeneric, between subclades	3,485	81	76	3,328 (95.5)	90 (2.70)	0.260

Percentages in the Retained column are of RBH pairs evaluated; percentages in the Ka/Ks > 1 column are of retained pairs.

Median Ks is calculated over pairs with Ks ≤ 1, matching Figure 7.
